# Tobacco smoking differently influences cell types of the innate and adaptive immune system—indications from CpG site methylation

**DOI:** 10.1186/s13148-016-0249-7

**Published:** 2016-08-03

**Authors:** Mario Bauer, Beate Fink, Loreen Thürmann, Markus Eszlinger, Gunda Herberth, Irina Lehmann

**Affiliations:** 1 Department of Environmental Immunology, Helmholtz Centre for Environmental Research-UFZ, Leipzig, 04318 Germany; 2 Division of Endocrinology and Nephrology, University of Leipzig, Leipzig, 04103 Germany

**Keywords:** DNA methylation, CpG, Tobacco smoking, Blood, Biomarker, GPR15

## Abstract

**Background:**

Tobacco smoke is worldwide one of the main preventable lifestyle inhalative pollutants causing severe adverse health effects. Epidemiological studies revealed association of tobacco smoking with epigenetic changes at single CpGs in blood. However, the biological relevance of the often only marginal methylation changes remains unclear.

**Results:**

Comparing genome-wide changes in CpG methylation of three recently reported epidemiological datasets, two obtained on whole blood and one on peripheral blood mononuclear cells (PBMCs), it becomes evident that the majority of methylation changes (86.7 and 93.3 %) in whole blood account for changes in granulocytes. Analyzing, in more detail, seven highly significant reported smoking-induced methylation changes at single CpGs in different blood cell types of healthy volunteers (*n* = 32), we confirmatively found a strong cell-type specificity. Two CpGs in *GFI1* and *F2RL3* were significantly hypomethylated in granulocytes (−11.3 %, *p* = 0.001; −8.7 %, *p* = 0.001, respectively) but not in PBMCs of smokers while two CpGs in *CPOX* and *GPR15* were found to be hypomethylated in PBMC (−4.3 %, *p* = 0.003; −4.2 %, *P* = 0.009, respectively) and their subtypes of GPR15 non-expressing (−3.2 %, *p* = 0.027; −2.5 %, *p* = 0.032, respectively) and smoking-evoked GPR15 expressing T cells (−15.8 %, *p* < 0.001; −13.8 %, *p* = 0.018, respectively) but not in granulocytes. In contrast, cg05575921 within *AHRR* was hypomethylated in every analyzed cell type of smokers, but with a different degree. Both, hypomethylation at cg05575921 in granulocytes (−55.2 % methylation change in smokers, *p* < 0.001) and the frequency of GPR15+ T cells (9.8–37.1 % in smokers), possessing a specific hypomethylation at cg19859270, were strongly associated with smoking behavior at individual level and could therefore serve as valuable biomarkers indicating a disturbed homeostasis in smokers.

In contrast to the reported long-term persistent methylation changes in adult smokers after cessation, the hypomethylation at cg05575921 in prenatally tobacco smoke-exposed children (*n* = 13) from our LINA cohort was less stable and disappeared already within 2 years after birth.

**Conclusions:**

Studying cell type-specific methylation changes provides helpful information regarding the biological relevance of epigenetic modifications. Here, we could show that smoking differently affects both cells of the innate and adaptive immune systems.

**Electronic supplementary material:**

The online version of this article (doi:10.1186/s13148-016-0249-7) contains supplementary material, which is available to authorized users.

## Background

Tobacco smoke is the main preventable lifestyle risk factor causing adverse health effects including the development of chronic obstructive pulmonary disease (COPD) and lung cancer. A better understanding of the mechanisms, leading to the harm by smoking, may yield strategies to manage disease risk. Genome-wide epigenetic studies identified strong associations between tobacco smoke exposure and changes in DNA methylation at single sites (CpG) in whole blood or isolated peripheral blood mononuclear cells (PBMCs) [1–12]. It was found that methylation changes at CpGs are dynamic but reversible, indicating a potential mechanism of reestablishment of DNA status after cessation [5, 6, 12]. Moreover, methylation changes at single CpGs were observed to persist after cessation [2]. In addition to smoking-related DNA methylation changes in adult active smokers, there is strong evidence from newborn studies that maternal smoking during pregnancy, especially during the second and third trimesters, can contribute to an altered DNA methylation pattern in cord blood [13–21] which may also persist for years after birth [22].

Altogether, population-based studies [14–21] have consistently shown that active tobacco smoking leads to methylation shift at CpG level in whole blood. However, whether and how these minor methylation changes in whole blood associated with tobacco smoking may cause or contribute to disease pathology remains elusive. Furthermore, none of the epidemiological studies extended their research to clarify the cellular distribution of methylation changes over different cell types of the whole blood.

In our previous study, we recently have shown that active tobacco smoking may lead to an expansion of a specialized T cell type accounting for a methylation shift at cg19859270 in composed blood samples like whole blood or PBMCs in adults [23]. With the present study, we pursued the goal to prove our hypothesis that the tobacco smoking-induced methylation changes at CpGs in whole blood are dissimilarly distributed among different cell types indicating different protection mechanisms against the harm of smoking. We therefore studied the cell-specific methylation profiles in smokers and non-smokers for five frequently reported CpGs from earlier epidemiological studies [4, 6, 8]. Based on the obtained results, our additional aim was to find biomarkers suitable to identify homeostasis-disturbing effect by tobacco smoking at the subject level.

## Results

### Distribution of smoking-induced CpG methylation changes in leukocytes

**Fig. 1:**
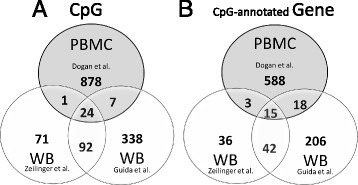
Venn diagram illustrating intersections of tobacco smoking-evoked methylation changes at single CpGs (**a**) and their annotated genes (**b**) of three different reports using genomic DNA isolated from whole blood (WB, Zeilinger et al., *n* = 1793; Guida et al., *n* = 745) or from separated peripheral blood mononuclear cells (PBMC, Dogan et al., *n* = 111) of adult probands. The origin of data sets is indicated

Recent epidemiological reports, describing epigenetic changes in whole blood of adults by active tobacco smoking, are mainly based on DNA extraction from whole blood, except one report exclusively focusing on DNA from isolated PBMCs [4]. When comparing tobacco smoking-evoked methylation changes at CpG sites identified in PBMC [4] with those in whole blood [6, 8], only a minority of significant CpGs in PBMC were concomitantly found in whole blood (32 out of 910, see Fig. 1a and Additional file 1). A similar result was also observed for CpG-annotated genes (Fig. 1b). Thus, it seems most likely that data created from whole blood, in first instance, indicate events occurring in granulocytes, thereby underestimating smoking-related methylation changes in PBMCs.

**Fig. 2:**
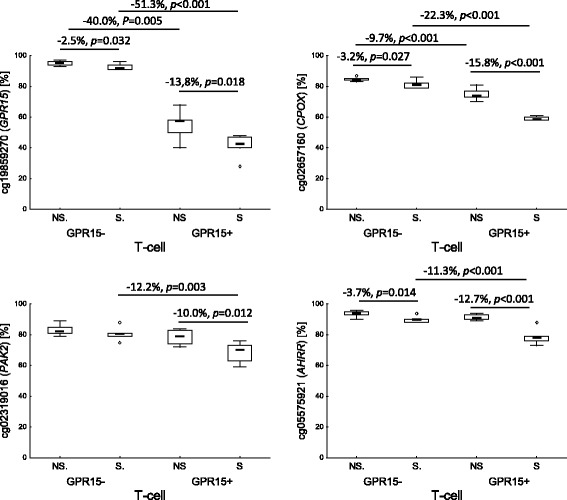
Distribution of methylation changes at single CpGs by tobacco smoking in GPR15− and GPR15+ T cells of whole blood. Highlighted are significant methylation changes expressed in percentage. *NS* non-smoker, *S* smoker; *p*, *P* statistical significance by Student’s *t* test (*p*) or Mann-Whitney *U* test (*P*)

To prove this hypothesis, we exemplarily explored the cell-specific methylation profiles in adult smokers and non-smokers (*n* = 32) for five frequently reported CpGs in recent epidemiological studies (cg02657160 in *CPOX*, cg09935388 in *GFI1*, cg05575921 in *AHRR*, cg03636183 in *F2RL3*, and cg19859270 in *GPR15*). Our methylation analyses were performed in whole blood, granulocytes, PBMC, CD3+ T cells expressing (GPR15+ T cell) or not (GPR15− T cell) the smoking-evoked GPR15 surface membrane protein. For all five CpGs, smoking-induced methylation changes were mainly found in that leukocyte population as predicted from the comparison of the smoking-related methylation change in PBMC versus whole blood, based on recent reported data (see Additional file 2: Table S2). In detail, the two analyzed CpGs in *GFI1* and *F2RL3* were significantly hypomethylated in granulocytes of smokers (−11.2 % [confidence interval, −5.0 to −17.4 %], *p* = 0.0013; −8.7 % [−3.9 to −13.5 %], *p* = 0.0013; respectively) but not in PBMCs (Table 1). In contrast, the two CpGs in *CPOX* and *GPR15* were found to be smoking-induced hypomethylated in PBMC (−4.3 % [−1.8 to −6.8 %], *p* = 0.003; −4.2 % [−1.2 to −7.1 %], *P* = 0.009; respectively) and their subtypes of GPR15− T cells (−3.2 % [−0.4 to −5.9 %], *p* = 0.027; −2.5 % [−0.3 to −4.7 %], *p* = 0.032; respectively) and GPR15+ T cells (−15.8 % [−12.2 to −19.4 %], *p* < 0.001; −13.8 % [−3.0 to −24.7 %], *p* = 0.018; respectively) but not in granulocytes. Cg05575921 in the *AHRR* gene body was smoking-induced hypomethylated both in granulocytes and PBMC but with an about threefold greater extent in granulocytes (−55.2 % [−48.5 to −61.9 %], *p* < 0.001; −15.7 % [−10.0 to −21.3 %], *p* = 0.005; respectively, see Additional file 3: Figure S4 for methylation changes at cg19859270 [*GPR15*], cg03636183 [*F2RL3*], and cg05575921 [*AHRR*]). Consistently, an increased AHRR gene expression was found in granulocytes as well as PBMC of smokers compared to non-smokers (Fig. 2).

**Table 1:** Tobacco smoking-associated methylation change at single CpG in different leukocyte populations of the blood

Gene	*AHRR*	*CPOX*	*F2RL3*	*GFI1*	*GPR15*	*PAICS*	*PAK2*
CpG	cg05575921	cg02657160	cg03636183	cg09935388	cg19859270	cg13086586	cg02319016
Leukocyte population							
Whole blood (*n* = 10, each group)							
NS (%)^a^	89.8 ± 5.9	85.5 ± 2.7	77.1 ± 8.6	66.6 ± 4.9	93.9 ± 1.3	12.7 ± 1.6	91.3 ± 3.7
S (%)^a^	68.2 ± 8.3	83.4 ± 2.0	71.8 ± 6.9	62.2 ± 3.7	92.9 ± 1.0	13.9 ± 1.9	90.0 ± 2.2
∆meth (%)^d^	*−21.6* (*−14.8*; *−28.4*)	−2.1 (0.1; −4.3)	−5.3 (0.4; −10.9)	−*4.4* (−*0.3*; −*8.5*)	−*1.0* (*0.1*; −*2.1*)	1.2 (2.8; −0.4)	−1.3 (1.6; −4.2)
*p* value^b^	2.8E−04	6.0E−02	6.6E−02	3.6E−02	4.7E−02^c^	1.4E−01	3.5E−01
Granulocyte (*n* = 10, each group)							
NS (%)^a^	85.2 ± 7.3	85.3 ± 1.4	75.4 ± 5.5	52.9 ± 5.8	96.0 ± 0.5	12.4 ± 1.3	93.0 ± 1.5
S (%)^a^	30.0 ± 7.0	84.6 ± 1.7	66.7 ± 4.2	41.7 ± 7.3	95.8 ± 0.6	11.8 ± 2.8	92.3 ± 2.5
∆meth (%)^d^	−*55.2* (−*48.5*; −*61.9*)	−0.7 (0.8; −2.2)	−*8.7* (−*3.9*; −*13.5*)	−*11.2* (−*5.0*; −*17.4*)	−0.2 (0.3; −0.7)	−0.6 (1.5; −2.7)	−0.7 (1.2; −2.6)
*p* value^b^	1.2E−12	3.3E−01	1.3E−03	1.3E−03	4.3E−01^c^	1.0E+00^c^	4.5E−01
PBMC (*n* = 6, each group)							
NS (%)^a^	92.2 ± 3.3	84.3 ± 1.6	82.0 ± 3.2	86.2 ± 2.9	93.0 ± 1.1	11.3 ± 3.1	87.2 ± 3.2
S (%)^a^	76.5 ± 5.2	80.0 ± 2.2	84.5 ± 8.1	83.8 ± 3.2	88.8 ± 3.1	12.5 ± 3.0	86.7 ± 0.5
∆meth (%)^d^	−*15.7* (−*10.0*; −*21.3*)	−*4.3 *(−*1.8*; −*6.8*)	2.5 (10.4; −5.4)	−2.3 (1.6; −6.2)	−*4.2* (−*1.2*; −*7.1*)	1.2 (5.1; −2.8)	−0.5 (2.4; −3.4)
*p* value^b^	5.0E−03^c^	3.0E−03	5.0E−01	2.1E−01	9.4E−03^c^	4.6E−01^c^	3.7E−01^c^
CD3+GPR15− T cell (*n* = 6, each group)							
NS (%)^a^	93.7 ± 2.1	84.5 ± 1.4	85.5 ± 6.6	87.7 ± 3.7	95.2 ± 1.5	11.7 ± 3.3	83.0 ± 3.5
S (%)^a^	90.0 ± 2.2	81.3 ± 2.7	86.0 ± 6.8	88.5 ± 2.1	92.7 ± 2.0	13.2 ± 5.2	80.7 ± 4.2
∆meth (%)^d^	−*3.7 *(−*0.9*; −*6.4*)	−*3.2* (−*0.4*; −*5.9*)	0.5 (9.1; −8.1)	0.8 (4.7; −3.0)	−*2.5* (−*0.3*; −*4.7*)	1.5 (7.1; −4.1)	−2.3 (2.7; −7.3)
*p* value^b^	1.4E−02	2.7E−02	9.0E−01	6.4E−01	3.2E−02	5.6E−01	3.2E−01
CD3+GPR15+ T cell (*n* = 6, each group)							
NS (%)^a^	91.3 ± 1.9	74.8 ± 3.8	83.7 ± 6.6	87.5 ± 1.4	55.2 ± 9.4	12.8 ± 3.4	78.5 ± 4.8
S (%)^a^	78.7 ± 5.0	59.0 ± 1.3	86.2 ± 6.8	88.7 ± 2.5	41.3 ± 7.4	17.2 ± 8.6	68.5 ± 6.4
∆meth (%)^d^	−*12.7 *(−*7.8*;−*17.6*)	−*15.8* (−*12.2*;−*19.4*)	2.5 (11.1;−6.1)	1.2 (3.8;−1.4)	−*13.8* (−*3.0*;−*24.7*)	4.3 (12.7;−4.0)	−*10.0* (−*2.7*;−*17.3*)
*p* value^b^	1.8E−04	2.0E−06	5.1E−01^c^	3.4E−01	1.8E−02	2.2E−01^c^	1.2E−02

Significant differences between smokers (S) and non-smokers (NS) are in italics (*p* < 0.05)

^a^Mean

^b^Student’s *t* test

^c^Mann-Whitney *U* test

^d^Limit of 95 % confidence interval in parentheses

### Smoking-induced CpG methylation changes in T cells

Recently, we have reported that the specific hypomethylation in the gene body of *GPR15* at cg19859270 is evoked by the expansion of GPR15-expressing T cells in smokers. While less than 10 % of peripheral CD3+ T cells express the GPR15 protein on their surface, smoking may induce an excess of this cell population up to 40 %. To examine whether further methylation changes in PBMC also rely on the tobacco smoking specific expansion of this cell type, we analyzed smoking-related methylation changes for two highly significant hence top-ranked CpGs (cg13086586 in *PAICS*; cg02319016 in *PAK2*), exclusively reported in PBMC, together with cg05575921 (*AHRR*), cg02657160 (*CPOX*), and cg19859270 (*GPR15*) which were described differentially methylated in smoker both in studies on PBMC and whole blood. Smoking-induced methylation changes in T cells were found for the four CpGs in *AHRR*, *GPR15*, *CPOX*, and *PAK2* but not for the CpG in *PAICS* (Table 1)*.* While the CpG methylation changes in *AHRR*, *GPR15*, and *CPOX* were found both in GPR15− T cells (−3.7 % [−0.9 to −6.4], *p* = 0.014; −2.5 % [−0.3 to −4.7], *p* = 0.032; −3.2 % [−0.4 to −5.9], *p* = 0.027; respectively) and GPR15+ T cells (−12.7 % [−7.8 to −17.6], *p* < 0.001; −13.8 % [−3.0 to −24.7], *p* = 0.018; −15.8 % [−12.2 to −19.4], *p* < 0.001, respectively), changes for *PAK2* were only present in GPR15+ T cells (−10.0 % [−2.7 to −17.3], *p* = 0.012). However, the degree of methylation change was dissimilarly distributed between the two T cell types (Fig. 2). The evoked hypomethylation in smokers was much less pronounced in GPR15− T cells (<4 % difference in methylation) than in GPR15+ T cells (10.0 to 15.8 % difference in methylation). Thus, beside cg19859270 in *GPR15,* the GPR15+ T cell subpopulation may also account for further smoking-induced methylation changes observed in PBMC. Interestingly, beside cg19859270 in *GPR15* gene body*,* the chromosomal adjacent CpG site cg02657160 in *CPOX* may serve as an additional cell type-specific CpG site for GPR15+ T cells since this CpG was significantly hypomethylated in GPR15+ T cells (*p* < 0.001), irrespective of smoking behavior.

### Biomarkers for tobacco smoking

**Fig. 3:**
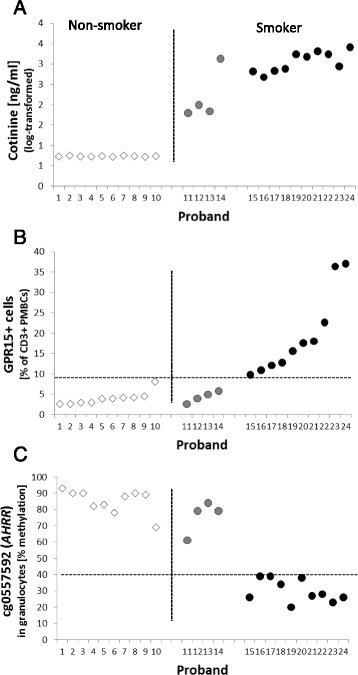
Cellular biomarkers associated with tobacco smoking. Subjects were separated into non-smoker (*white diamond*), occasional smoker (*gray circle*), and smoker (*black circle*) by both a questionnaire and cotinine level in the blood (**a**). Separately, the content of GPR15+ CD3+ T cells in blood (**b**) and the degree of methylation at the CpG site cg05575921 within *AHRR* in granulocytes (**c**) perfectly indicate the systemic effect of active tobacco smoking on blood cell types

To find out the best biomarker describing the systemic perturbation of homeostasis by tobacco smoking, we compared the flow-cytometric analyzed frequency of smoking-evoked GPR15+ T cells (CD3+) with the degree of methylation at the most conspicuous CpG site cg05575921 (*AHRR*) in granulocytes and the blood concentration of the nicotine metabolite cotinine at individual level. As indicated in Fig. 3, active smokers could be identified by (i) an elevated blood cotinine level (Fig. 3a), (ii) a frequency of GPR15+ T cells above 9 % (Fig. 3b), or (iii) a methylation degree below 40 % at cg05575921 (*AHRR)* in granulocytes (Fig. 3c). Both frequency of GPR15+ T cells and cg05575921 methylation did not correlate as in non-smokers (Spearman *r*_S_ = −0.56, *p* = 0.0931) as in smokers (*r*_S_ = −0.42, *p* = 0.22, see Additional file 4: Figure S3). However, the interindividual range of the amount of GPR15+ T cell (9.8–37.1 %) is twofold higher compared to cg05575921 methylation level (20–39 %) in smokers. Thus, for clinical purpose, the amount of GPR15+ T cells might better reflect the individual responses toward smoking exposure. Based on discontinuous distribution of the amount of GPR15+ T cell, smoking subjects may be segregated into high-affected (>30 % GPR15+ T cells) and low-affected (<26 % GPR15+ T cells).

**Fig. 4:**
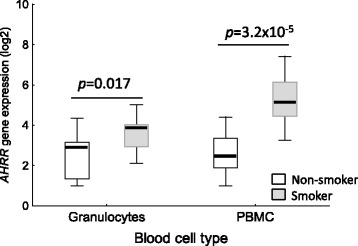
Smoking-induced *AHRR* expression in different cell types of the blood. The gene expression was increased in granulocytes as well as in PBMC from tobacco smokers (*n* = 14) in comparison to non-smoker (*n* = 10). *P*, Student’s *t* test

Gene expression data were not considered as a biomarker. However, similar to the functional cg19859270, a hypomethylated cg05575921 site in smokers is associated with an increased *AHRR* gene expression both in granulocytes (2.1-fold, *p* = 0.017) and in PBMC (6.3-fold, *p* < 0.001), thus, the cg05575921 might also belong to functional CpG sites (Fig. 4).

### Tobacco smoke-related CpG methylation pattern in early childhood

**Fig. 5:**
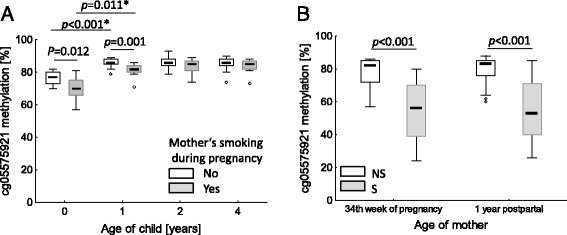
Influence of maternal smoking during pregnancy (*n* = 13) on time course of methylation at cg05575921 within *AHRR* in the child from birth until the age of 4 years (*n* = 20) (**a**) in comparison to the degree of smoking-related methylation changes in mothers during pregnancy and 1 year postpartal (**b**). Due to drop out probes, the number of tobacco smoke-exposed children during pregnancy over time is 13, 13, 11, and 8 for the ages of 0, 1, 2, and 3 years, respectively. Number of non-exposed children was always 20. *p Kruskal-Wallis ANOVA by ranks, *p* Student’s *t* test, *P* Mann-Whitney *U* test

The CpG site cg05575921 (*AHRR*) was reported to be hypomethylated in cord blood of children whose mother had sustained smoked during the two last trimesters of pregnancy. To evaluate a putative long-lasting effect of smoking during prenatal period, we followed the methylation pattern of this CpG in children from birth on up to the age of 4 years in whole blood. Children whose mothers smoked during pregnancy were hypomethylated at this CpG at birth (−6.0 % [CI, −1.4 to −10.6 %] of methylation, *P =* 0.012) and at the age of 1 year (−4.1 % [CI, −1.5 to −6.7 %], *p* = 0.001) but not thereafter (Fig. 5a). Interestingly, the hypomethylation at cg05575921 at birth disappeared in children of never-smoking mothers already at the age of 1 year (9.1 % [CI, 6.9 to 11.3 %] increase in methylation, *p* < 0.001), whereby in children of mothers who smoked during pregnancy, a degree of hypomethylation remained in spite of similar increment of methylation after the first year of life (11.0 % [6.3 to 15.7 %] increase in methylation, *p* = 0.011). At the individual level, cg05575921 (*AHRR)* failed to discriminate newborn with respect to the smoking exposure during pregnancy. At birth, only approximately 30 % (4 out of 13) of in utero exposed newborns have shown methylation levels below the lowest degree of methylation of 70 % which was found in unexposed newborns. The degree of methylation at cg05575921 in mothers did not change between pregnancy and 1 year postpartal (Fig. 5b). However, the degree of methylation in cord blood correlated with that in maternal blood from the 34th pregnancy week both in tobacco smoking-non-exposed (Spearman correlation *r*_*S*_ = 0.46, *p* = 0.045) and tobacco smoking-exposed (*r*_*S*_ = 0.63, *p* = 0.020) mother-child pairs.

### Influence of cigarette smoke extract on cg05575921 *(AHRR)* methylation in vitro

To verify if active tobacco smoking may directly impair the methylation at cg05575921in *AHRR*, we investigated the response of granulocytes as well as PBMCs isolated from adult non-smokers to aqueous cigarette smoke extract (CSE) in vitro (*n* = 3) following an 48-h exposure. However, CSE exposure did not change the methylation at cg05575921 in *AHRR* (see Additional file 5: Figure S2).

## Discussion

### Distribution of smoking-associated CpG among white blood cells

One major aim of this study was to show that smoking-related methylation changes observed in whole blood or PBMC are rather caused by activation/expansion of specific cell types than by minor methylation changes in each cell type.

Recently, with the smoking-evoked GPR15+ T cells, possessing a cell type-specific hypomethylation at cg19859270 within the gene body of *GPR15*, we identified such a specific cell type accounting for the minor methylation change at cg19859270 in whole blood or PBMC samples of smokers [23]. It has been hypothesized that an up-regulation of GPR15 could explain to some extent the health hazard of smoking with regard to chronic inflammatory diseases [24].

Thus, we further assumed that all methylation changes at single CpGs by tobacco smoking might be unequally distributed among the leukocyte population of the blood. To prove this, we chose several highly significant hence top-ranked CpGs repeatedly highlighted in recent studies, based on isolated DNA from whole blood [6] and PBMC [4]. As expected, we could show for the first time, by comparing data from these two recent reports and by our own experimental data based on separated cell populations, that smoking-induced methylation changes in blood are strongly cell type-specific. As shown for cg19859270 (*GPR15*), even a methylation difference in blood of around 2 % by smoking may be of strong biological relevance, if this methylation is caused by expansion of a specific cell type, namely GPR15+ T cells, involved in inflammation and disease pathology.

Interestingly, in all cases where the main smoking-induced methylation change at single CpG was expected in PBMC (cg19859270, *GPR15*; cg02657160, *CPOX*; cg02319016, *PAK2*), the greatest methylation change was found even in the GPR15+ T cells of smokers. Thus, it can be supposed that similar to the reported cg19859270 in the *GPR15* gene [23], the smoking-induced expansion of GPR15+ T cells may be responsible for further single methylation changes identified in whole blood or PBMC samples in smokers.

In contrast to other CpG sites showing cell type-specific methylation changes in smokers, the hypomethylation of cg05575921 within the *AHRR* gene was found in different cell types. The most pronounced methylation change emerged in granulocytes (−55.2 % compared to non-smoker) followed by PBMC (−15.7 %) and GPR15+ T cells (−12.7 %). Since GPR15+ T cells are only a small subset of PBMC (about 6–28 %), it can be assumed that monocytes additionally account for the methylation change of about 16 % at cg05575921 in PBMC. Smoking-induced methylation changes at cg05575921 in antigen-presenting cell types like EBV-immortalized lymphoblasts (corresponding to B cell) and alveolar macrophages may confirm this assumption [10]. The dissimilar effect size of methylation changes at cg05575921 by tobacco smoking furthermore discounts the previously discussed assumption of a stem cell origin of methylation changes.

Comparing smoking-induced changes in CpG methylation of whole blood with buccal samples, it was recently found that hypomethylated top-ranked CpGs (*n* = 19) were common to both tissues [25]. This might evoke the assumption that there are really existing CpG sites which are affected by smoking exposure irrespective of the tissue or cell type. However, because of (i) the absence of expected T cell-specific cg19859270 in *GPR15* [26], (ii) the presence of mainly granulocyte-specific top-ranked CpG sites (in *AHRR*, *F2RL3*, *GFI1* and others), and (iii) the huge number of different smoking-affected CpGs s between buccal and blood samples (see Additional file 6: Figure S1), this observation seems more likely to rely on expected contamination of buccal samples with leukocytes, especially granulocytes [27], than on a common smoking-induced hypomethylation at identical CpG sites both in leukocytes and buccal epithelial cells. Additionally, because of top-ranked CpGs in aryl-hydrocarbon receptor (AHR)-induced genes (*CYP1A1*, *CYP1B1*) in buccal samples, not conspicuous in whole blood, the concomitant presence of hypomethylated CpGs in AHR repressor gene (*AHRR*) in epithelial cells would not have been expected. A similar conclusion could also be drawn comparing isolated PBMC with whole blood. Due to contamination of PBMC preparations with granulocytes (3 ± 2 %, according to the manufacturer’s preparation instruction), the most significant CpGs of whole blood were expectedly found in PBMC (i.e., cg05575921 in *AHRR*, cg09935388 in *GFI1*, and cg03636183 *F2RL3*). Vice versa, the cell type-specific hypomethylation at cg19859270 (*GPR15*) of GPR15+ T cells (approximately 2–4 % of T cells in smokers) was found in whole blood of smokers.

### Cellular biomarker for tobacco smoking effects in blood

Based on epidemiological studies, a variety of CpG sites have been identified as potential candidates for monitoring current and life-time tobacco smoke exposure, among them several sites in *F2RL3*, cg05575921 in *AHRR* as well as cg19859270 in *GPR15*. The cg05575921 in *AHRR* was the CpG with the highest effect size [11, 12, 20] and has additionally become of special interest, since its methylation level was found to be changed already after a quite short period of smoking. Philibert and colleagues postulated that the extent of *AHRR* methylation at cg0557921 in PBMC might be a potential biomarker for the initiation of smoking because it shows a significant hypomethylation in nascent smokers who had an one pack-year exposure [11]. However, in that report, the methylation level at one pack-year exposure showed a broad overlapping range toward that of non-smokers indicating the impossibility of discrimination of subjects at the individual level by the cg0557921 methylation level in PBMC.

In contrast to the reported results at a population level, we intended to identify biomarkers suitable to identify tobacco smoke effects at the subject level. In this study, we have shown that the methylation level at cg05575921 in granulocytes as well as the amount of GPR15+ T cells may serve as highly sensitive and specific biomarkers in blood indicating a disturbed homeostasis by tobacco smoking at the subject level.

Comparing both biomarkers, it was evident that the amount of GPR15+ T cells much better reflects the individual variation in response to smoking than cg05575921 methylation level in granulocytes. It should be noted that it was not our intension to identify a biomarker correlating with the duration or the level of tobacco smoke exposure. In a century of personalized medicine, we rather focused on specific qualitative signs for disturbed homeostasis by tobacco smoking at an individual level. If GPR15+ T cells are activated and expanded as a consequence of lung inflammation and if the occurrence of this cell population in peripheral blood might reflect the degree of already established lung inflammation, it remains elusive. However, further studies are necessary to validate whether the amount of GPR15+ T cells may in fact indicate lung inflammation/destruction by smoking.

### Time course of methylation recovery

To exclude the possibility that the hypomethylation at cg0557921 in *AHRR* might be a long-lasting effect from mother’s smoking during pregnancy, we investigated the time course of methylation from birth until the age of 4 years in children from smoking compared to non-smoking mothers during pregnancy. In general, the cg0557921 was one of the most significant CpG in cord blood affected by tobacco smoking throughout pregnancy [16, 18, 20]. However, in accordance with studies showing that DNA methylation in blood of adult smokers after cessation approaches level of never smokers within few years, but never completely reaches normal levels [6, 8], we have shown that the different methylation level at cg0557921 in cord blood of newborns whose mothers had smoked during pregnancy disappeared until the age of 2 years. Because of the fast recovery during the first 2 years after birth, it seems unlikely that the hypomethylation at this CpG in adult smokers was caused by in utero exposure. In a further study including birth and ages of 7 and 17 years, a full recovery of methylation at cg0557921 in whole blood toward the level of those children not exposed to prenatal maternal smoke was not reached [21]. However, in this study, a maximum methylation was reported for the 7-year-old children followed by a slightly falling down to the age of 17 years [21].

The reason for the shift in methylation at cg0557921 over time in unexposed children remains elusive. But we think that changed methylation over the first year of life might be a sign of physiological maturation of immune system in general. Seemingly similar methylation changes were also found in *GFI1* and *CNTNAP2* showing an increase of methylation from birth to the age of 7 years [21]. Interestingly, all these three genes additionally exert their function as tumor suppressor genes (*AHRR* [28], *GFI1* [29], *CNTNAP2* [30]).

According to the time course of recovery of methylation after cessation in adults, the CpGs affected by tobacco smoking were classified by Guida and colleagues as persistent (cg19859270, *GPR15*; cg02657160, *CPOX*; cg0557921, *AHRR*; cg09935388, GFI1) or reversible (cg02319016, *PAK*; cg13086586, *PAICS*) [8]. This statement relied on genome-wide methylation data. However, taken into account that smoking-induced GPR15+ T cells are mainly central or effector memory T cells [31] with an even longer life span, we would expect that all conspicuous CpGs in this cell type could be interpreted as persistent, including the cg02319016 in *PAK.* The mechanistic interpretation of persistent CpGs mainly found in granulocytes, such as cg09935388 in *GFI*, remains unclear. In contrast to cells of the adaptive immune system, innate immune cells like granulocytes are expected to exhibit a comparable functional effector response each time the same pathogen is encountered [32]. Thus, activated immune cells might appear in peripheral blood as long as immune cell activating tobacco combustion products, which are deposited in the lung during active smoking, are not eliminated.

### Smoking-associated methylation changes in newborns

Comparing the most significant smoking-induced methylation changes in cord blood [16, 17, 19] with that of adult blood [6, 8], it is striking evident that in cord blood exclusively those CpG-annotated genes were highlighted which were dominantly regulated in granulocytes. With decreasing significance of CpGs the *AHRR*, *GFI1*, *MYO1G*, and *CYP1A1* were the top leading conspicuous genes in cord blood with altered methylation pattern following maternal smoking throughout pregnancy [16–18, 21]. According to our findings, the main methylation changes by smoking in CpGs of *AHRR* and *GFI1* were attributed to granulocytes. *MYO1G* was identified in whole blood samples of adult smokers [6, 8] but not in PBMC [4] indicating granulocytes as the main source of this methylation change at this CpG. The enzyme of biotransformation CYP1A1 is rather expressed in metabolic active granulocytes or monocytes than in lymphocytes [33, 34]. Additionally, it is of special interest that the smoking-induced hypomethylation at cg19859270 in GPR15+ T cells, representing the adaptive immunity, was never found in cord blood. As a potential reason for the missing GPR15 activation in cord blood, the presence of a specific population of suppressor T cells protecting the fetus from an adaptive immune reaction like graft-versus-host reaction in utero has been discussed [35]. Such suppressor cells were not found in adult blood.

### Missing in vitro effects of cigarette smoke extract

In a recent study, we have shown that CSE failed to induce the expansion of GPR15+ T cells in vitro [23]. Thus, we hypothesized that GPR15+ T cells, rather than being directly activated by tobacco smoke exposure, are stimulated by antigen-presenting cells in the lung, such as Langerhans cells, indicating a disturbed lung epithelial homeostasis by tobacco smoking. In the current study, we additionally have shown that CSE failed to induce hypomethylation at cg05575921 in *AHRR* both in isolated granulocytes and in PBMCs. This result again may give evidence for a more complex cellular mechanism leading to methylation changes in different cell types.

### Limitations

Analyzing exclusively top-ranked CpGs in whole blood or PBMC samples, we did not adjust for cellular composition. This seems to be a limitation of this study but, first, it was proven that top-ranked CpGs are not affected by composition of main blood cell types [16]. Second, identical smoking-induced top-ranked CpGs were repeatedly highlighted in different genome-wide association studies (GWAS) considering [3, 6] or not considering [1, 2] cell composition of blood. Third, we have shown that smoking-induced changes in methylation at top-ranked CpGs were present both in cell type-composed blood and separated blood cell types. Altogether, this indicates the plausibility of our findings not considering the cell composition of blood. To better understand the mechanism or causality of impaired methylation pattern in blood by smoking exposure, for instance, we clearly have shown the need to discover the cellular origin of each of the significant CpG. By this approach, we automatically would clarify if a change in methylation at given CpG relied on cell composition of the blood or not. On the other hand, if adjustment would have been performed for all known cell types of the blood, including the recently described smoking-induced GPR15+ T cells, then methylation changes at cg19859270 in *GPR15* would not have been detected in none of the GWAS. To overcome this limitation of incomplete adjustment for cell composition and to strengthen the focus on identification of biological cause of exposure-triggered methylation changes in blood, in general, we advise against adjustment of datasets obtained from whole blood for cell composition. An additional limitation was given by missing details about smoking behavior from blood donors; thus, we could not interpret the excess of GPR15+ cells among T cells to the daily frequency of smoking or cigarette pack-years. However, from the clinical site, for therapeutic purpose, more the individual qualitative and quantitative endpoints are of interest and less the tobacco smoke exposure leading to the phenotypic disturbance.

## Conclusions

The main intention of the present study was to clarify how to interpret the importance of epigenetic methylation changes at single CpGs in blood by tobacco smoking as one candidate of harmful exposure and whether these in general minor changes in DNA methylation in blood could be of biological relevance. Our data provide evidence that even minor methylation changes observed in whole blood samples may be of strong biological relevance if they can be attributed to a specific cell type. We could found two biomarkers for a disturbed homeostasis by tobacco smoking at subject level. Furthermore, we could postulate that the biological adaptation toward in utero tobacco smoke exposure is caused by cells of the innate immunity compared to active smoking in adulthood where cells of the innate and additionally of the adaptive immunity are important.

## Methods

### Subjects

For the present study, blood samples from three independent human blood donation cohorts and one prospective birth cohort were used. The three independent blood donation cohorts comprised blood samples from 148 volunteers (102 non-smokers and 46 smokers) obtained from the blood bank at the University of Leipzig (Table 2). Volunteers were recruited randomly in the years 2014/2015 (blood donation 2 cohort, *n* = 100) or as case-control cohorts after questionnaire regarding their smoking behavior (blood donation 1 [*n* = 25] and 3 [*n* = 23] cohorts). A standardized questionnaire collected data for age, gender, and smoking. There were no demographic differences among non-smokers or smokers between the three cohorts (Mann-Whitney *U* test, p > 0.05). Exposure to cigarette smoke was estimated via cotinine level measurement in whole blood. All volunteers were HIV-tested negative and gave their written consent. The study was approved by the Ethics Committee of the University of Leipzig (079-15-09032015).

**Table 2:** Description of study subjects

A) Adult cohorts (*n* = 3)					
			Gender		Cell type
			Male	Female	
Blood donation 1 (whole blood)					Granulocyte PBMC
Subject			17	8	
Age^a^			42 (24–66)	43 (30–65)	
Tobacco smoking					
No			5	6	
Yes		>5 cig/day	9	1	
		<10 cig/week	3	1	
Blood donation 2 (whole blood)					Whole blood
Subject			53	47	
Age^a^			44 (21–47)	46 (19–66)	
Tobacco smoking					
No			41	41	
Yes			12	6	
Blood donation 3 (buffy coat)					PBMC GPR15± T cells
Subject			14	9	
Age^a^			36 (24–64)	39 (21–71)	
Tobacco smoking					
No			6	3	
Yes			8	6	
B) Child cohort (LINA)					
Whole blood					Whole blood
			Smoking throughout pregnancy		
Age (year)			No	Yes	
0			20	13	
1			20	13	
2			20	11	
3			20	8	

*PBMC* peripheral blood mononuclear cells

^a^Median (min-max)

Blood samples of children and their corresponding mothers were used from the prospective birth cohort study LINA. Prenatal tobacco smoke-exposed children were defined due to maternal urine cotinine levels (>100 μg/g creatinine at the 34th week of pregnancy; controls, <1 μg/g creatinine). The same children and their mothers were investigated at consecutive time periods (see Additional file 7: Table S4). Participation in the study was voluntary, and informed consent was obtained from all participants. The study was approved by the Ethics Committee of the University of Leipzig (046-2006, 160-2008).

### Leukocyte separation

PBMCs were obtained by density-gradient centrifugation using Ficoll-Paque (GE Healthcare, Solingen, Germany). Granulocytes from fresh blood were obtained by density-gradient centrifugation using Mono-Poly resolving medium (MPRM, MP Biomedical, Heidelberg, Germany) within 2 h after donation. To enhance the purity of granulocytes, MPRM was overlayed with Ficoll-Paque. GPR15+CD3+ and GPR15−CD3+ T cells were separated by flow-cytometric cell sorting after antibody staining of PBMCs for CD3 and GPR15 surface proteins as indicated below. The rationale of the implementation of this cell type was given by our recent finding that a tobacco smoking-induced hypomethylation at cg19859270 in blood was caused by the excess of GPR15+ T cells harboring this cell type-specific CpG. Thus, we were intended to prove if additional CpG methylation shifts by smoking might rely on the excess of this cell type. Flow-cytometric cell sorting was performed at the laboratory of cytometry of the Core Facility at the University of Leipzig.

### Flow-cytometric analysis

All antibody incubation and wash steps were performed in PBS/1%FCS at room temperature. First, the PBMCs were stained for GPR15 and thereafter for the CD3 surface receptor. Briefly, cells were incubated with anti-GPR15 antibody (1:500; R&D Systems, Wiesbaden-Nordenstadt, Germany) supplemented with 5 % goat serum for 1 hour and stained with R-phycoerythrin-labeled anti-mouse IgG2b (1:500; Biozol, Eching, Germany). Free IgG2b binding sites were blocked thereafter with 5 % mouse serum for 30 min followed by staining for leukocyte differentiation receptor (anti-CD3-FITC [1:200, Beckman Coulter, Krefeld, Germany]). The stained cells were examined for fluorescence with the FACS Canto II and analyzed with BD FACSDIVA software (version 8.0.1, BD Biosciences, Heidelberg, Germany). When analyzing leukocytes in whole blood, the first incubation step was performed directly in 100 μl of blood samples. Erythrocytes were lysed in FACS Lysing solution (BD Bioscience, Heidelberg, Germany) according to the manufacturer’s instruction immediately before fluorescence measurement.

### Cotinine ELISA

The cotinine concentration in plasma of blood or blood buffy coat samples was measured using the Cotinine direct ELISA Kit according to the manufacturer’s instruction (DRG Instruments GmbH, Marburg, Germany). A cotinine level exceeding the sensitivity level of the assay (1 ng/ml) was considered as smoker.

### Pyrosequencing

Genomic DNA was extracted from leukocytes by using the Blood DNA extraction kit according to the manufacturer’s protocol (Qiagen, Hilden, Germany). DNA bisulfite treatment was performed using the Epitect kit (Qiagen) according to the manufacturer’s instruction. Samples were immediately stored at −20 °C and thereafter simultaneously analyzed by pyrosequencing. Methylation assays were designed using the PyroMark Assay Design Software 2.0 (www.qiagen.com). Primer sequences for pyrosequencing are indicated (see Additional file 8: Table S1). Methylation levels for the CpG site were assessed using Pyromark Q24 pyrosequencer (Qiagen). Selection criteria for single CpG were the significance of replicated in different reports single CpGs as well as their chromosomal location nearby a gene.

### RNA expression

For semi-quantitative PCR (qPCR), total RNA of separated leukocytes was prepared by using peqGold RNA Pure (peqlab, Erlangen, Germany) according to the manufacturer’s instructions. The cDNA synthesis was carried out with 1 μg of RNA by using ImProm-II™ Reverse Transcription System (Promega, Mannheim, Germany). Intron-spanning primers were designed, and UPL probes were selected by the Universal Probe Library Assay Design Center (http://qpcr.probefinder.com/organism.jsp; AHRR-for 5′- tgcttcatctgccgtgtg, -rev 5′- agctgccaagcctgtgac, UPL 56; GUSB-for 5′- cgccctgcctatctgtattc, -rev 5′- tccccacagggagtgtgtag, UPL 57). The cycling program consisted of 95 °C for 5 min, followed by 45 cycles of 95 °C for 15 s and 60 °C for 1 min and 72 °C for 15 s on a LightCycler 480 (Roche Applied Science, Mannheim, Germany). PCR was performed with FastStart Universal Probe Master Mix (Roche, Mannheim, Germany). All reactions were performed in duplicates. Gene expression values were determined by using the 2−∆∆CT method [36] with GUSB as reference gene and normalized to the lowest measured value.

### In vitro exposure of leukocytes

Separated granulocytes and PBMC were immediately cultured in 96-well round-bottom plates in RPMI 1640 medium supplemented with 10 % FCS, glutamine, and 25 mM HEPES (Life Technologies, Darmstadt, Germany) without antibiotics. To prolong granulocytes survival in vitro, they were additionally stimulated with 200 U/ml IL1-β [37]. Cells were exposed to CSE in concentrations of 1:1000, 1:100, and 1:10 for 2 days. For procedure of preparation of aqueous CSE (see Bauer et al. 2015 [23]). Cellular toxicity was assessed by trypan blue exclusion.

### Statistical analysis

Smoking-dependent differences in methylation at single CpG in different cell populations were tested using the unpaired Student’s *t* test (in case of normal distributed data) or Mann-Whitney *U* test, respectively. Test of normality was performed using Shapiro-Wilks test. Since we focused here only on a selected set of CpGs, frequently reported in earlier studies as differentially methylated according to tobacco smoke exposure, statistical correction for multiple testing was not indicated. The non-parametric Spearman correlation was used to describe relationship between the two highlighted biomarkers and between methylation levels of mothers and their children at single CpG. Multiple comparison of cg05575921 methylation over the first 4 years of children’s life was assessed by Kruskal-Wallis ANOVA by ranks (*p* < 0.016 was considered to be significant). Age-dependent differences in methylation at cg05575921 between tobacco smoke-exposed and non-exposed children during pregnancy and their mothers were calculated by Student’s *t* test or Mann-Whitney *U* test. Boxes in figures indicate the 25 and 75 % percentile, whiskers the non-outlier range, and dots the outlier. All *p* values <0.05 were considered to be significant. All statistical calculations were performed with Statistica for Windows version 10 (StatSoft Inc. Europe).

Statistical values from three implemented epidemiological studies are indicated as have been published by the authors.

## Abbreviations

CpG, cytosine nucleotide-phosphate-guanine nucleotide; CSE, cigarette smoke extract; PBMC, peripheral blood mononuclear cells

## Additional files

Additional file 1: Original data of three published reports which were used for building the Venn diagram of Fig. 1. (DOC 2580 kb) Additional file 2: Estimation of the prominent cell type of whole blood accounting for major smoking-associated methylation change (∆meth) at single CpG site based on reports of methylation changes in whole blood and peripheral blood mononuclear cells (PBMC). (DOCX 20 kb) Additional file 3: Distribution of methylation changes at single CpGs by tobacco smoking in different cell populations of whole blood. Highlighted are significant methylation changes. NS, non-smoker; S, smoker; *p*, *P*, statistical significance by Student’s *t* test (*p*) or Mann-Whitney *U* test (*P*). (PDF 81 kb) Additional file 4: Spearman correlation between amount of GPR15+ T cells and methylation at cg05575921 in granulocytes in non-smokers and smokers. Both biomarkers did not correlate significantly with each other. (PDF 101 kb) Additional file 5: Figure S2. Influence of cigarette smoke extract (CSE) exposure on in vitro cultured PBMC and granulocytes (48 h) of non-smokers (*n* = 3, each) on methylation at cg05575921 (*AHRR*). CSE did not change the methylation at cg05575921. (PDF 27 kb) Additional file 6: Venn diagram illustrating intersections of tobacco smoking-evoked methylation changes at single CpGs of four different reports using genomic DNA isolated from whole blood (WBC), from separated peripheral blood mononuclear cells (PBMC) of blood or from cells collected from the inside of a person’s cheek (buccal cells) of adult probands. Origin of data sets is indicated. (DOCX 20 kb) Additional file 7: Sample sizes of the prospective birth cohort study LINA. (DOCX 16 kb) Additional file 8: Primer for pyrosequencing. (DOCX 15 kb)

## References

[1] Breitling LP, Yang R, Korn B, Burwinkel B, Brenner H (2011). Tobacco-smoking-related differential DNA methylation: 27K discovery and replication. Am J Hum Genet.

[2] Wan ES, Qiu W, Baccarelli A, Carey VJ, Bacherman H, Rennard SI (2012). Cigarette smoking behaviors and time since quitting are associated with differential DNA methylation across the human genome. Hum Mol Genet.

[3] Sun YV, Smith AK, Conneely KN, Chang Q, Li W, Lazarus A (2013). Epigenomic association analysis identifies smoking-related DNA methylation sites in African Americans. Hum Genet.

[4] Dogan MV, Shields B, Cutrona C, Gao L, Gibbons FX, Simons R (2014). The effect of smoking on DNA methylation of peripheral blood mononuclear cells from African American women. BMC Genomics.

[5] Tsaprouni LG, Yang TP, Bell J, Dick KJ, Kanoni S, Nisbet J (2014). Cigarette smoking reduces DNA methylation levels at multiple genomic loci but the effect is partially reversible upon cessation. Epigenetics.

[6] Zeilinger S, Kuhnel B, Klopp N, Baurecht H, Kleinschmidt A, Gieger C (2013). Tobacco smoking leads to extensive genome-wide changes in DNA methylation. PLoS One.

[7] Shenker NS, Polidoro S, van Veldhoven K, Sacerdote C, Ricceri F, Birrell MA (2013). Epigenome-wide association study in the European Prospective Investigation into Cancer and Nutrition (EPIC-Turin) identifies novel genetic loci associated with smoking. Hum Mol Genet.

[8] Guida F, Sandanger TM, Castagne R, Campanella G, Polidoro S, Palli D (2015). Dynamics of smoking-induced genome-wide methylation changes with time since smoking cessation. Hum Mol Genet.

[9] Harlid S, Xu Z, Panduri V, Sandler DP, Taylor JA (2014). CpG sites associated with cigarette smoking: analysis of epigenome-wide data from the Sister Study. Environ Health Perspect.

[10] Monick MM, Beach SR, Plume J, Sears R, Gerrard M, Brody GH (2012). Coordinated changes in AHRR methylation in lymphoblasts and pulmonary macrophages from smokers. Am J Med Genet B Neuropsychiatr Genet.

[11] Philibert RA, Beach SR, Brody GH (2012). Demethylation of the aryl hydrocarbon receptor repressor as a biomarker for nascent smokers. Epigenetics.

[12] Ambatipudi S, Cuenin C, Hernandez-Vargas H, Ghantous A, Le Calvez-Kelm F, Kaaks R (2016). Tobacco smoking-associated genome-wide DNA methylation changes in the EPIC study. Epigenomics.

[13] Breton CV, Byun HM, Wenten M, Pan F, Yang A, Gilliland FD (2009). Prenatal tobacco smoke exposure affects global and gene-specific DNA methylation. Am J Respir Crit Care Med.

[14] Ivorra C, Fraga MF, Bayon GF, Fernandez AF, Garcia-Vicent C, Chaves FJ (2015). DNA methylation patterns in newborns exposed to tobacco in utero. J Transl Med.

[15] Joubert BR, Haberg SE, Bell DA, Nilsen RM, Vollset SE, Midttun O (2014). Maternal smoking and DNA methylation in newborns: in utero effect or epigenetic inheritance?. Cancer Epidemiol Biomarkers Prev.

[16] Joubert BR, Haberg SE, Nilsen RM, Wang X, Vollset SE, Murphy SK (2012). 450K epigenome-wide scan identifies differential DNA methylation in newborns related to maternal smoking during pregnancy. Environ Health Perspect.

[17] Kupers LK, Xu X, Jankipersadsing SA, Vaez A, la Bastide-van Gemert S, Scholtens S (2015). DNA methylation mediates the effect of maternal smoking during pregnancy on birthweight of the offspring. Int J Epidemiol.

[18] Lee KW, Richmond R, Hu P, French L, Shin J, Bourdon C (2015). Prenatal exposure to maternal cigarette smoking and DNA methylation: epigenome-wide association in a discovery sample of adolescents and replication in an independent cohort at birth through 17 years of age. Environ Health Perspect.

[19] Markunas CA, Xu Z, Harlid S, Wade PA, Lie RT, Taylor JA (2014). Identification of DNA methylation changes in newborns related to maternal smoking during pregnancy. Environ Health Perspect.

[20] Novakovic B, Ryan J, Pereira N, Boughton B, Craig JM, Saffery R (2014). Postnatal stability, tissue, and time specific effects of AHRR methylation change in response to maternal smoking in pregnancy. Epigenetics.

[21] Richmond RC, Simpkin AJ, Woodward G, Gaunt TR, Lyttleton O, McArdle WL (2015). Prenatal exposure to maternal smoking and offspring DNA methylation across the lifecourse: findings from the Avon Longitudinal Study of Parents and Children (ALSPAC). Hum Mol Genet.

[22] Bauer T, Trump S, Ishaque N, Thurmann L, Gu L, Bauer M (2016). Environment-induced epigenetic reprogramming in genomic regulatory elements in smoking mothers and their children. Mol Syst Biol.

[23] Bauer M, Linsel G, Fink B, Offenberg K, Hahn AM, Sack U (2015). A varying T cell subtype explains apparent tobacco smoking induced single CpG hypomethylation in whole blood. Clin Epigenetics.

[24] Koks G, Uudelepp ML, Limbach M, Peterson P, Reimann E, Koks S (2015). Smoking-induced expression of the GPR15 gene indicates its potential role in chronic inflammatory pathologies. Am J Pathol.

[25] Teschendorff AE, Yang Z, Wong A, Pipinikas CP, Jiao Y, Jones A (2015). Correlation of smoking-associated DNA methylation changes in buccal cells with DNA methylation changes in epithelial cancer. JAMA Oncol.

[26] Gao X, Jia M, Zhang Y, Breitling LP, Brenner H (2015). DNA methylation changes of whole blood cells in response to active smoking exposure in adults: a systematic review of DNA methylation studies. Clin Epigenetics.

[27] Dauber EM, Gorner G, Mitterbauer M, Wenda S, Fae I, Glock B et al. Discrepant results of samples taken from different tissues of a single individual. Int Congr Ser. 2004;(1261):48-9.

[28] Zudaire E, Cuesta N, Murty V, Woodson K, Adams L, Gonzalez N (2008). The aryl hydrocarbon receptor repressor is a putative tumor suppressor gene in multiple human cancers. J Clin Invest.

[29] Hones JM, Botezatu L, Helness A, Vadnais C, Vassen L, Robert F (2016). GFI1 as a novel prognostic and therapeutic factor for AML/MDS. Leukemia.

[30] Bralten LB, Gravendeel AM, Kloosterhof NK, Sacchetti A, Vrijenhoek T, Veltman JA (2010). The CASPR2 cell adhesion molecule functions as a tumor suppressor gene in glioma. Oncogene.

[31] Kiene M, Rethi B, Jansson M, Dillon S, Lee E, Lantto R (2014). Toll-like receptor 3 signalling up-regulates expression of the HIV co-receptor G-protein coupled receptor 15 on human CD4+ T cells. PLoS One.

[32] Narni-Mancinelli E, Soudja SM, Crozat K, Dalod M, Gounon P, Geissmann F (2011). Inflammatory monocytes and neutrophils are licensed to kill during memory responses in vivo. PLoS Pathog.

[33] Bahari A, Mehrzad J, Mahmoudi M, Bassami MR, Dehghani H (2014). Cytochrome P450 isoforms are differently up-regulated in aflatoxin B(1)-exposed human lymphocytes and monocytes. Immunopharmacol Immunotoxicol.

[34] Butkiewicz D, Grzybowska E, Hemminki K, Ovrebo S, Haugen A, Motykiewicz G (1998). Modulation of DNA adduct levels in human mononuclear white blood cells and granulocytes by CYP1A1 CYP2D6 and GSTM1 genetic polymorphisms. Mutat Res.

[35] Tosato G, Magrath IT, Koski IR, Dooley NJ, Blaese RM (1980). B cell differentiation and immunoregulatory T cell function in human cord blood lymphocytes. J Clin Invest.

[36] Livak KJ, Schmittgen TD (2001). Analysis of relative gene expression data using real-time quantitative PCR and the 2(-Delta Delta C(T)) Method. Methods.

[37] Colotta F, Re F, Polentarutti N, Sozzani S, Mantovani A (1992). Modulation of granulocyte survival and programmed cell death by cytokines and bacterial products. Blood.

