# Supplementary material for: Tobacco smoking differently influences cell types of the innate and adaptive immune system—indications from CpG site methylation
Source: Clin Epigenetics. 2016 Aug 3;8:83. doi: 10.1186/s13148-016-0249-7 (PMC4973040; doi:10.1186/s13148-016-0249-7)
Supplement: Additional file 1: — Original data of three published reports which were used for building the Venn diagram of Fig. 1. (DOC 2580 kb) [file 13148_2016_249_MOESM1_ESM.doc]

|  |  |  |  |  |  |  |  |  |  |  |  |  |  |  |  |  |  |  |  |  |
| --- | --- | --- | --- | --- | --- | --- | --- | --- | --- | --- | --- | --- | --- | --- | --- | --- | --- | --- | --- | --- |
| **CpG** | Zeilinger et al. [PMID: 23691101] | | | Guida et al. [PMID: 25556184] | | | Dogan et al. [PMID: 24559495] | | |  | **Estimation of intersectional CpGs** | | |  |  |  |  |  |  |  |
|  |  |  |  |  |  |  |  |  |  |  |  |  |  |  |  |  |  |  |  |  |
|  | **CpG** | Gene | p-value | **CpG** | Gene | p-value | **CpG** | Gene | p-value |  | Zeilinger et al. | Guida et al. | Both | Zeilinger et al. | Dogan et al. | Both | Guida et al. | Dogan et al. | Both | Triple |
|  |  |  | [Bonferroni correction for multiple testing. p<1.06E-07] |  |  | [Bonferroni correction for multiple testing, p<1.15E-07] |  |  | [Benjamini-Hochberg correction for multiple testing] |  |  |  |  |  |  |  |  |  |  |  |
|  |  |  |  |  |  |  |  |  |  |  | **72** | **345** | **116** | **163** | **885** | **25** | **430** | **879** | **31** | **24** |
| cg01899089 | cg01899089 | AHRR | 8.95E-18 | cg01899089 | AHRR | 3.58E-16 | cg01899089 | AHRR | 2.05E-06 |  |  |  | 1 |  |  | 1 |  |  | 1 | 1 |
| cg03991871 | cg03991871 | AHRR | 4.84E-28 | cg03991871 | AHRR | 1.63E-21 | cg03991871 | AHRR | 4.01E-07 |  |  |  | 1 |  |  | 1 |  |  | 1 | 1 |
| cg05575921 | cg05575921 | AHRR | 2.54E-168 | cg05575921 | AHRR | 3.05E-106 | cg05575921 | AHRR | 6.17E-19 |  |  |  | 1 |  |  | 1 |  |  | 1 | 1 |
| cg12806681 | cg12806681 | AHRR | 3.63E-19 | cg12806681 | AHRR | 4.36E-13 | cg12806681 | AHRR | 5.42E-05 |  |  |  | 1 |  |  | 1 |  |  | 1 | 1 |
| cg21161138 | cg21161138 | AHRR | 8.58E-67 | cg21161138 | AHRR | 8.51E-57 | cg21161138 | AHRR | 4.64E-12 |  |  |  | 1 |  |  | 1 |  |  | 1 | 1 |
| cg23916896 | cg23916896 | AHRR | 9.79E-15 | cg23916896 | AHRR | 2.93E-19 | cg23916896 | AHRR | 5.11E-06 |  |  |  | 1 |  |  | 1 |  |  | 1 | 1 |
| cg25648203 | cg25648203 | AHRR | 4.73E-33 | cg25648203 | AHRR | 5.14E-37 | cg25648203 | AHRR | 3.66E-06 |  |  |  | 1 |  |  | 1 |  |  | 1 | 1 |
| cg26703534 | cg26703534 | AHRR | 2.14E-24 | cg26703534 | AHRR | 9.82E-59 | cg26703534 | AHRR | 2.52E-08 |  |  |  | 1 |  |  | 1 |  |  | 1 | 1 |
| cg01940273 | cg01940273 | ALPPL2b | 9.28E-100 | cg01940273 | NA | 7.44E-74 | cg01940273 |  | 2.51E-10 |  |  |  | 1 |  |  | 1 |  |  | 1 | 1 |
| cg03329539 | cg03329539 | ALPPL2b | 3.66E-45 | cg03329539 | NA | 6.51E-35 | cg03329539 |  | 5.21E-05 |  |  |  | 1 |  |  | 1 |  |  | 1 | 1 |
| cg05951221 | cg05951221 | ALPPL2bALPPL2bALPPL2bALPPL2bALPPL2bALPPL2bALPPL2bALPPL2bALPPL2bALPPL2bALPPL2bALPPL2bALPPL2bALPPL2bALPPL2bALPPL2bALPPL2bALPPL2bALPPL2bALPPL2bALPPL2bALPPL2bALPPL2bALPPL2bALPPL2bALPPL2bALPPL2bALPPL2bALPPL2bALPPL2bALPPL2bALPPL2bALPPL2bALPPL2bALPPL2bALPPL2bALPPL2bALPPL2bALPPL2bALPPL2bALPPL2bALPPL2bALPPL2bALPPL2bALPPL2bALPPL2bALPPL2bALPPL2bALPPL2bALPPL2bALPPL2bALPPL2bALPPL2bALPPL2bALPPL2bALPPL2bALPPL2bALPPL2bALPPL2bALPPL2bALPPL2bALPPL2bALPPL2bALPPL2bALPPL2bALPPL2bALPPL2bALPPL2bALPPL2bALPPL2bALPPL2bALPPL2bALPPL2bALPPL2bALPPL2bALPPL2bALPPL2bALPPL2bALPPL2bALPPL2bALPPL2bALPPL2bALPPL2bALPPL2bALPPL2bALPPL2bALPPL2bALPPL2b | 8.92E-90 | cg05951221 | NA | 7.52E-69 | cg05951221 |  | 4.23E-08 |  |  |  | 1 |  |  | 1 |  |  | 1 | 1 |
| cg21566642 | cg21566642 | ALPPL2bALPPL2bALPPL2bALPPL2bALPPL2bALPPL2bALPPL2bALPPL2bALPPL2bALPPL2bALPPL2bALPPL2bALPPL2bALPPL2bALPPL2bALPPL2bALPPL2bALPPL2bALPPL2bALPPL2bALPPL2bALPPL2bALPPL2bALPPL2bALPPL2bALPPL2bALPPL2bALPPL2bALPPL2bALPPL2bALPPL2bALPPL2bALPPL2bALPPL2bALPPL2bALPPL2bALPPL2bALPPL2bALPPL2bALPPL2bALPPL2bALPPL2bALPPL2bALPPL2bALPPL2bALPPL2bALPPL2bALPPL2bALPPL2bALPPL2bALPPL2bALPPL2bALPPL2bALPPL2bALPPL2bALPPL2bALPPL2bALPPL2bALPPL2bALPPL2bALPPL2bALPPL2bALPPL2bALPPL2bALPPL2bALPPL2bALPPL2bALPPL2bALPPL2bALPPL2bALPPL2bALPPL2bALPPL2bALPPL2bALPPL2bALPPL2bALPPL2bALPPL2bALPPL2bALPPL2bALPPL2bALPPL2bALPPL2bALPPL2bALPPL2bALPPL2bALPPL2bALPPL2b | 6.90E-124 | cg21566642 | NA | 2.14E-79 | cg21566642 |  | 2.99E-10 |  |  |  | 1 |  |  | 1 |  |  | 1 | 1 |
| cg01731783 | cg01731783 | C14orf43 | 3.48E+03 | cg01731783 | C14orf43 | 4.27E-12 | cg01731783 | C14orf43 | 2.65E-05 |  |  |  | 1 |  |  | 1 |  |  | 1 | 1 |
| cg22851561 | cg22851561 | C14orf43 | 5.47E-07 | cg22851561 | C14orf43 | 5.81E-12 | cg22851561 | C14orf43 | 2.40E-07 |  |  |  | 1 |  |  | 1 |  |  | 1 | 1 |
| cg02657160 | cg02657160 | CPOX | 1.67E+05 | cg02657160 | CPOX | 5.55E-15 | cg02657160 | CPOX | 3.97E-12 |  |  |  | 1 |  |  | 1 |  |  | 1 | 1 |
| cg03636183 | cg03636183 | F2RL3 | 2.42E-66 | cg03636183 | F2RL3 | 5.37E-82 | cg03636183 | F2RL3 | 2.30E-07 |  |  |  | 1 |  |  | 1 |  |  | 1 | 1 |
| cg09935388 | cg09935388 | GFI1 | 3.27E-10 | cg09935388 | GFI1 | 9.56E-33 | cg09935388 | GFI1 | 3.31E-08 |  |  |  | 1 |  |  | 1 |  |  | 1 | 1 |
| cg12876356 | cg12876356 | GFI1 | 2.92E-03 | cg12876356 | GFI1 | 1.63E-17 | cg12876356 | GFI1 | 4.47E-05 |  |  |  | 1 |  |  | 1 |  |  | 1 | 1 |
| cg19859270 | cg19859270 | GPR15 | 9.00E-11 | cg19859270 | GPR15 | 8.57E-27 | cg19859270 | GPR15 | 1.19E-19 |  |  |  | 1 |  |  | 1 |  |  | 1 | 1 |
| cg12075928 | cg12075928 | PTK2 | 1.40E-04 | cg12075928 | PTK2 | 9.70E-12 | cg12075928 | PTK2 | 2.46E-05 |  |  |  | 1 |  |  | 1 |  |  | 1 | 1 |
| cg19572487 | cg19572487 | RARA | 9.37E-26 | cg19572487 | RARA | 4.82E-29 | cg19572487 | RARA | 1.02E-05 |  |  |  | 1 |  |  | 1 |  |  | 1 | 1 |
| cg04885881 | cg04885881 | xaxaxaxaxaxaxaxaxaxaxaxaxaxaxaxaxaxaxaxaxaxaxaxaxaxaxaxaxaxaxaxaxaxaxaxaxaxaxaxaxaxaxaxaxaxaxaxaxaxaxaxaxaxaxaxaxaxaxaxaxaxaxaxaxaxaxaxaxaxaxaxaxaxaxaxaxaxaxaxaxaxaxaxaxaxaxaxa | 1.35E-14 | cg04885881 | NA | 1.41E-25 | cg04885881 |  | 5.46E-06 |  |  |  | 1 |  |  | 1 |  |  | 1 | 1 |
| cg06126421 | cg06126421 | xaxaxaxaxaxaxaxaxaxaxaxaxaxaxaxaxaxaxaxaxaxaxaxaxaxaxaxaxaxaxaxaxaxaxaxaxaxaxaxaxaxaxaxaxaxaxaxaxaxaxaxaxaxaxaxaxaxaxaxaxaxaxaxaxaxaxaxaxaxaxaxaxaxaxaxaxaxaxaxaxaxaxaxaxaxaxaxa | 1.72E-61 | cg06126421 | NA | 2.00E-60 | cg06126421 |  | 2.91E-10 |  |  |  | 1 |  |  | 1 |  |  | 1 | 1 |
| cg24859433 | cg24859433 | xaxaxaxaxaxaxaxaxaxaxaxaxaxaxaxaxaxaxaxaxaxaxaxaxaxaxaxaxaxaxaxaxaxaxaxaxaxaxaxaxaxaxaxaxaxaxaxaxaxaxaxaxaxaxaxaxaxaxaxaxaxaxaxaxaxaxaxaxaxaxaxaxaxaxaxaxaxaxaxaxaxaxaxaxaxaxaxa | 3.06E-27 | cg24859433 | NA | 4.43E-35 | cg24859433 |  | 1.16E-05 |  |  |  | 1 |  |  | 1 |  |  | 1 | 1 |
| cg08672695 |  |  |  |  |  |  | cg08672695 |  | 7.40E-15 |  |  |  |  |  | 1 |  |  | 1 |  |  |
| cg18230367 |  |  |  |  |  |  | cg18230367 | RNASE4 | 1.48E-11 |  |  |  |  |  | 1 |  |  | 1 |  |  |
| cg02319016 |  |  |  |  |  |  | cg02319016 | PAK2 | 9.27E-11 |  |  |  |  |  | 1 |  |  | 1 |  |  |
| cg26607002 |  |  |  |  |  |  | cg26607002 | NOSTRIN | 2.32E-10 |  |  |  |  |  | 1 |  |  | 1 |  |  |
| cg04677326 |  |  |  |  |  |  | cg04677326 | C19orf28 | 2.42E-10 |  |  |  |  |  | 1 |  |  | 1 |  |  |
| cg05457881 |  |  |  |  |  |  | cg05457881 | C6orf218 | 2.79E-10 |  |  |  |  |  | 1 |  |  | 1 |  |  |
| cg13086586 |  |  |  |  |  |  | cg13086586 | PAICS | 1.05E-09 |  |  |  |  |  | 1 |  |  | 1 |  |  |
| cg15281724 |  |  |  |  |  |  | cg15281724 | TXLNB | 2.92E-09 |  |  |  |  |  | 1 |  |  | 1 |  |  |
| cg15645254 |  |  |  |  |  |  | cg15645254 | NAALAD2 | 3.51E-09 |  |  |  |  |  | 1 |  |  | 1 |  |  |
| cg19111030 |  |  |  |  |  |  | cg19111030 | ANKRD53 | 5.30E-09 |  |  |  |  |  | 1 |  |  | 1 |  |  |
| cg09741592 |  |  |  |  |  |  | cg09741592 | HNRNPA1 | 8.25E-09 |  |  |  |  |  | 1 |  |  | 1 |  |  |
| cg08528204 |  |  |  |  |  |  | cg08528204 | TMEM116 | 2.07E-08 |  |  |  |  |  | 1 |  |  | 1 |  |  |
| cg17391741 |  |  |  |  |  |  | cg17391741 |  | 2.07E-08 |  |  |  |  |  | 1 |  |  | 1 |  |  |
| cg15614155 |  |  |  |  |  |  | cg15614155 |  | 2.34E-08 |  |  |  |  |  | 1 |  |  | 1 |  |  |
| cg05916255 |  |  |  |  |  |  | cg05916255 | ABCC2 | 2.47E-08 |  |  |  |  |  | 1 |  |  | 1 |  |  |
| cg00736283 |  |  |  |  |  |  | cg00736283 | ASF1B | 2.52E-08 |  |  |  |  |  | 1 |  |  | 1 |  |  |
| cg25223391 |  |  |  |  |  |  | cg25223391 | UVRAG | 2.52E-08 |  |  |  |  |  | 1 |  |  | 1 |  |  |
| cg16851858 |  |  |  |  |  |  | cg16851858 |  | 2.80E-08 |  |  |  |  |  | 1 |  |  | 1 |  |  |
| cg02521854 |  |  |  |  |  |  | cg02521854 |  | 2.93E-08 |  |  |  |  |  | 1 |  |  | 1 |  |  |
| cg15658543 |  |  |  |  |  |  | cg15658543 | CARD11 | 3.20E-08 |  |  |  |  |  | 1 |  |  | 1 |  |  |
| cg13789443 |  |  |  |  |  |  | cg13789443 | GALNT11 | 3.24E-08 |  |  |  |  |  | 1 |  |  | 1 |  |  |
| cg26381918 |  |  |  |  |  |  | cg26381918 | C2orf64 | 3.26E-08 |  |  |  |  |  | 1 |  |  | 1 |  |  |
| cg25882591 |  |  |  |  |  |  | cg25882591 |  | 4.86E-08 |  |  |  |  |  | 1 |  |  | 1 |  |  |
| cg18332146 |  |  |  |  |  |  | cg18332146 | CTSC | 6.71E-08 |  |  |  |  |  | 1 |  |  | 1 |  |  |
| cg09174741 |  |  |  |  |  |  | cg09174741 | THSD1 | 8.55E-08 |  |  |  |  |  | 1 |  |  | 1 |  |  |
| cg15105252 |  |  |  |  |  |  | cg15105252 |  | 1.01E-07 |  |  |  |  |  | 1 |  |  | 1 |  |  |
| cg16105594 |  |  |  |  |  |  | cg16105594 | MEF2C | 1.07E-07 |  |  |  |  |  | 1 |  |  | 1 |  |  |
| cg18095109 |  |  |  |  |  |  | cg18095109 |  | 1.07E-07 |  |  |  |  |  | 1 |  |  | 1 |  |  |
| cg05944967 |  |  |  |  |  |  | cg05944967 | NFATC1 | 1.10E-07 |  |  |  |  |  | 1 |  |  | 1 |  |  |
| cg25677394 |  |  |  |  |  |  | cg25677394 | HRH1 | 1.23E-07 |  |  |  |  |  | 1 |  |  | 1 |  |  |
| cg16830479 |  |  |  |  |  |  | cg16830479 | ZC3H12B | 1.23E-07 |  |  |  |  |  | 1 |  |  | 1 |  |  |
| cg18327772 |  |  |  |  |  |  | cg18327772 | HMGB4 | 1.29E-07 |  |  |  |  |  | 1 |  |  | 1 |  |  |
| cg03333116 |  |  |  |  |  |  | cg03333116 | RHBDF1 | 1.43E-07 |  |  |  |  |  | 1 |  |  | 1 |  |  |
| cg10369313 |  |  |  |  |  |  | cg10369313 |  | 1.71E-07 |  |  |  |  |  | 1 |  |  | 1 |  |  |
| cg26309498 |  |  |  |  |  |  | cg26309498 | EDAR | 1.78E-07 |  |  |  |  |  | 1 |  |  | 1 |  |  |
| cg00389785 |  |  |  |  |  |  | cg00389785 |  | 2.00E-07 |  |  |  |  |  | 1 |  |  | 1 |  |  |
| cg09773647 |  |  |  |  |  |  | cg09773647 |  | 2.03E-07 |  |  |  |  |  | 1 |  |  | 1 |  |  |
| cg05157912 |  |  |  |  |  |  | cg05157912 | JDP2 | 2.24E-07 |  |  |  |  |  | 1 |  |  | 1 |  |  |
| cg11553667 |  |  |  |  |  |  | cg11553667 |  | 2.25E-07 |  |  |  |  |  | 1 |  |  | 1 |  |  |
| cg04120407 |  |  |  |  |  |  | cg04120407 | KLHDC4 | 2.30E-07 |  |  |  |  |  | 1 |  |  | 1 |  |  |
| cg18450625 |  |  |  |  |  |  | cg18450625 | EPB42 | 2.41E-07 |  |  |  |  |  | 1 |  |  | 1 |  |  |
| cg12636538 |  |  |  |  |  |  | cg12636538 | SLC37A4 | 2.41E-07 |  |  |  |  |  | 1 |  |  | 1 |  |  |
| cg14254999 |  |  |  |  |  |  | cg14254999 | GPR55 | 3.39E-07 |  |  |  |  |  | 1 |  |  | 1 |  |  |
| cg24078767 |  |  |  |  |  |  | cg24078767 | IFI35 | 3.39E-07 |  |  |  |  |  | 1 |  |  | 1 |  |  |
| cg19477192 |  |  |  |  |  |  | cg19477192 | CCDC42B | 3.51E-07 |  |  |  |  |  | 1 |  |  | 1 |  |  |
| cg17417347 |  |  |  |  |  |  | cg17417347 | GLYATL3 | 3.51E-07 |  |  |  |  |  | 1 |  |  | 1 |  |  |
| cg16906995 |  |  |  |  |  |  | cg16906995 |  | 3.72E-07 |  |  |  |  |  | 1 |  |  | 1 |  |  |
| cg05062814 |  |  |  |  |  |  | cg05062814 | C6orf64 | 3.87E-07 |  |  |  |  |  | 1 |  |  | 1 |  |  |
| cg02995567 |  |  |  |  |  |  | cg02995567 | TUBB2A | 3.87E-07 |  |  |  |  |  | 1 |  |  | 1 |  |  |
| cg24671951 |  |  |  |  |  |  | cg24671951 | ATP8B2 | 3.90E-07 |  |  |  |  |  | 1 |  |  | 1 |  |  |
| cg04689145 |  |  |  |  |  |  | cg04689145 | FRMD4B | 4.00E-07 |  |  |  |  |  | 1 |  |  | 1 |  |  |
| cg17414733 |  |  |  |  |  |  | cg17414733 | ZNF429 | 4.00E-07 |  |  |  |  |  | 1 |  |  | 1 |  |  |
| cg26687746 |  |  |  |  |  |  | cg26687746 |  | 4.00E-07 |  |  |  |  |  | 1 |  |  | 1 |  |  |
| cg08369295 |  |  |  |  |  |  | cg08369295 | LRP5L | 4.01E-07 |  |  |  |  |  | 1 |  |  | 1 |  |  |
| cg13397649 |  |  |  |  |  |  | cg13397649 | AFG3L2 | 4.31E-07 |  |  |  |  |  | 1 |  |  | 1 |  |  |
| cg08026195 |  |  |  |  |  |  | cg08026195 | C2orf27B | 4.91E-07 |  |  |  |  |  | 1 |  |  | 1 |  |  |
| cg15514751 |  |  |  |  |  |  | cg15514751 | CKS1B | 4.91E-07 |  |  |  |  |  | 1 |  |  | 1 |  |  |
| cg16636692 |  |  |  |  |  |  | cg16636692 | HIST1H2BK | 5.29E-07 |  |  |  |  |  | 1 |  |  | 1 |  |  |
| cg18731202 |  |  |  |  |  |  | cg18731202 | TGFA | 5.39E-07 |  |  |  |  |  | 1 |  |  | 1 |  |  |
| cg27649653 |  |  |  |  |  |  | cg27649653 | ZNF8 | 5.61E-07 |  |  |  |  |  | 1 |  |  | 1 |  |  |
| cg01344787 |  |  |  |  |  |  | cg01344787 |  | 5.75E-07 |  |  |  |  |  | 1 |  |  | 1 |  |  |
| cg00746487 |  |  |  |  |  |  | cg00746487 |  | 5.82E-07 |  |  |  |  |  | 1 |  |  | 1 |  |  |
| cg25808839 |  |  |  |  |  |  | cg25808839 | LACTB | 6.29E-07 |  |  |  |  |  | 1 |  |  | 1 |  |  |
| cg21763952 |  |  |  |  |  |  | cg21763952 | TCTN1 | 6.29E-07 |  |  |  |  |  | 1 |  |  | 1 |  |  |
| cg03811905 |  |  |  |  |  |  | cg03811905 |  | 6.29E-07 |  |  |  |  |  | 1 |  |  | 1 |  |  |
| cg13440641 |  |  |  |  |  |  | cg13440641 | DMTF1 | 7.64E-07 |  |  |  |  |  | 1 |  |  | 1 |  |  |
| cg22001073 |  |  |  |  |  |  | cg22001073 | RICS | 7.64E-07 |  |  |  |  |  | 1 |  |  | 1 |  |  |
| cg10351795 |  |  |  |  |  |  | cg10351795 | SLCO2A1 | 8.29E-07 |  |  |  |  |  | 1 |  |  | 1 |  |  |
| cg00095276 |  |  |  |  |  |  | cg00095276 | SLC12A7 | 8.38E-07 |  |  |  |  |  | 1 |  |  | 1 |  |  |
| cg13668025 |  |  |  |  |  |  | cg13668025 | SUCLG2 | 8.57E-07 |  |  |  |  |  | 1 |  |  | 1 |  |  |
| cg05874167 |  |  |  |  |  |  | cg05874167 |  | 8.57E-07 |  |  |  |  |  | 1 |  |  | 1 |  |  |
| cg08621418 |  |  |  |  |  |  | cg08621418 | HMGN3 | 9.53E-07 |  |  |  |  |  | 1 |  |  | 1 |  |  |
| cg23267554 |  |  |  |  |  |  | cg23267554 |  | 9.87E-07 |  |  |  |  |  | 1 |  |  | 1 |  |  |
| cg19550439 |  |  |  |  |  |  | cg19550439 | ADAMTS6 | 1.01E-06 |  |  |  |  |  | 1 |  |  | 1 |  |  |
| cg03046325 |  |  |  |  |  |  | cg03046325 | TNKS2 | 1.01E-06 |  |  |  |  |  | 1 |  |  | 1 |  |  |
| cg22403154 |  |  |  |  |  |  | cg22403154 |  | 1.01E-06 |  |  |  |  |  | 1 |  |  | 1 |  |  |
| cg14195606 |  |  |  |  |  |  | cg14195606 | FAM190A | 1.02E-06 |  |  |  |  |  | 1 |  |  | 1 |  |  |
| cg01612443 |  |  |  |  |  |  | cg01612443 | ATOH1 | 1.03E-06 |  |  |  |  |  | 1 |  |  | 1 |  |  |
| cg14791530 |  |  |  |  |  |  | cg14791530 | CTBS | 1.05E-06 |  |  |  |  |  | 1 |  |  | 1 |  |  |
| cg03482600 |  |  |  |  |  |  | cg03482600 |  | 1.08E-06 |  |  |  |  |  | 1 |  |  | 1 |  |  |
| cg09552983 |  |  |  |  |  |  | cg09552983 |  | 1.08E-06 |  |  |  |  |  | 1 |  |  | 1 |  |  |
| cg18968279 |  |  |  |  |  |  | cg18968279 | TCF7L2 | 1.11E-06 |  |  |  |  |  | 1 |  |  | 1 |  |  |
| cg02641288 |  |  |  |  |  |  | cg02641288 | IRX4 | 1.13E-06 |  |  |  |  |  | 1 |  |  | 1 |  |  |
| cg25354716 |  |  |  |  |  |  | cg25354716 | CRTAP | 1.14E-06 |  |  |  |  |  | 1 |  |  | 1 |  |  |
| cg01097768 | cg01097768 | AHRR | 1.22E+03 |  |  |  | cg01097768 | AHRR | 1.18E-06 |  | 1 |  |  |  |  | 1 |  | 1 |  |  |
| cg17632028 |  |  |  |  |  |  | cg17632028 | PDE6B | 1.19E-06 |  |  |  |  |  | 1 |  |  | 1 |  |  |
| cg26546646 |  |  |  |  |  |  | cg26546646 | ENSA | 1.30E-06 |  |  |  |  |  | 1 |  |  | 1 |  |  |
| cg18994438 |  |  |  |  |  |  | cg18994438 | FMNL1 | 1.37E-06 |  |  |  |  |  | 1 |  |  | 1 |  |  |
| cg26674132 |  |  |  |  |  |  | cg26674132 | ZNF559 | 1.40E-06 |  |  |  |  |  | 1 |  |  | 1 |  |  |
| cg23797200 |  |  |  |  |  |  | cg23797200 | NKIRAS2 | 1.43E-06 |  |  |  |  |  | 1 |  |  | 1 |  |  |
| cg01693063 |  |  |  |  |  |  | cg01693063 | PRMT8 | 1.43E-06 |  |  |  |  |  | 1 |  |  | 1 |  |  |
| cg11804350 |  |  |  |  |  |  | cg11804350 | VWF | 1.43E-06 |  |  |  |  |  | 1 |  |  | 1 |  |  |
| cg16149164 |  |  |  |  |  |  | cg16149164 | JAG2 | 1.46E-06 |  |  |  |  |  | 1 |  |  | 1 |  |  |
| cg11580351 |  |  |  |  |  |  | cg11580351 | SPATA9 | 1.48E-06 |  |  |  |  |  | 1 |  |  | 1 |  |  |
| cg23003872 |  |  |  |  |  |  | cg23003872 |  | 1.48E-06 |  |  |  |  |  | 1 |  |  | 1 |  |  |
| cg22995176 |  |  |  |  |  |  | cg22995176 | UPK3B | 1.49E-06 |  |  |  |  |  | 1 |  |  | 1 |  |  |
| cg18754985 |  |  |  |  |  |  | cg18754985 | CLDND1 | 1.51E-06 |  |  |  |  |  | 1 |  |  | 1 |  |  |
| cg05587870 |  |  |  |  |  |  | cg05587870 |  | 1.56E-06 |  |  |  |  |  | 1 |  |  | 1 |  |  |
| cg19358594 |  |  |  |  |  |  | cg19358594 | DFFA | 1.64E-06 |  |  |  |  |  | 1 |  |  | 1 |  |  |
| cg24186711 |  |  |  |  |  |  | cg24186711 | GUSBL1 | 1.64E-06 |  |  |  |  |  | 1 |  |  | 1 |  |  |
| cg17930737 |  |  |  |  |  |  | cg17930737 | NOP58 | 1.64E-06 |  |  |  |  |  | 1 |  |  | 1 |  |  |
| cg04179819 |  |  |  |  |  |  | cg04179819 | TAF3 | 1.64E-06 |  |  |  |  |  | 1 |  |  | 1 |  |  |
| cg10646962 |  |  |  |  |  |  | cg10646962 |  | 1.64E-06 |  |  |  |  |  | 1 |  |  | 1 |  |  |
| cg12992443 |  |  |  |  |  |  | cg12992443 | DLGAP4 | 1.66E-06 |  |  |  |  |  | 1 |  |  | 1 |  |  |
| cg26195710 |  |  |  |  |  |  | cg26195710 | GNRH1 | 1.66E-06 |  |  |  |  |  | 1 |  |  | 1 |  |  |
| cg03384915 |  |  |  |  |  |  | cg03384915 | SIN3B | 1.66E-06 |  |  |  |  |  | 1 |  |  | 1 |  |  |
| cg27356115 |  |  |  |  |  |  | cg27356115 |  | 1.66E-06 |  |  |  |  |  | 1 |  |  | 1 |  |  |
| cg04194664 |  |  |  |  |  |  | cg04194664 | C17orf69 | 1.67E-06 |  |  |  |  |  | 1 |  |  | 1 |  |  |
| cg10430189 |  |  |  |  |  |  | cg10430189 |  | 1.67E-06 |  |  |  |  |  | 1 |  |  | 1 |  |  |
| cg09606015 |  |  |  |  |  |  | cg09606015 | ATP11B | 1.71E-06 |  |  |  |  |  | 1 |  |  | 1 |  |  |
| cg04950839 |  |  |  |  |  |  | cg04950839 | HAT1 | 1.72E-06 |  |  |  |  |  | 1 |  |  | 1 |  |  |
| cg22182287 |  |  |  |  |  |  | cg22182287 | TTC15 | 1.76E-06 |  |  |  |  |  | 1 |  |  | 1 |  |  |
| cg10094624 |  |  |  |  |  |  | cg10094624 | ARL6IP1 | 1.78E-06 |  |  |  |  |  | 1 |  |  | 1 |  |  |
| cg17267720 |  |  |  |  |  |  | cg17267720 | PGBD4 | 1.81E-06 |  |  |  |  |  | 1 |  |  | 1 |  |  |
| cg07404400 |  |  |  |  |  |  | cg07404400 | TRIM41 | 1.86E-06 |  |  |  |  |  | 1 |  |  | 1 |  |  |
| cg17971328 |  |  |  |  |  |  | cg17971328 | SEMA4A | 2.03E-06 |  |  |  |  |  | 1 |  |  | 1 |  |  |
| cg02578836 |  |  |  |  |  |  | cg02578836 |  | 2.03E-06 |  |  |  |  |  | 1 |  |  | 1 |  |  |
| cg08828868 |  |  |  |  |  |  | cg08828868 | DDX21 | 2.08E-06 |  |  |  |  |  | 1 |  |  | 1 |  |  |
| cg11550862 |  |  |  |  |  |  | cg11550862 | NCOR2 | 2.23E-06 |  |  |  |  |  | 1 |  |  | 1 |  |  |
| cg10964388 |  |  |  |  |  |  | cg10964388 | NTN4 | 2.23E-06 |  |  |  |  |  | 1 |  |  | 1 |  |  |
| cg16709512 |  |  |  |  |  |  | cg16709512 |  | 2.23E-06 |  |  |  |  |  | 1 |  |  | 1 |  |  |
| cg26057840 |  |  |  |  |  |  | cg26057840 |  | 2.31E-06 |  |  |  |  |  | 1 |  |  | 1 |  |  |
| cg00155844 |  |  |  |  |  |  | cg00155844 | HERC2 | 2.41E-06 |  |  |  |  |  | 1 |  |  | 1 |  |  |
| cg05920998 |  |  |  |  |  |  | cg05920998 | HUS1B | 2.61E-06 |  |  |  |  |  | 1 |  |  | 1 |  |  |
| cg13675319 |  |  |  |  |  |  | cg13675319 |  | 2.62E-06 |  |  |  |  |  | 1 |  |  | 1 |  |  |
| cg06378498 |  |  |  |  |  |  | cg06378498 | STAT3 | 2.72E-06 |  |  |  |  |  | 1 |  |  | 1 |  |  |
| cg08360253 |  |  |  |  |  |  | cg08360253 | PPP2CA | 2.79E-06 |  |  |  |  |  | 1 |  |  | 1 |  |  |
| cg24793014 |  |  |  |  |  |  | cg24793014 | SNORA59B | 2.80E-06 |  |  |  |  |  | 1 |  |  | 1 |  |  |
| cg13324357 |  |  |  |  |  |  | cg13324357 |  | 2.80E-06 |  |  |  |  |  | 1 |  |  | 1 |  |  |
| cg21720999 |  |  |  |  |  |  | cg21720999 |  | 2.83E-06 |  |  |  |  |  | 1 |  |  | 1 |  |  |
| cg06936779 |  |  |  |  |  |  | cg06936779 | PIP5K1A | 2.91E-06 |  |  |  |  |  | 1 |  |  | 1 |  |  |
| cg26270695 |  |  |  |  |  |  | cg26270695 | CRABP1 | 2.95E-06 |  |  |  |  |  | 1 |  |  | 1 |  |  |
| cg23367119 |  |  |  |  |  |  | cg23367119 | C14orf43 | 3.08E-06 |  |  |  |  |  | 1 |  |  | 1 |  |  |
| cg23611710 |  |  |  |  |  |  | cg23611710 |  | 3.08E-06 |  |  |  |  |  | 1 |  |  | 1 |  |  |
| cg13576178 |  |  |  |  |  |  | cg13576178 | ZNF324B | 3.13E-06 |  |  |  |  |  | 1 |  |  | 1 |  |  |
| cg19716125 |  |  |  |  |  |  | cg19716125 |  | 3.13E-06 |  |  |  |  |  | 1 |  |  | 1 |  |  |
| cg10588834 |  |  |  |  |  |  | cg10588834 | AUTS2 | 3.14E-06 |  |  |  |  |  | 1 |  |  | 1 |  |  |
| cg21385983 |  |  |  |  |  |  | cg21385983 | PVALB | 3.14E-06 |  |  |  |  |  | 1 |  |  | 1 |  |  |
| cg14941559 |  |  |  |  |  |  | cg14941559 | NCK1 | 3.19E-06 |  |  |  |  |  | 1 |  |  | 1 |  |  |
| cg08866608 |  |  |  |  |  |  | cg08866608 |  | 3.32E-06 |  |  |  |  |  | 1 |  |  | 1 |  |  |
| cg15010854 |  |  |  |  |  |  | cg15010854 | BAIAP2 | 3.34E-06 |  |  |  |  |  | 1 |  |  | 1 |  |  |
| cg21898708 |  |  |  |  |  |  | cg21898708 | C6orf48 | 3.34E-06 |  |  |  |  |  | 1 |  |  | 1 |  |  |
| cg25362525 |  |  |  |  |  |  | cg25362525 |  | 3.34E-06 |  |  |  |  |  | 1 |  |  | 1 |  |  |
| cg26456259 |  |  |  |  |  |  | cg26456259 |  | 3.34E-06 |  |  |  |  |  | 1 |  |  | 1 |  |  |
| cg19243391 |  |  |  |  |  |  | cg19243391 | EIF4B | 3.36E-06 |  |  |  |  |  | 1 |  |  | 1 |  |  |
| cg22094163 |  |  |  |  |  |  | cg22094163 | KDM3A | 3.36E-06 |  |  |  |  |  | 1 |  |  | 1 |  |  |
| cg17512382 |  |  |  |  |  |  | cg17512382 |  | 3.36E-06 |  |  |  |  |  | 1 |  |  | 1 |  |  |
| cg17731547 |  |  |  |  |  |  | cg17731547 | COL23A1 | 3.57E-06 |  |  |  |  |  | 1 |  |  | 1 |  |  |
| cg21290290 |  |  |  |  |  |  | cg21290290 | C1orf93 | 3.60E-06 |  |  |  |  |  | 1 |  |  | 1 |  |  |
| cg05573133 |  |  |  |  |  |  | cg05573133 | FYCO1 | 3.60E-06 |  |  |  |  |  | 1 |  |  | 1 |  |  |
| cg01837661 |  |  |  |  |  |  | cg01837661 | GNB5 | 3.60E-06 |  |  |  |  |  | 1 |  |  | 1 |  |  |
| cg27622633 |  |  |  |  |  |  | cg27622633 | USP53 | 3.60E-06 |  |  |  |  |  | 1 |  |  | 1 |  |  |
| cg14651082 |  |  |  |  |  |  | cg14651082 |  | 3.60E-06 |  |  |  |  |  | 1 |  |  | 1 |  |  |
| cg18655915 |  |  |  |  |  |  | cg18655915 |  | 3.60E-06 |  |  |  |  |  | 1 |  |  | 1 |  |  |
| cg03603505 |  |  |  |  |  |  | cg03603505 | CLIP1 | 3.62E-06 |  |  |  |  |  | 1 |  |  | 1 |  |  |
| cg00690392 |  |  |  |  |  |  | cg00690392 | ENAH | 3.66E-06 |  |  |  |  |  | 1 |  |  | 1 |  |  |
| cg07065737 |  |  |  |  |  |  | cg07065737 |  | 3.66E-06 |  |  |  |  |  | 1 |  |  | 1 |  |  |
| cg17917920 |  |  |  |  |  |  | cg17917920 |  | 3.66E-06 |  |  |  |  |  | 1 |  |  | 1 |  |  |
| cg11168432 |  |  |  |  |  |  | cg11168432 | MAEA | 3.67E-06 |  |  |  |  |  | 1 |  |  | 1 |  |  |
| cg18474718 |  |  |  |  |  |  | cg18474718 | ATM | 3.74E-06 |  |  |  |  |  | 1 |  |  | 1 |  |  |
| cg12880967 |  |  |  |  |  |  | cg12880967 | MACROD1 | 3.74E-06 |  |  |  |  |  | 1 |  |  | 1 |  |  |
| cg12950624 |  |  |  |  |  |  | cg12950624 | RTN2 | 3.74E-06 |  |  |  |  |  | 1 |  |  | 1 |  |  |
| cg26182263 |  |  |  |  |  |  | cg26182263 | SLC39A14 | 3.74E-06 |  |  |  |  |  | 1 |  |  | 1 |  |  |
| cg02247175 |  |  |  |  |  |  | cg02247175 |  | 3.83E-06 |  |  |  |  |  | 1 |  |  | 1 |  |  |
| cg17413252 |  |  |  |  |  |  | cg17413252 | ENPP1 | 3.88E-06 |  |  |  |  |  | 1 |  |  | 1 |  |  |
| cg01852611 |  |  |  |  |  |  | cg01852611 | DSCR8 | 3.92E-06 |  |  |  |  |  | 1 |  |  | 1 |  |  |
| cg12484845 |  |  |  |  |  |  | cg12484845 | ZNF826 | 3.96E-06 |  |  |  |  |  | 1 |  |  | 1 |  |  |
| cg02111705 |  |  |  |  |  |  | cg02111705 |  | 4.06E-06 |  |  |  |  |  | 1 |  |  | 1 |  |  |
| cg11809157 |  |  |  |  |  |  | cg11809157 | BBC3 | 4.08E-06 |  |  |  |  |  | 1 |  |  | 1 |  |  |
| cg00833661 |  |  |  |  |  |  | cg00833661 | KCNE1 | 4.08E-06 |  |  |  |  |  | 1 |  |  | 1 |  |  |
| cg08451992 |  |  |  |  |  |  | cg08451992 | ARMC5 | 4.16E-06 |  |  |  |  |  | 1 |  |  | 1 |  |  |
| cg22924269 |  |  |  |  |  |  | cg22924269 | PHF11 | 4.16E-06 |  |  |  |  |  | 1 |  |  | 1 |  |  |
| cg12317505 |  |  |  |  |  |  | cg12317505 | CUBN | 4.33E-06 |  |  |  |  |  | 1 |  |  | 1 |  |  |
| cg04042800 |  |  |  |  |  |  | cg04042800 |  | 4.38E-06 |  |  |  |  |  | 1 |  |  | 1 |  |  |
| cg01638829 |  |  |  |  |  |  | cg01638829 |  | 4.49E-06 |  |  |  |  |  | 1 |  |  | 1 |  |  |
| cg21249729 |  |  |  |  |  |  | cg21249729 | C9orf116 | 4.50E-06 |  |  |  |  |  | 1 |  |  | 1 |  |  |
| cg08283318 |  |  |  |  |  |  | cg08283318 | ETFB | 4.50E-06 |  |  |  |  |  | 1 |  |  | 1 |  |  |
| cg02671915 |  |  |  |  |  |  | cg02671915 | MAL2 | 4.50E-06 |  |  |  |  |  | 1 |  |  | 1 |  |  |
| cg14905634 |  |  |  |  |  |  | cg14905634 | TRHDE | 4.50E-06 |  |  |  |  |  | 1 |  |  | 1 |  |  |
| cg00642607 |  |  |  |  |  |  | cg00642607 |  | 4.50E-06 |  |  |  |  |  | 1 |  |  | 1 |  |  |
| cg04031757 |  |  |  |  |  |  | cg04031757 |  | 4.50E-06 |  |  |  |  |  | 1 |  |  | 1 |  |  |
| cg09011231 |  |  |  |  |  |  | cg09011231 | NCRNA00164 | 4.58E-06 |  |  |  |  |  | 1 |  |  | 1 |  |  |
| cg11140305 |  |  |  |  |  |  | cg11140305 | SH3BP5L | 4.58E-06 |  |  |  |  |  | 1 |  |  | 1 |  |  |
| cg26311995 |  |  |  |  |  |  | cg26311995 | TRIM26 | 4.58E-06 |  |  |  |  |  | 1 |  |  | 1 |  |  |
| cg05620791 |  |  |  |  |  |  | cg05620791 |  | 4.58E-06 |  |  |  |  |  | 1 |  |  | 1 |  |  |
| cg20891060 |  |  |  |  |  |  | cg20891060 |  | 4.58E-06 |  |  |  |  |  | 1 |  |  | 1 |  |  |
| cg21450008 |  |  |  |  |  |  | cg21450008 |  | 4.58E-06 |  |  |  |  |  | 1 |  |  | 1 |  |  |
| cg02171500 |  |  |  |  |  |  | cg02171500 | CHKA | 4.72E-06 |  |  |  |  |  | 1 |  |  | 1 |  |  |
| cg08531017 |  |  |  |  |  |  | cg08531017 | DSCAML1 | 4.72E-06 |  |  |  |  |  | 1 |  |  | 1 |  |  |
| cg02392575 |  |  |  |  |  |  | cg02392575 | UHRF1BP1L | 4.72E-06 |  |  |  |  |  | 1 |  |  | 1 |  |  |
| cg07972458 |  |  |  |  |  |  | cg07972458 |  | 4.72E-06 |  |  |  |  |  | 1 |  |  | 1 |  |  |
| cg21389924 |  |  |  |  |  |  | cg21389924 |  | 4.72E-06 |  |  |  |  |  | 1 |  |  | 1 |  |  |
| cg11843502 |  |  |  |  |  |  | cg11843502 | PER3 | 4.77E-06 |  |  |  |  |  | 1 |  |  | 1 |  |  |
| cg22225065 |  |  |  |  |  |  | cg22225065 | C1orf57 | 4.92E-06 |  |  |  |  |  | 1 |  |  | 1 |  |  |
| cg07334509 |  |  |  |  |  |  | cg07334509 | STAU2 | 4.92E-06 |  |  |  |  |  | 1 |  |  | 1 |  |  |
| cg27528104 |  |  |  |  |  |  | cg27528104 | LOC100128023 | 4.93E-06 |  |  |  |  |  | 1 |  |  | 1 |  |  |
| cg02720697 |  |  |  |  |  |  | cg02720697 | NFIC | 4.93E-06 |  |  |  |  |  | 1 |  |  | 1 |  |  |
| cg22496377 |  |  |  |  |  |  | cg22496377 | SHF | 4.99E-06 |  |  |  |  |  | 1 |  |  | 1 |  |  |
| cg24518943 |  |  |  |  |  |  | cg24518943 | FAM135A | 5.00E-06 |  |  |  |  |  | 1 |  |  | 1 |  |  |
| cg11565042 |  |  |  |  |  |  | cg11565042 | GPR45 | 5.00E-06 |  |  |  |  |  | 1 |  |  | 1 |  |  |
| cg18801567 |  |  |  |  |  |  | cg18801567 | PDCD10 | 5.23E-06 |  |  |  |  |  | 1 |  |  | 1 |  |  |
| cg05347948 |  |  |  |  |  |  | cg05347948 |  | 5.23E-06 |  |  |  |  |  | 1 |  |  | 1 |  |  |
| cg18016288 |  |  |  |  |  |  | cg18016288 | ABCC4 | 5.24E-06 |  |  |  |  |  | 1 |  |  | 1 |  |  |
| cg16922869 |  |  |  |  |  |  | cg16922869 | H1FOO | 5.24E-06 |  |  |  |  |  | 1 |  |  | 1 |  |  |
| cg11206312 |  |  |  |  |  |  | cg11206312 | FGF8 | 5.31E-06 |  |  |  |  |  | 1 |  |  | 1 |  |  |
| cg19213194 |  |  |  |  |  |  | cg19213194 | CUX1 | 5.38E-06 |  |  |  |  |  | 1 |  |  | 1 |  |  |
| cg08668662 |  |  |  |  |  |  | cg08668662 | PDRG1 | 5.52E-06 |  |  |  |  |  | 1 |  |  | 1 |  |  |
| cg08750493 |  |  |  |  |  |  | cg08750493 | ZNF204P | 5.52E-06 |  |  |  |  |  | 1 |  |  | 1 |  |  |
| cg04140754 |  |  |  |  |  |  | cg04140754 | ATP6V0A2 | 5.56E-06 |  |  |  |  |  | 1 |  |  | 1 |  |  |
| cg24301350 |  |  |  |  |  |  | cg24301350 | C2orf69 | 5.64E-06 |  |  |  |  |  | 1 |  |  | 1 |  |  |
| cg24311704 |  |  |  |  |  |  | cg24311704 | MUC21 | 5.64E-06 |  |  |  |  |  | 1 |  |  | 1 |  |  |
| cg08488494 |  |  |  |  |  |  | cg08488494 | ZNF365 | 5.64E-06 |  |  |  |  |  | 1 |  |  | 1 |  |  |
| cg14719752 |  |  |  |  |  |  | cg14719752 |  | 5.64E-06 |  |  |  |  |  | 1 |  |  | 1 |  |  |
| cg16812288 |  |  |  |  |  |  | cg16812288 |  | 5.64E-06 |  |  |  |  |  | 1 |  |  | 1 |  |  |
| cg23987549 |  |  |  |  |  |  | cg23987549 | PMEPA1 | 5.66E-06 |  |  |  |  |  | 1 |  |  | 1 |  |  |
| cg06438056 |  |  |  |  |  |  | cg06438056 | AK2 | 5.70E-06 |  |  |  |  |  | 1 |  |  | 1 |  |  |
| cg21192376 |  |  |  |  |  |  | cg21192376 | GPR137C | 5.70E-06 |  |  |  |  |  | 1 |  |  | 1 |  |  |
| cg14743683 |  |  |  |  |  |  | cg14743683 | PTPRN2 | 5.71E-06 |  |  |  |  |  | 1 |  |  | 1 |  |  |
| cg19104471 |  |  |  |  |  |  | cg19104471 | TOMM34 | 5.77E-06 |  |  |  |  |  | 1 |  |  | 1 |  |  |
| cg18864497 |  |  |  |  |  |  | cg18864497 | SSR1 | 5.84E-06 |  |  |  |  |  | 1 |  |  | 1 |  |  |
| cg09917026 |  |  |  |  |  |  | cg09917026 | GNPTG | 5.87E-06 |  |  |  |  |  | 1 |  |  | 1 |  |  |
| cg23674788 |  |  |  |  |  |  | cg23674788 | KRT32 | 5.87E-06 |  |  |  |  |  | 1 |  |  | 1 |  |  |
| cg19110795 |  |  |  |  |  |  | cg19110795 | UBXN7 | 6.05E-06 |  |  |  |  |  | 1 |  |  | 1 |  |  |
| cg01938570 |  |  |  |  |  |  | cg01938570 | ZBTB43 | 6.05E-06 |  |  |  |  |  | 1 |  |  | 1 |  |  |
| cg03467813 |  |  |  |  |  |  | cg03467813 | FAM50B | 6.09E-06 |  |  |  |  |  | 1 |  |  | 1 |  |  |
| cg04629194 |  |  |  |  |  |  | cg04629194 | PDLIM1 | 6.09E-06 |  |  |  |  |  | 1 |  |  | 1 |  |  |
| cg13762887 |  |  |  |  |  |  | cg13762887 | ZER1 | 6.09E-06 |  |  |  |  |  | 1 |  |  | 1 |  |  |
| cg18007641 |  |  |  |  |  |  | cg18007641 |  | 6.09E-06 |  |  |  |  |  | 1 |  |  | 1 |  |  |
| cg13566059 |  |  |  |  |  |  | cg13566059 | TFDP3 | 6.10E-06 |  |  |  |  |  | 1 |  |  | 1 |  |  |
| cg02569236 |  |  |  |  |  |  | cg02569236 | ALDH1L1 | 6.12E-06 |  |  |  |  |  | 1 |  |  | 1 |  |  |
| cg00649216 |  |  |  |  |  |  | cg00649216 |  | 6.13E-06 |  |  |  |  |  | 1 |  |  | 1 |  |  |
| cg06897921 |  |  |  |  |  |  | cg06897921 |  | 6.34E-06 |  |  |  |  |  | 1 |  |  | 1 |  |  |
| cg05079547 |  |  |  |  |  |  | cg05079547 | NTM | 6.46E-06 |  |  |  |  |  | 1 |  |  | 1 |  |  |
| cg12416053 |  |  |  |  |  |  | cg12416053 | ROBO2 | 6.46E-06 |  |  |  |  |  | 1 |  |  | 1 |  |  |
| cg22743003 |  |  |  |  |  |  | cg22743003 |  | 6.46E-06 |  |  |  |  |  | 1 |  |  | 1 |  |  |
| cg05707844 |  |  |  |  |  |  | cg05707844 | EIF2AK2 | 6.49E-06 |  |  |  |  |  | 1 |  |  | 1 |  |  |
| cg02292066 |  |  |  |  |  |  | cg02292066 | C1orf93 | 6.50E-06 |  |  |  |  |  | 1 |  |  | 1 |  |  |
| cg07598331 |  |  |  |  |  |  | cg07598331 | DMRTB1 | 6.50E-06 |  |  |  |  |  | 1 |  |  | 1 |  |  |
| cg14628803 |  |  |  |  |  |  | cg14628803 |  | 6.50E-06 |  |  |  |  |  | 1 |  |  | 1 |  |  |
| cg13461130 |  |  |  |  |  |  | cg13461130 | PNPLA7 | 6.53E-06 |  |  |  |  |  | 1 |  |  | 1 |  |  |
| cg27097575 |  |  |  |  |  |  | cg27097575 | ADARB2 | 6.56E-06 |  |  |  |  |  | 1 |  |  | 1 |  |  |
| cg24291974 |  |  |  |  |  |  | cg24291974 | PLIN5 | 6.56E-06 |  |  |  |  |  | 1 |  |  | 1 |  |  |
| cg21112148 |  |  |  |  |  |  | cg21112148 | FBXL12 | 6.59E-06 |  |  |  |  |  | 1 |  |  | 1 |  |  |
| cg02227015 |  |  |  |  |  |  | cg02227015 |  | 6.72E-06 |  |  |  |  |  | 1 |  |  | 1 |  |  |
| cg18641329 |  |  |  |  |  |  | cg18641329 | RCBTB2 | 6.87E-06 |  |  |  |  |  | 1 |  |  | 1 |  |  |
| cg08532673 |  |  |  |  |  |  | cg08532673 |  | 7.10E-06 |  |  |  |  |  | 1 |  |  | 1 |  |  |
| cg19906093 |  |  |  |  |  |  | cg19906093 | C2orf88 | 7.27E-06 |  |  |  |  |  | 1 |  |  | 1 |  |  |
| cg27521571 |  |  |  |  |  |  | cg27521571 | COMT | 7.27E-06 |  |  |  |  |  | 1 |  |  | 1 |  |  |
| cg10000843 |  |  |  |  |  |  | cg10000843 | FANCI | 7.30E-06 |  |  |  |  |  | 1 |  |  | 1 |  |  |
| cg18055230 |  |  |  |  |  |  | cg18055230 | FAM32A | 7.38E-06 |  |  |  |  |  | 1 |  |  | 1 |  |  |
| cg07500957 |  |  |  |  |  |  | cg07500957 | OTOR | 7.44E-06 |  |  |  |  |  | 1 |  |  | 1 |  |  |
| cg27573593 |  |  |  |  |  |  | cg27573593 | PIK3CD | 7.44E-06 |  |  |  |  |  | 1 |  |  | 1 |  |  |
| cg11213199 |  |  |  |  |  |  | cg11213199 | MRPS24 | 7.49E-06 |  |  |  |  |  | 1 |  |  | 1 |  |  |
| cg14082938 |  |  |  |  |  |  | cg14082938 |  | 7.57E-06 |  |  |  |  |  | 1 |  |  | 1 |  |  |
| cg22530977 |  |  |  |  |  |  | cg22530977 |  | 7.62E-06 |  |  |  |  |  | 1 |  |  | 1 |  |  |
| cg24804436 |  |  |  |  |  |  | cg24804436 | PPP2R5D | 7.88E-06 |  |  |  |  |  | 1 |  |  | 1 |  |  |
| cg21194937 |  |  |  |  |  |  | cg21194937 | AP3D1 | 8.02E-06 |  |  |  |  |  | 1 |  |  | 1 |  |  |
| cg21466736 |  |  |  |  |  |  | cg21466736 |  | 8.02E-06 |  |  |  |  |  | 1 |  |  | 1 |  |  |
| cg09999563 |  |  |  |  |  |  | cg09999563 | AASDH | 8.08E-06 |  |  |  |  |  | 1 |  |  | 1 |  |  |
| cg03662571 |  |  |  |  |  |  | cg03662571 | SLC16A12 | 8.08E-06 |  |  |  |  |  | 1 |  |  | 1 |  |  |
| cg16062877 |  |  |  |  |  |  | cg16062877 | KSR1 | 8.10E-06 |  |  |  |  |  | 1 |  |  | 1 |  |  |
| cg09908764 |  |  |  |  |  |  | cg09908764 |  | 8.17E-06 |  |  |  |  |  | 1 |  |  | 1 |  |  |
| cg18874902 |  |  |  |  |  |  | cg18874902 | CSNK1G3 | 8.22E-06 |  |  |  |  |  | 1 |  |  | 1 |  |  |
| cg12432846 |  |  |  |  |  |  | cg12432846 | BCL10 | 8.30E-06 |  |  |  |  |  | 1 |  |  | 1 |  |  |
| cg11649016 |  |  |  |  |  |  | cg11649016 | ITGA7 | 8.30E-06 |  |  |  |  |  | 1 |  |  | 1 |  |  |
| cg10216717 |  |  |  |  |  |  | cg10216717 | TMEM132C | 8.30E-06 |  |  |  |  |  | 1 |  |  | 1 |  |  |
| cg00727386 |  |  |  |  |  |  | cg00727386 |  | 8.30E-06 |  |  |  |  |  | 1 |  |  | 1 |  |  |
| cg03168249 |  |  |  |  |  |  | cg03168249 | KAZALD1 | 8.32E-06 |  |  |  |  |  | 1 |  |  | 1 |  |  |
| cg03775901 |  |  |  |  |  |  | cg03775901 | CNDP1 | 8.36E-06 |  |  |  |  |  | 1 |  |  | 1 |  |  |
| cg21552822 |  |  |  |  |  |  | cg21552822 | PDHX | 8.36E-06 |  |  |  |  |  | 1 |  |  | 1 |  |  |
| cg18570553 |  |  |  |  |  |  | cg18570553 | PRR15 | 8.52E-06 |  |  |  |  |  | 1 |  |  | 1 |  |  |
| cg27607583 |  |  |  |  |  |  | cg27607583 | TRAP1 | 8.62E-06 |  |  |  |  |  | 1 |  |  | 1 |  |  |
| cg10572794 |  |  |  |  |  |  | cg10572794 | ABHD6 | 8.65E-06 |  |  |  |  |  | 1 |  |  | 1 |  |  |
| cg07120806 |  |  |  |  |  |  | cg07120806 | WHSC1 | 8.77E-06 |  |  |  |  |  | 1 |  |  | 1 |  |  |
| cg16063474 |  |  |  |  |  |  | cg16063474 | STEAP3 | 8.85E-06 |  |  |  |  |  | 1 |  |  | 1 |  |  |
| cg03862705 |  |  |  |  |  |  | cg03862705 | NAT8B | 8.87E-06 |  |  |  |  |  | 1 |  |  | 1 |  |  |
| cg26573518 |  |  |  |  |  |  | cg26573518 | SLFN11 | 8.87E-06 |  |  |  |  |  | 1 |  |  | 1 |  |  |
| cg18556005 |  |  |  |  |  |  | cg18556005 | MGA | 8.88E-06 |  |  |  |  |  | 1 |  |  | 1 |  |  |
| cg20755651 |  |  |  |  |  |  | cg20755651 | SMOC2 | 8.89E-06 |  |  |  |  |  | 1 |  |  | 1 |  |  |
| cg24533466 |  |  |  |  |  |  | cg24533466 | SSBP3 | 9.14E-06 |  |  |  |  |  | 1 |  |  | 1 |  |  |
| cg17054485 |  |  |  |  |  |  | cg17054485 |  | 9.14E-06 |  |  |  |  |  | 1 |  |  | 1 |  |  |
| cg03761477 |  |  |  |  |  |  | cg03761477 |  | 9.20E-06 |  |  |  |  |  | 1 |  |  | 1 |  |  |
| cg23098168 |  |  |  |  |  |  | cg23098168 | CELSR2 | 9.32E-06 |  |  |  |  |  | 1 |  |  | 1 |  |  |
| cg17456644 |  |  |  |  |  |  | cg17456644 | KSR1 | 9.32E-06 |  |  |  |  |  | 1 |  |  | 1 |  |  |
| cg19017254 |  |  |  |  |  |  | cg19017254 | TRPM4 | 9.35E-06 |  |  |  |  |  | 1 |  |  | 1 |  |  |
| cg06056514 |  |  |  |  |  |  | cg06056514 | VARS | 9.36E-06 |  |  |  |  |  | 1 |  |  | 1 |  |  |
| cg18115235 |  |  |  |  |  |  | cg18115235 |  | 9.39E-06 |  |  |  |  |  | 1 |  |  | 1 |  |  |
| cg16608652 |  |  |  |  |  |  | cg16608652 | B3GALT2 | 9.41E-06 |  |  |  |  |  | 1 |  |  | 1 |  |  |
| cg22460590 |  |  |  |  |  |  | cg22460590 |  | 9.47E-06 |  |  |  |  |  | 1 |  |  | 1 |  |  |
| cg05153748 |  |  |  |  |  |  | cg05153748 |  | 9.49E-06 |  |  |  |  |  | 1 |  |  | 1 |  |  |
| cg26227186 |  |  |  |  |  |  | cg26227186 | CNN3 | 9.59E-06 |  |  |  |  |  | 1 |  |  | 1 |  |  |
| cg24170784 |  |  |  |  |  |  | cg24170784 | CABP7 | 9.75E-06 |  |  |  |  |  | 1 |  |  | 1 |  |  |
| cg01442959 |  |  |  |  |  |  | cg01442959 | HIST1H2AM | 9.77E-06 |  |  |  |  |  | 1 |  |  | 1 |  |  |
| cg03508409 |  |  |  |  |  |  | cg03508409 | PRR14 | 9.87E-06 |  |  |  |  |  | 1 |  |  | 1 |  |  |
| cg14515381 |  |  |  |  |  |  | cg14515381 | VCP | 1.01E-05 |  |  |  |  |  | 1 |  |  | 1 |  |  |
| cg05377515 |  |  |  |  |  |  | cg05377515 |  | 1.01E-05 |  |  |  |  |  | 1 |  |  | 1 |  |  |
| cg01746532 |  |  |  |  |  |  | cg01746532 | HSPBP1 | 1.02E-05 |  |  |  |  |  | 1 |  |  | 1 |  |  |
| cg20353780 |  |  |  |  |  |  | cg20353780 | TAOK3 | 1.02E-05 |  |  |  |  |  | 1 |  |  | 1 |  |  |
| cg08681117 |  |  |  |  |  |  | cg08681117 |  | 1.02E-05 |  |  |  |  |  | 1 |  |  | 1 |  |  |
| cg04995826 |  |  |  |  |  |  | cg04995826 | API5 | 1.03E-05 |  |  |  |  |  | 1 |  |  | 1 |  |  |
| cg25538883 |  |  |  |  |  |  | cg25538883 | MAPK4 | 1.05E-05 |  |  |  |  |  | 1 |  |  | 1 |  |  |
| cg16422343 |  |  |  |  |  |  | cg16422343 | SFRS3 | 1.05E-05 |  |  |  |  |  | 1 |  |  | 1 |  |  |
| cg26530713 |  |  |  |  |  |  | cg26530713 | CRTC2 | 1.06E-05 |  |  |  |  |  | 1 |  |  | 1 |  |  |
| cg07156182 |  |  |  |  |  |  | cg07156182 | DIRC2 | 1.12E-05 |  |  |  |  |  | 1 |  |  | 1 |  |  |
| cg21364231 |  |  |  |  |  |  | cg21364231 | LOC100128822 | 1.12E-05 |  |  |  |  |  | 1 |  |  | 1 |  |  |
| cg22459078 |  |  |  |  |  |  | cg22459078 |  | 1.13E-05 |  |  |  |  |  | 1 |  |  | 1 |  |  |
| cg26947060 |  |  |  |  |  |  | cg26947060 | ANKRD19 | 1.14E-05 |  |  |  |  |  | 1 |  |  | 1 |  |  |
| cg16361253 |  |  |  |  |  |  | cg16361253 | GPR111 | 1.15E-05 |  |  |  |  |  | 1 |  |  | 1 |  |  |
| cg23076299 |  |  |  |  |  |  | cg23076299 | PCDH21 | 1.15E-05 |  |  |  |  |  | 1 |  |  | 1 |  |  |
| cg09387992 |  |  |  |  |  |  | cg09387992 |  | 1.16E-05 |  |  |  |  |  | 1 |  |  | 1 |  |  |
| cg07991479 |  |  |  |  |  |  | cg07991479 | KAT2A | 1.17E-05 |  |  |  |  |  | 1 |  |  | 1 |  |  |
| cg01341751 |  |  |  |  |  |  | cg01341751 | THRA | 1.17E-05 |  |  |  |  |  | 1 |  |  | 1 |  |  |
| cg25155298 |  |  |  |  |  |  | cg25155298 | TRERF1 | 1.17E-05 |  |  |  |  |  | 1 |  |  | 1 |  |  |
| cg21963656 |  |  |  |  |  |  | cg21963656 | VARS | 1.19E-05 |  |  |  |  |  | 1 |  |  | 1 |  |  |
| cg24538512 |  |  |  |  |  |  | cg24538512 | NFATC1 | 1.20E-05 |  |  |  |  |  | 1 |  |  | 1 |  |  |
| cg13958324 |  |  |  |  |  |  | cg13958324 | ARID5B | 1.21E-05 |  |  |  |  |  | 1 |  |  | 1 |  |  |
| cg03562528 |  |  |  |  |  |  | cg03562528 | ASB2 | 1.21E-05 |  |  |  |  |  | 1 |  |  | 1 |  |  |
| cg14170437 |  |  |  |  |  |  | cg14170437 | CD300C | 1.21E-05 |  |  |  |  |  | 1 |  |  | 1 |  |  |
| cg11343534 |  |  |  |  |  |  | cg11343534 | SETD1B | 1.21E-05 |  |  |  |  |  | 1 |  |  | 1 |  |  |
| cg03244189 |  |  |  |  |  |  | cg03244189 | TTTY14 | 1.21E-05 |  |  |  |  |  | 1 |  |  | 1 |  |  |
| cg02693345 |  |  |  |  |  |  | cg02693345 |  | 1.21E-05 |  |  |  |  |  | 1 |  |  | 1 |  |  |
| cg03038850 |  |  |  |  |  |  | cg03038850 |  | 1.21E-05 |  |  |  |  |  | 1 |  |  | 1 |  |  |
| cg06213807 |  |  |  |  |  |  | cg06213807 |  | 1.21E-05 |  |  |  |  |  | 1 |  |  | 1 |  |  |
| cg08128444 |  |  |  |  |  |  | cg08128444 |  | 1.21E-05 |  |  |  |  |  | 1 |  |  | 1 |  |  |
| cg26698819 |  |  |  |  |  |  | cg26698819 |  | 1.21E-05 |  |  |  |  |  | 1 |  |  | 1 |  |  |
| cg16650002 |  |  |  |  |  |  | cg16650002 | C7orf57 | 1.22E-05 |  |  |  |  |  | 1 |  |  | 1 |  |  |
| cg20873046 |  |  |  |  |  |  | cg20873046 | KDM4A | 1.22E-05 |  |  |  |  |  | 1 |  |  | 1 |  |  |
| cg01249134 |  |  |  |  |  |  | cg01249134 |  | 1.22E-05 |  |  |  |  |  | 1 |  |  | 1 |  |  |
| cg06431105 |  |  |  |  |  |  | cg06431105 | ELL | 1.23E-05 |  |  |  |  |  | 1 |  |  | 1 |  |  |
| cg03525818 |  |  |  |  |  |  | cg03525818 |  | 1.24E-05 |  |  |  |  |  | 1 |  |  | 1 |  |  |
| cg00608779 |  |  |  |  |  |  | cg00608779 | PRRX2 | 1.26E-05 |  |  |  |  |  | 1 |  |  | 1 |  |  |
| cg21126306 |  |  |  |  |  |  | cg21126306 |  | 1.26E-05 |  |  |  |  |  | 1 |  |  | 1 |  |  |
| cg16727862 |  |  |  |  |  |  | cg16727862 | ATP10A | 1.27E-05 |  |  |  |  |  | 1 |  |  | 1 |  |  |
| cg01418536 |  |  |  |  |  |  | cg01418536 | USP32 | 1.27E-05 |  |  |  |  |  | 1 |  |  | 1 |  |  |
| cg25411699 |  |  |  |  |  |  | cg25411699 | ELMO1 | 1.29E-05 |  |  |  |  |  | 1 |  |  | 1 |  |  |
| cg18260823 |  |  |  |  |  |  | cg18260823 |  | 1.29E-05 |  |  |  |  |  | 1 |  |  | 1 |  |  |
| cg26061593 |  |  |  |  |  |  | cg26061593 |  | 1.29E-05 |  |  |  |  |  | 1 |  |  | 1 |  |  |
| cg11253957 |  |  |  |  |  |  | cg11253957 | MYO18A | 1.30E-05 |  |  |  |  |  | 1 |  |  | 1 |  |  |
| cg18715665 |  |  |  |  |  |  | cg18715665 |  | 1.30E-05 |  |  |  |  |  | 1 |  |  | 1 |  |  |
| cg26319015 |  |  |  |  |  |  | cg26319015 |  | 1.30E-05 |  |  |  |  |  | 1 |  |  | 1 |  |  |
| cg02264922 |  |  |  |  |  |  | cg02264922 | B4GALNT1 | 1.31E-05 |  |  |  |  |  | 1 |  |  | 1 |  |  |
| cg18575346 |  |  |  |  |  |  | cg18575346 | EP400 | 1.31E-05 |  |  |  |  |  | 1 |  |  | 1 |  |  |
| cg06557376 |  |  |  |  |  |  | cg06557376 | MYH10 | 1.31E-05 |  |  |  |  |  | 1 |  |  | 1 |  |  |
| cg09043104 |  |  |  |  |  |  | cg09043104 |  | 1.31E-05 |  |  |  |  |  | 1 |  |  | 1 |  |  |
| cg00486022 |  |  |  |  |  |  | cg00486022 | KIFC3 | 1.32E-05 |  |  |  |  |  | 1 |  |  | 1 |  |  |
| cg02322048 |  |  |  |  |  |  | cg02322048 | PIP5K1C | 1.32E-05 |  |  |  |  |  | 1 |  |  | 1 |  |  |
| cg15888699 |  |  |  |  |  |  | cg15888699 | TIMM10 | 1.32E-05 |  |  |  |  |  | 1 |  |  | 1 |  |  |
| cg16608731 |  |  |  |  |  |  | cg16608731 | PIPOX | 1.33E-05 |  |  |  |  |  | 1 |  |  | 1 |  |  |
| cg01405684 |  |  |  |  |  |  | cg01405684 | TBC1D10B | 1.34E-05 |  |  |  |  |  | 1 |  |  | 1 |  |  |
| cg03651715 |  |  |  |  |  |  | cg03651715 | AKAP12 | 1.35E-05 |  |  |  |  |  | 1 |  |  | 1 |  |  |
| cg18504937 |  |  |  |  |  |  | cg18504937 | KLK9 | 1.35E-05 |  |  |  |  |  | 1 |  |  | 1 |  |  |
| cg19655032 |  |  |  |  |  |  | cg19655032 |  | 1.35E-05 |  |  |  |  |  | 1 |  |  | 1 |  |  |
| cg10858195 |  |  |  |  |  |  | cg10858195 |  | 1.39E-05 |  |  |  |  |  | 1 |  |  | 1 |  |  |
| cg12090052 |  |  |  |  |  |  | cg12090052 | TCP11 | 1.40E-05 |  |  |  |  |  | 1 |  |  | 1 |  |  |
| cg23094080 |  |  |  |  |  |  | cg23094080 | DGAT1 | 1.42E-05 |  |  |  |  |  | 1 |  |  | 1 |  |  |
| cg14056470 |  |  |  |  |  |  | cg14056470 | DYRK1B | 1.43E-05 |  |  |  |  |  | 1 |  |  | 1 |  |  |
| cg00712792 |  |  |  |  |  |  | cg00712792 | SPIRE2 | 1.43E-05 |  |  |  |  |  | 1 |  |  | 1 |  |  |
| cg06936402 |  |  |  |  |  |  | cg06936402 | MMP20 | 1.45E-05 |  |  |  |  |  | 1 |  |  | 1 |  |  |
| cg14964274 |  |  |  |  |  |  | cg14964274 | USH2A | 1.45E-05 |  |  |  |  |  | 1 |  |  | 1 |  |  |
| cg04607032 |  |  |  |  |  |  | cg04607032 |  | 1.45E-05 |  |  |  |  |  | 1 |  |  | 1 |  |  |
| cg20668718 |  |  |  |  |  |  | cg20668718 | JAKMIP2 | 1.46E-05 |  |  |  |  |  | 1 |  |  | 1 |  |  |
| cg22067527 |  |  |  |  |  |  | cg22067527 | RFX2 | 1.49E-05 |  |  |  |  |  | 1 |  |  | 1 |  |  |
| cg24003539 |  |  |  |  |  |  | cg24003539 |  | 1.49E-05 |  |  |  |  |  | 1 |  |  | 1 |  |  |
| cg12145289 |  |  |  |  |  |  | cg12145289 | PCNXL3 | 1.52E-05 |  |  |  |  |  | 1 |  |  | 1 |  |  |
| cg25840536 |  |  |  |  |  |  | cg25840536 | RBPMS2 | 1.54E-05 |  |  |  |  |  | 1 |  |  | 1 |  |  |
| cg13412452 |  |  |  |  |  |  | cg13412452 | DDX31 | 1.55E-05 |  |  |  |  |  | 1 |  |  | 1 |  |  |
| cg05287481 |  |  |  |  |  |  | cg05287481 |  | 1.55E-05 |  |  |  |  |  | 1 |  |  | 1 |  |  |
| cg04715525 |  |  |  |  |  |  | cg04715525 | ARGLU1 | 1.56E-05 |  |  |  |  |  | 1 |  |  | 1 |  |  |
| cg21078247 |  |  |  |  |  |  | cg21078247 | CAMK2D | 1.56E-05 |  |  |  |  |  | 1 |  |  | 1 |  |  |
| cg26440142 |  |  |  |  |  |  | cg26440142 | HLX | 1.56E-05 |  |  |  |  |  | 1 |  |  | 1 |  |  |
| cg27401989 |  |  |  |  |  |  | cg27401989 | SP1 | 1.56E-05 |  |  |  |  |  | 1 |  |  | 1 |  |  |
| cg21571793 |  |  |  |  |  |  | cg21571793 | FOXN3 | 1.59E-05 |  |  |  |  |  | 1 |  |  | 1 |  |  |
| cg04167725 |  |  |  |  |  |  | cg04167725 | GEMIN8 | 1.59E-05 |  |  |  |  |  | 1 |  |  | 1 |  |  |
| cg19614811 |  |  |  |  |  |  | cg19614811 | GPR15 | 1.59E-05 |  |  |  |  |  | 1 |  |  | 1 |  |  |
| cg01111842 |  |  |  |  |  |  | cg01111842 | PNPLA7 | 1.59E-05 |  |  |  |  |  | 1 |  |  | 1 |  |  |
| cg09580249 |  |  |  |  |  |  | cg09580249 | RPH3AL | 1.59E-05 |  |  |  |  |  | 1 |  |  | 1 |  |  |
| cg09145256 |  |  |  |  |  |  | cg09145256 |  | 1.59E-05 |  |  |  |  |  | 1 |  |  | 1 |  |  |
| cg19925178 |  |  |  |  |  |  | cg19925178 |  | 1.59E-05 |  |  |  |  |  | 1 |  |  | 1 |  |  |
| cg09166536 |  |  |  |  |  |  | cg09166536 | COL9A3 | 1.60E-05 |  |  |  |  |  | 1 |  |  | 1 |  |  |
| cg08676730 |  |  |  |  |  |  | cg08676730 | PCTP | 1.60E-05 |  |  |  |  |  | 1 |  |  | 1 |  |  |
| cg08826460 |  |  |  |  |  |  | cg08826460 | LDLR | 1.63E-05 |  |  |  |  |  | 1 |  |  | 1 |  |  |
| cg27345534 |  |  |  |  |  |  | cg27345534 | PRB1 | 1.63E-05 |  |  |  |  |  | 1 |  |  | 1 |  |  |
| cg02917246 |  |  |  |  |  |  | cg02917246 | GSC2 | 1.64E-05 |  |  |  |  |  | 1 |  |  | 1 |  |  |
| cg14554244 |  |  |  |  |  |  | cg14554244 | NOTCH1 | 1.64E-05 |  |  |  |  |  | 1 |  |  | 1 |  |  |
| cg05274755 |  |  |  |  |  |  | cg05274755 | NPAS3 | 1.64E-05 |  |  |  |  |  | 1 |  |  | 1 |  |  |
| cg14404146 |  |  |  |  |  |  | cg14404146 | SLC24A2 | 1.64E-05 |  |  |  |  |  | 1 |  |  | 1 |  |  |
| cg03991106 |  |  |  |  |  |  | cg03991106 |  | 1.64E-05 |  |  |  |  |  | 1 |  |  | 1 |  |  |
| cg04195702 |  |  |  |  |  |  | cg04195702 |  | 1.64E-05 |  |  |  |  |  | 1 |  |  | 1 |  |  |
| cg06058681 |  |  |  |  |  |  | cg06058681 |  | 1.64E-05 |  |  |  |  |  | 1 |  |  | 1 |  |  |
| cg24315257 |  |  |  |  |  |  | cg24315257 |  | 1.64E-05 |  |  |  |  |  | 1 |  |  | 1 |  |  |
| cg27665449 |  |  |  |  |  |  | cg27665449 | ANKRD53 | 1.66E-05 |  |  |  |  |  | 1 |  |  | 1 |  |  |
| cg27258878 |  |  |  |  |  |  | cg27258878 | RBPMS | 1.66E-05 |  |  |  |  |  | 1 |  |  | 1 |  |  |
| cg19623519 |  |  |  |  |  |  | cg19623519 | C1orf229 | 1.67E-05 |  |  |  |  |  | 1 |  |  | 1 |  |  |
| cg04729173 |  |  |  |  |  |  | cg04729173 |  | 1.67E-05 |  |  |  |  |  | 1 |  |  | 1 |  |  |
| cg08556938 |  |  |  |  |  |  | cg08556938 |  | 1.67E-05 |  |  |  |  |  | 1 |  |  | 1 |  |  |
| cg09583024 |  |  |  |  |  |  | cg09583024 | RLTPR | 1.68E-05 |  |  |  |  |  | 1 |  |  | 1 |  |  |
| cg20806296 |  |  |  |  |  |  | cg20806296 |  | 1.70E-05 |  |  |  |  |  | 1 |  |  | 1 |  |  |
| cg03168749 |  |  |  |  |  |  | cg03168749 | OR8B12 | 1.71E-05 |  |  |  |  |  | 1 |  |  | 1 |  |  |
| cg14923379 |  |  |  |  |  |  | cg14923379 | FAM123B | 1.72E-05 |  |  |  |  |  | 1 |  |  | 1 |  |  |
| cg04294388 |  |  |  |  |  |  | cg04294388 |  | 1.72E-05 |  |  |  |  |  | 1 |  |  | 1 |  |  |
| cg26000722 |  |  |  |  |  |  | cg26000722 |  | 1.72E-05 |  |  |  |  |  | 1 |  |  | 1 |  |  |
| cg19117365 |  |  |  |  |  |  | cg19117365 | CLSTN2 | 1.73E-05 |  |  |  |  |  | 1 |  |  | 1 |  |  |
| cg05832051 |  |  |  |  |  |  | cg05832051 | MYADM | 1.73E-05 |  |  |  |  |  | 1 |  |  | 1 |  |  |
| cg04792227 |  |  |  |  |  |  | cg04792227 |  | 1.73E-05 |  |  |  |  |  | 1 |  |  | 1 |  |  |
| cg05905475 |  |  |  |  |  |  | cg05905475 |  | 1.73E-05 |  |  |  |  |  | 1 |  |  | 1 |  |  |
| cg15724941 |  |  |  |  |  |  | cg15724941 |  | 1.75E-05 |  |  |  |  |  | 1 |  |  | 1 |  |  |
| cg27248887 |  |  |  |  |  |  | cg27248887 | EEF1B2 | 1.76E-05 |  |  |  |  |  | 1 |  |  | 1 |  |  |
| cg16582889 |  |  |  |  |  |  | cg16582889 | GPT | 1.76E-05 |  |  |  |  |  | 1 |  |  | 1 |  |  |
| cg09985739 |  |  |  |  |  |  | cg09985739 | PCDH9 | 1.76E-05 |  |  |  |  |  | 1 |  |  | 1 |  |  |
| cg25051052 |  |  |  |  |  |  | cg25051052 | SPIRE1 | 1.76E-05 |  |  |  |  |  | 1 |  |  | 1 |  |  |
| cg25575845 |  |  |  |  |  |  | cg25575845 |  | 1.76E-05 |  |  |  |  |  | 1 |  |  | 1 |  |  |
| cg15894467 |  |  |  |  |  |  | cg15894467 | C1orf190 | 1.78E-05 |  |  |  |  |  | 1 |  |  | 1 |  |  |
| cg09843155 |  |  |  |  |  |  | cg09843155 |  | 1.78E-05 |  |  |  |  |  | 1 |  |  | 1 |  |  |
| cg14191885 |  |  |  |  |  |  | cg14191885 |  | 1.78E-05 |  |  |  |  |  | 1 |  |  | 1 |  |  |
| cg12584590 |  |  |  |  |  |  | cg12584590 | TMEM233 | 1.80E-05 |  |  |  |  |  | 1 |  |  | 1 |  |  |
| cg18148726 |  |  |  |  |  |  | cg18148726 |  | 1.80E-05 |  |  |  |  |  | 1 |  |  | 1 |  |  |
| cg19157327 |  |  |  |  |  |  | cg19157327 |  | 1.81E-05 |  |  |  |  |  | 1 |  |  | 1 |  |  |
| cg08914271 |  |  |  |  |  |  | cg08914271 |  | 1.82E-05 |  |  |  |  |  | 1 |  |  | 1 |  |  |
| cg14257543 |  |  |  |  |  |  | cg14257543 |  | 1.82E-05 |  |  |  |  |  | 1 |  |  | 1 |  |  |
| cg01810593 |  |  |  |  |  |  | cg01810593 | KIAA1967 | 1.83E-05 |  |  |  |  |  | 1 |  |  | 1 |  |  |
| cg26872588 |  |  |  |  |  |  | cg26872588 | C1orf198 | 1.85E-05 |  |  |  |  |  | 1 |  |  | 1 |  |  |
| cg12058781 |  |  |  |  |  |  | cg12058781 | GBP4 | 1.85E-05 |  |  |  |  |  | 1 |  |  | 1 |  |  |
| cg00831247 |  |  |  |  |  |  | cg00831247 | LOXL3 | 1.85E-05 |  |  |  |  |  | 1 |  |  | 1 |  |  |
| cg06968752 |  |  |  |  |  |  | cg06968752 |  | 1.85E-05 |  |  |  |  |  | 1 |  |  | 1 |  |  |
| cg10502231 |  |  |  |  |  |  | cg10502231 |  | 1.85E-05 |  |  |  |  |  | 1 |  |  | 1 |  |  |
| cg22466850 |  |  |  |  |  |  | cg22466850 |  | 1.85E-05 |  |  |  |  |  | 1 |  |  | 1 |  |  |
| cg03881711 |  |  |  |  |  |  | cg03881711 |  | 1.86E-05 |  |  |  |  |  | 1 |  |  | 1 |  |  |
| cg14595275 |  |  |  |  |  |  | cg14595275 | C17orf72 | 1.91E-05 |  |  |  |  |  | 1 |  |  | 1 |  |  |
| cg00124488 |  |  |  |  |  |  | cg00124488 | BAT1 | 1.92E-05 |  |  |  |  |  | 1 |  |  | 1 |  |  |
| cg02724903 |  |  |  |  |  |  | cg02724903 | TFDP1 | 1.92E-05 |  |  |  |  |  | 1 |  |  | 1 |  |  |
| cg04573872 |  |  |  |  |  |  | cg04573872 | CATSPER4 | 1.93E-05 |  |  |  |  |  | 1 |  |  | 1 |  |  |
| cg19189355 |  |  |  |  |  |  | cg19189355 | DLL1 | 1.93E-05 |  |  |  |  |  | 1 |  |  | 1 |  |  |
| cg14242958 |  |  |  |  |  |  | cg14242958 | KIAA1377 | 1.93E-05 |  |  |  |  |  | 1 |  |  | 1 |  |  |
| cg16226300 |  |  |  |  |  |  | cg16226300 | RPS12 | 1.93E-05 |  |  |  |  |  | 1 |  |  | 1 |  |  |
| cg22852353 |  |  |  |  |  |  | cg22852353 | LMX1A | 1.96E-05 |  |  |  |  |  | 1 |  |  | 1 |  |  |
| cg02788938 |  |  |  |  |  |  | cg02788938 |  | 1.96E-05 |  |  |  |  |  | 1 |  |  | 1 |  |  |
| cg22092397 |  |  |  |  |  |  | cg22092397 |  | 1.96E-05 |  |  |  |  |  | 1 |  |  | 1 |  |  |
| cg12867237 |  |  |  |  |  |  | cg12867237 | ACCS | 1.97E-05 |  |  |  |  |  | 1 |  |  | 1 |  |  |
| cg14582009 |  |  |  |  |  |  | cg14582009 | GLTSCR1 | 1.97E-05 |  |  |  |  |  | 1 |  |  | 1 |  |  |
| cg00421612 |  |  |  |  |  |  | cg00421612 |  | 1.97E-05 |  |  |  |  |  | 1 |  |  | 1 |  |  |
| cg01810416 |  |  |  |  |  |  | cg01810416 |  | 1.97E-05 |  |  |  |  |  | 1 |  |  | 1 |  |  |
| cg18123072 |  |  |  |  |  |  | cg18123072 | CSNK1G3 | 2.00E-05 |  |  |  |  |  | 1 |  |  | 1 |  |  |
| cg05388492 |  |  |  |  |  |  | cg05388492 | ESRRG | 2.00E-05 |  |  |  |  |  | 1 |  |  | 1 |  |  |
| cg01094121 |  |  |  |  |  |  | cg01094121 |  | 2.00E-05 |  |  |  |  |  | 1 |  |  | 1 |  |  |
| cg13750180 |  |  |  |  |  |  | cg13750180 | ZNF815 | 2.01E-05 |  |  |  |  |  | 1 |  |  | 1 |  |  |
| cg00911962 |  |  |  |  |  |  | cg00911962 | 42253 | 2.03E-05 |  |  |  |  |  | 1 |  |  | 1 |  |  |
| cg12920393 |  |  |  |  |  |  | cg12920393 | HIST1H2BK | 2.04E-05 |  |  |  |  |  | 1 |  |  | 1 |  |  |
| cg00282216 |  |  |  |  |  |  | cg00282216 | KIAA1688 | 2.04E-05 |  |  |  |  |  | 1 |  |  | 1 |  |  |
| cg08583001 |  |  |  |  |  |  | cg08583001 | PVRL2 | 2.04E-05 |  |  |  |  |  | 1 |  |  | 1 |  |  |
| cg16475705 |  |  |  |  |  |  | cg16475705 | UNC5C | 2.04E-05 |  |  |  |  |  | 1 |  |  | 1 |  |  |
| cg04717613 |  |  |  |  |  |  | cg04717613 |  | 2.04E-05 |  |  |  |  |  | 1 |  |  | 1 |  |  |
| cg12340381 |  |  |  |  |  |  | cg12340381 |  | 2.04E-05 |  |  |  |  |  | 1 |  |  | 1 |  |  |
| cg18998938 |  |  |  |  |  |  | cg18998938 |  | 2.04E-05 |  |  |  |  |  | 1 |  |  | 1 |  |  |
| cg26487629 |  |  |  |  |  |  | cg26487629 |  | 2.04E-05 |  |  |  |  |  | 1 |  |  | 1 |  |  |
| cg27060240 |  |  |  |  |  |  | cg27060240 |  | 2.04E-05 |  |  |  |  |  | 1 |  |  | 1 |  |  |
| cg03502236 |  |  |  |  |  |  | cg03502236 | MUC2 | 2.08E-05 |  |  |  |  |  | 1 |  |  | 1 |  |  |
| cg19069039 |  |  |  |  |  |  | cg19069039 |  | 2.08E-05 |  |  |  |  |  | 1 |  |  | 1 |  |  |
| cg22397910 |  |  |  |  |  |  | cg22397910 | CPSF3L | 2.09E-05 |  |  |  |  |  | 1 |  |  | 1 |  |  |
| cg19062112 |  |  |  |  |  |  | cg19062112 | RCOR1 | 2.09E-05 |  |  |  |  |  | 1 |  |  | 1 |  |  |
| cg16602460 |  |  |  |  |  |  | cg16602460 | PBX2 | 2.10E-05 |  |  |  |  |  | 1 |  |  | 1 |  |  |
| cg21860285 |  |  |  |  |  |  | cg21860285 | CPA6 | 2.11E-05 |  |  |  |  |  | 1 |  |  | 1 |  |  |
| cg10888878 |  |  |  |  |  |  | cg10888878 |  | 2.11E-05 |  |  |  |  |  | 1 |  |  | 1 |  |  |
| cg07675184 |  |  |  |  |  |  | cg07675184 | EMX2OS | 2.12E-05 |  |  |  |  |  | 1 |  |  | 1 |  |  |
| cg10192196 |  |  |  |  |  |  | cg10192196 | PSMB8 | 2.12E-05 |  |  |  |  |  | 1 |  |  | 1 |  |  |
| cg20805479 |  |  |  |  |  |  | cg20805479 | CLK1 | 2.15E-05 |  |  |  |  |  | 1 |  |  | 1 |  |  |
| cg05435065 |  |  |  |  |  |  | cg05435065 | POR | 2.15E-05 |  |  |  |  |  | 1 |  |  | 1 |  |  |
| cg16314263 |  |  |  |  |  |  | cg16314263 | A2M | 2.17E-05 |  |  |  |  |  | 1 |  |  | 1 |  |  |
| cg21557724 |  |  |  |  |  |  | cg21557724 | TSSC1 | 2.17E-05 |  |  |  |  |  | 1 |  |  | 1 |  |  |
| cg04880611 |  |  |  |  |  |  | cg04880611 | MIR548H4 | 2.18E-05 |  |  |  |  |  | 1 |  |  | 1 |  |  |
| cg06872036 |  |  |  |  |  |  | cg06872036 | RASSF5 | 2.18E-05 |  |  |  |  |  | 1 |  |  | 1 |  |  |
| cg04636881 |  |  |  |  |  |  | cg04636881 |  | 2.18E-05 |  |  |  |  |  | 1 |  |  | 1 |  |  |
| cg12989650 |  |  |  |  |  |  | cg12989650 | ARHGEF15 | 2.19E-05 |  |  |  |  |  | 1 |  |  | 1 |  |  |
| cg06381350 |  |  |  |  |  |  | cg06381350 | IRGC | 2.19E-05 |  |  |  |  |  | 1 |  |  | 1 |  |  |
| cg26433208 |  |  |  |  |  |  | cg26433208 | CTPS | 2.20E-05 |  |  |  |  |  | 1 |  |  | 1 |  |  |
| cg03792491 |  |  |  |  |  |  | cg03792491 | GPR120 | 2.20E-05 |  |  |  |  |  | 1 |  |  | 1 |  |  |
| cg11692477 |  |  |  |  |  |  | cg11692477 | SLC40A1 | 2.20E-05 |  |  |  |  |  | 1 |  |  | 1 |  |  |
| cg04433306 |  |  |  |  |  |  | cg04433306 | UBE2H | 2.20E-05 |  |  |  |  |  | 1 |  |  | 1 |  |  |
| cg08076532 |  |  |  |  |  |  | cg08076532 |  | 2.20E-05 |  |  |  |  |  | 1 |  |  | 1 |  |  |
| cg21694626 |  |  |  |  |  |  | cg21694626 |  | 2.20E-05 |  |  |  |  |  | 1 |  |  | 1 |  |  |
| cg25842470 |  |  |  |  |  |  | cg25842470 |  | 2.21E-05 |  |  |  |  |  | 1 |  |  | 1 |  |  |
| cg07867325 |  |  |  |  |  |  | cg07867325 |  | 2.23E-05 |  |  |  |  |  | 1 |  |  | 1 |  |  |
| cg19212828 |  |  |  |  |  |  | cg19212828 |  | 2.23E-05 |  |  |  |  |  | 1 |  |  | 1 |  |  |
| cg00828709 |  |  |  |  |  |  | cg00828709 | C10orf71 | 2.25E-05 |  |  |  |  |  | 1 |  |  | 1 |  |  |
| cg18865733 |  |  |  |  |  |  | cg18865733 | DEFB125 | 2.25E-05 |  |  |  |  |  | 1 |  |  | 1 |  |  |
| cg09333325 |  |  |  |  |  |  | cg09333325 | KIF26A | 2.25E-05 |  |  |  |  |  | 1 |  |  | 1 |  |  |
| cg21350392 |  |  |  |  |  |  | cg21350392 | MTUS2 | 2.25E-05 |  |  |  |  |  | 1 |  |  | 1 |  |  |
| cg20529334 |  |  |  |  |  |  | cg20529334 | SLCO1A2 | 2.25E-05 |  |  |  |  |  | 1 |  |  | 1 |  |  |
| cg24162465 |  |  |  |  |  |  | cg24162465 |  | 2.25E-05 |  |  |  |  |  | 1 |  |  | 1 |  |  |
| cg00950473 |  |  |  |  |  |  | cg00950473 | APCDD1L | 2.26E-05 |  |  |  |  |  | 1 |  |  | 1 |  |  |
| cg27589809 |  |  |  |  |  |  | cg27589809 | CISH | 2.26E-05 |  |  |  |  |  | 1 |  |  | 1 |  |  |
| cg14313916 |  |  |  |  |  |  | cg14313916 |  | 2.26E-05 |  |  |  |  |  | 1 |  |  | 1 |  |  |
| cg17114402 |  |  |  |  |  |  | cg17114402 | ANKRD36 | 2.27E-05 |  |  |  |  |  | 1 |  |  | 1 |  |  |
| cg20072001 |  |  |  |  |  |  | cg20072001 | ASB7 | 2.27E-05 |  |  |  |  |  | 1 |  |  | 1 |  |  |
| cg08402107 |  |  |  |  |  |  | cg08402107 | GSTO2 | 2.27E-05 |  |  |  |  |  | 1 |  |  | 1 |  |  |
| cg03027241 |  |  |  |  |  |  | cg03027241 | KCNG1 | 2.27E-05 |  |  |  |  |  | 1 |  |  | 1 |  |  |
| cg07078532 |  |  |  |  |  |  | cg07078532 | SLC26A11 | 2.27E-05 |  |  |  |  |  | 1 |  |  | 1 |  |  |
| cg12617538 |  |  |  |  |  |  | cg12617538 |  | 2.27E-05 |  |  |  |  |  | 1 |  |  | 1 |  |  |
| cg17063731 |  |  |  |  |  |  | cg17063731 |  | 2.27E-05 |  |  |  |  |  | 1 |  |  | 1 |  |  |
| cg24651790 |  |  |  |  |  |  | cg24651790 |  | 2.27E-05 |  |  |  |  |  | 1 |  |  | 1 |  |  |
| cg06249109 |  |  |  |  |  |  | cg06249109 |  | 2.28E-05 |  |  |  |  |  | 1 |  |  | 1 |  |  |
| cg07468782 |  |  |  |  |  |  | cg07468782 | ZNF232 | 2.29E-05 |  |  |  |  |  | 1 |  |  | 1 |  |  |
| cg10703101 |  |  |  |  |  |  | cg10703101 | AMAC1L3 | 2.30E-05 |  |  |  |  |  | 1 |  |  | 1 |  |  |
| cg05586134 |  |  |  |  |  |  | cg05586134 | PTCRA | 2.30E-05 |  |  |  |  |  | 1 |  |  | 1 |  |  |
| cg05740739 |  |  |  |  |  |  | cg05740739 | OR6B3 | 2.31E-05 |  |  |  |  |  | 1 |  |  | 1 |  |  |
| cg17657594 |  |  |  |  |  |  | cg17657594 | TRIM27 | 2.31E-05 |  |  |  |  |  | 1 |  |  | 1 |  |  |
| cg27379715 |  |  |  |  |  |  | cg27379715 |  | 2.34E-05 |  |  |  |  |  | 1 |  |  | 1 |  |  |
| cg02882813 |  |  |  |  |  |  | cg02882813 | CST5 | 2.36E-05 |  |  |  |  |  | 1 |  |  | 1 |  |  |
| cg21599794 |  |  |  |  |  |  | cg21599794 | XKR9 | 2.36E-05 |  |  |  |  |  | 1 |  |  | 1 |  |  |
| cg04425458 |  |  |  |  |  |  | cg04425458 | KCNQ3 | 2.39E-05 |  |  |  |  |  | 1 |  |  | 1 |  |  |
| cg25015290 |  |  |  |  |  |  | cg25015290 | DCLRE1C | 2.40E-05 |  |  |  |  |  | 1 |  |  | 1 |  |  |
| cg02776750 |  |  |  |  |  |  | cg02776750 | C12orf42 | 2.41E-05 |  |  |  |  |  | 1 |  |  | 1 |  |  |
| cg26544188 |  |  |  |  |  |  | cg26544188 | SNX29 | 2.41E-05 |  |  |  |  |  | 1 |  |  | 1 |  |  |
| cg05938628 |  |  |  |  |  |  | cg05938628 | MYO10 | 2.43E-05 |  |  |  |  |  | 1 |  |  | 1 |  |  |
| cg20992503 |  |  |  |  |  |  | cg20992503 | NACA2 | 2.43E-05 |  |  |  |  |  | 1 |  |  | 1 |  |  |
| cg16168311 |  |  |  |  |  |  | cg16168311 | APOA1BP | 2.44E-05 |  |  |  |  |  | 1 |  |  | 1 |  |  |
| cg16775460 |  |  |  |  |  |  | cg16775460 | TBC1D16 | 2.44E-05 |  |  |  |  |  | 1 |  |  | 1 |  |  |
| cg25910466 |  |  |  |  |  |  | cg25910466 | XRN2 | 2.45E-05 |  |  |  |  |  | 1 |  |  | 1 |  |  |
| cg18233942 |  |  |  |  |  |  | cg18233942 | C1orf53 | 2.46E-05 |  |  |  |  |  | 1 |  |  | 1 |  |  |
| cg21646084 |  |  |  |  |  |  | cg21646084 | FAM49A | 2.46E-05 |  |  |  |  |  | 1 |  |  | 1 |  |  |
| cg15756928 |  |  |  |  |  |  | cg15756928 | GFRAL | 2.46E-05 |  |  |  |  |  | 1 |  |  | 1 |  |  |
| cg25867545 |  |  |  |  |  |  | cg25867545 | LAMP3 | 2.46E-05 |  |  |  |  |  | 1 |  |  | 1 |  |  |
| cg06897120 |  |  |  |  |  |  | cg06897120 | LOC342346 | 2.46E-05 |  |  |  |  |  | 1 |  |  | 1 |  |  |
| cg09249637 |  |  |  |  |  |  | cg09249637 | PQLC3 | 2.46E-05 |  |  |  |  |  | 1 |  |  | 1 |  |  |
| cg23629792 |  |  |  |  |  |  | cg23629792 | PRKCZ | 2.46E-05 |  |  |  |  |  | 1 |  |  | 1 |  |  |
| cg22091132 |  |  |  |  |  |  | cg22091132 | SEC11C | 2.46E-05 |  |  |  |  |  | 1 |  |  | 1 |  |  |
| cg01323766 |  |  |  |  |  |  | cg01323766 |  | 2.46E-05 |  |  |  |  |  | 1 |  |  | 1 |  |  |
| cg18396789 |  |  |  |  |  |  | cg18396789 |  | 2.46E-05 |  |  |  |  |  | 1 |  |  | 1 |  |  |
| cg08708209 |  |  |  |  |  |  | cg08708209 | DUSP10 | 2.47E-05 |  |  |  |  |  | 1 |  |  | 1 |  |  |
| cg14768782 |  |  |  |  |  |  | cg14768782 | KIAA1257 | 2.47E-05 |  |  |  |  |  | 1 |  |  | 1 |  |  |
| cg27167195 |  |  |  |  |  |  | cg27167195 | PKP4 | 2.47E-05 |  |  |  |  |  | 1 |  |  | 1 |  |  |
| cg15267250 |  |  |  |  |  |  | cg15267250 |  | 2.47E-05 |  |  |  |  |  | 1 |  |  | 1 |  |  |
| cg17451760 |  |  |  |  |  |  | cg17451760 | LTBP3 | 2.48E-05 |  |  |  |  |  | 1 |  |  | 1 |  |  |
| cg09916030 |  |  |  |  |  |  | cg09916030 | CLDN14 | 2.52E-05 |  |  |  |  |  | 1 |  |  | 1 |  |  |
| cg14840863 |  |  |  |  |  |  | cg14840863 | COLEC11 | 2.52E-05 |  |  |  |  |  | 1 |  |  | 1 |  |  |
| cg15959715 |  |  |  |  |  |  | cg15959715 | GAD2 | 2.52E-05 |  |  |  |  |  | 1 |  |  | 1 |  |  |
| cg16098304 |  |  |  |  |  |  | cg16098304 | GAL3ST2 | 2.52E-05 |  |  |  |  |  | 1 |  |  | 1 |  |  |
| cg18985133 |  |  |  |  |  |  | cg18985133 | IDO2 | 2.52E-05 |  |  |  |  |  | 1 |  |  | 1 |  |  |
| cg11779204 |  |  |  |  |  |  | cg11779204 | CTNND2 | 2.53E-05 |  |  |  |  |  | 1 |  |  | 1 |  |  |
| cg01720945 |  |  |  |  |  |  | cg01720945 | PLEKHA6 | 2.55E-05 |  |  |  |  |  | 1 |  |  | 1 |  |  |
| cg24070837 |  |  |  |  |  |  | cg24070837 | ZNF382 | 2.57E-05 |  |  |  |  |  | 1 |  |  | 1 |  |  |
| cg04758185 |  |  |  |  |  |  | cg04758185 | PLK1 | 2.59E-05 |  |  |  |  |  | 1 |  |  | 1 |  |  |
| cg09542690 |  |  |  |  |  |  | cg09542690 | ZNF445 | 2.59E-05 |  |  |  |  |  | 1 |  |  | 1 |  |  |
| cg18334819 |  |  |  |  |  |  | cg18334819 |  | 2.59E-05 |  |  |  |  |  | 1 |  |  | 1 |  |  |
| cg22010909 |  |  |  |  |  |  | cg22010909 | ATP11A | 2.60E-05 |  |  |  |  |  | 1 |  |  | 1 |  |  |
| cg25457109 |  |  |  |  |  |  | cg25457109 |  | 2.60E-05 |  |  |  |  |  | 1 |  |  | 1 |  |  |
| cg19023320 |  |  |  |  |  |  | cg19023320 | IQSEC1 | 2.61E-05 |  |  |  |  |  | 1 |  |  | 1 |  |  |
| cg27110491 |  |  |  |  |  |  | cg27110491 | UPP1 | 2.61E-05 |  |  |  |  |  | 1 |  |  | 1 |  |  |
| cg09106556 |  |  |  |  |  |  | cg09106556 | ZNF302 | 2.61E-05 |  |  |  |  |  | 1 |  |  | 1 |  |  |
| cg02406531 |  |  |  |  |  |  | cg02406531 |  | 2.61E-05 |  |  |  |  |  | 1 |  |  | 1 |  |  |
| cg07842327 |  |  |  |  |  |  | cg07842327 | SKI | 2.62E-05 |  |  |  |  |  | 1 |  |  | 1 |  |  |
| cg02351018 |  |  |  |  |  |  | cg02351018 |  | 2.62E-05 |  |  |  |  |  | 1 |  |  | 1 |  |  |
| cg19515468 |  |  |  |  |  |  | cg19515468 |  | 2.62E-05 |  |  |  |  |  | 1 |  |  | 1 |  |  |
| cg21217129 |  |  |  |  |  |  | cg21217129 | ADARB1 | 2.63E-05 |  |  |  |  |  | 1 |  |  | 1 |  |  |
| cg24377560 |  |  |  |  |  |  | cg24377560 | AFAP1 | 2.63E-05 |  |  |  |  |  | 1 |  |  | 1 |  |  |
| cg05112617 |  |  |  |  |  |  | cg05112617 | ARL13B | 2.63E-05 |  |  |  |  |  | 1 |  |  | 1 |  |  |
| cg19910568 |  |  |  |  |  |  | cg19910568 | DNAJC1 | 2.63E-05 |  |  |  |  |  | 1 |  |  | 1 |  |  |
| cg10583473 |  |  |  |  |  |  | cg10583473 | MICALL2 | 2.63E-05 |  |  |  |  |  | 1 |  |  | 1 |  |  |
| cg15777910 |  |  |  |  |  |  | cg15777910 | TRPV1 | 2.63E-05 |  |  |  |  |  | 1 |  |  | 1 |  |  |
| cg08257600 |  |  |  |  |  |  | cg08257600 | ADCK4 | 2.64E-05 |  |  |  |  |  | 1 |  |  | 1 |  |  |
| cg06648782 |  |  |  |  |  |  | cg06648782 | PACRG | 2.64E-05 |  |  |  |  |  | 1 |  |  | 1 |  |  |
| cg20407796 |  |  |  |  |  |  | cg20407796 | B3GNTL1 | 2.65E-05 |  |  |  |  |  | 1 |  |  | 1 |  |  |
| cg03065888 |  |  |  |  |  |  | cg03065888 | CYC1 | 2.68E-05 |  |  |  |  |  | 1 |  |  | 1 |  |  |
| cg03421069 |  |  |  |  |  |  | cg03421069 | PAN3 | 2.68E-05 |  |  |  |  |  | 1 |  |  | 1 |  |  |
| cg07387813 |  |  |  |  |  |  | cg07387813 | RPL14 | 2.68E-05 |  |  |  |  |  | 1 |  |  | 1 |  |  |
| cg24807761 |  |  |  |  |  |  | cg24807761 | CALD1 | 2.73E-05 |  |  |  |  |  | 1 |  |  | 1 |  |  |
| cg18040892 |  |  |  |  |  |  | cg18040892 | SMARCA4 | 2.73E-05 |  |  |  |  |  | 1 |  |  | 1 |  |  |
| cg08888410 |  |  |  |  |  |  | cg08888410 | ZSCAN10 | 2.73E-05 |  |  |  |  |  | 1 |  |  | 1 |  |  |
| cg24263998 |  |  |  |  |  |  | cg24263998 |  | 2.73E-05 |  |  |  |  |  | 1 |  |  | 1 |  |  |
| cg01604210 |  |  |  |  |  |  | cg01604210 | HMGCL | 2.75E-05 |  |  |  |  |  | 1 |  |  | 1 |  |  |
| cg12742432 |  |  |  |  |  |  | cg12742432 | SYTL1 | 2.75E-05 |  |  |  |  |  | 1 |  |  | 1 |  |  |
| cg10502232 |  |  |  |  |  |  | cg10502232 | ATP6V0C | 2.76E-05 |  |  |  |  |  | 1 |  |  | 1 |  |  |
| cg00498419 |  |  |  |  |  |  | cg00498419 | MKI67 | 2.76E-05 |  |  |  |  |  | 1 |  |  | 1 |  |  |
| cg09180820 |  |  |  |  |  |  | cg09180820 | PRKCZ | 2.76E-05 |  |  |  |  |  | 1 |  |  | 1 |  |  |
| cg11870561 |  |  |  |  |  |  | cg11870561 |  | 2.76E-05 |  |  |  |  |  | 1 |  |  | 1 |  |  |
| cg19615731 |  |  |  |  |  |  | cg19615731 | DIDO1 | 2.78E-05 |  |  |  |  |  | 1 |  |  | 1 |  |  |
| cg14303616 |  |  |  |  |  |  | cg14303616 | RTP1 | 2.78E-05 |  |  |  |  |  | 1 |  |  | 1 |  |  |
| cg07437919 |  |  |  |  |  |  | cg07437919 | SLC45A4 | 2.78E-05 |  |  |  |  |  | 1 |  |  | 1 |  |  |
| cg14065224 |  |  |  |  |  |  | cg14065224 | VWA3B | 2.79E-05 |  |  |  |  |  | 1 |  |  | 1 |  |  |
| cg20758953 |  |  |  |  |  |  | cg20758953 | ZNF74 | 2.81E-05 |  |  |  |  |  | 1 |  |  | 1 |  |  |
| cg18205465 |  |  |  |  |  |  | cg18205465 | C7orf40 | 2.82E-05 |  |  |  |  |  | 1 |  |  | 1 |  |  |
| cg02618319 |  |  |  |  |  |  | cg02618319 | USP2 | 2.83E-05 |  |  |  |  |  | 1 |  |  | 1 |  |  |
| cg22403782 |  |  |  |  |  |  | cg22403782 | ALPP | 2.84E-05 |  |  |  |  |  | 1 |  |  | 1 |  |  |
| cg18766608 |  |  |  |  |  |  | cg18766608 | DAGLA | 2.84E-05 |  |  |  |  |  | 1 |  |  | 1 |  |  |
| cg18075287 |  |  |  |  |  |  | cg18075287 | GABARAPL1 | 2.84E-05 |  |  |  |  |  | 1 |  |  | 1 |  |  |
| cg14312334 |  |  |  |  |  |  | cg14312334 | CIT | 2.86E-05 |  |  |  |  |  | 1 |  |  | 1 |  |  |
| cg07153264 |  |  |  |  |  |  | cg07153264 | FBXL19 | 2.86E-05 |  |  |  |  |  | 1 |  |  | 1 |  |  |
| cg18118795 |  |  |  |  |  |  | cg18118795 | RBBP4 | 2.86E-05 |  |  |  |  |  | 1 |  |  | 1 |  |  |
| cg04908325 |  |  |  |  |  |  | cg04908325 | ZNF410 | 2.86E-05 |  |  |  |  |  | 1 |  |  | 1 |  |  |
| cg05985501 |  |  |  |  |  |  | cg05985501 |  | 2.87E-05 |  |  |  |  |  | 1 |  |  | 1 |  |  |
| cg13324539 |  |  |  |  |  |  | cg13324539 |  | 2.87E-05 |  |  |  |  |  | 1 |  |  | 1 |  |  |
| cg18499636 |  |  |  |  |  |  | cg18499636 | ARHGEF10 | 2.91E-05 |  |  |  |  |  | 1 |  |  | 1 |  |  |
| cg08924554 |  |  |  |  |  |  | cg08924554 | IGFBP7 | 2.93E-05 |  |  |  |  |  | 1 |  |  | 1 |  |  |
| cg09299076 |  |  |  |  |  |  | cg09299076 | ZNF276 | 2.93E-05 |  |  |  |  |  | 1 |  |  | 1 |  |  |
| cg10326891 |  |  |  |  |  |  | cg10326891 | NUDT9 | 2.94E-05 |  |  |  |  |  | 1 |  |  | 1 |  |  |
| cg07855322 |  |  |  |  |  |  | cg07855322 |  | 2.94E-05 |  |  |  |  |  | 1 |  |  | 1 |  |  |
| cg06361278 |  |  |  |  |  |  | cg06361278 |  | 3.04E-05 |  |  |  |  |  | 1 |  |  | 1 |  |  |
| cg06658468 |  |  |  |  |  |  | cg06658468 | OTOP1 | 3.06E-05 |  |  |  |  |  | 1 |  |  | 1 |  |  |
| cg06616029 |  |  |  |  |  |  | cg06616029 | TTBK2 | 3.08E-05 |  |  |  |  |  | 1 |  |  | 1 |  |  |
| cg07804408 |  |  |  |  |  |  | cg07804408 |  | 3.08E-05 |  |  |  |  |  | 1 |  |  | 1 |  |  |
| cg07104380 |  |  |  |  |  |  | cg07104380 |  | 3.09E-05 |  |  |  |  |  | 1 |  |  | 1 |  |  |
| cg07495755 |  |  |  |  |  |  | cg07495755 |  | 3.11E-05 |  |  |  |  |  | 1 |  |  | 1 |  |  |
| cg20445034 |  |  |  |  |  |  | cg20445034 | KCNH4 | 3.14E-05 |  |  |  |  |  | 1 |  |  | 1 |  |  |
| cg14487577 |  |  |  |  |  |  | cg14487577 | MIR133B | 3.14E-05 |  |  |  |  |  | 1 |  |  | 1 |  |  |
| cg14606129 |  |  |  |  |  |  | cg14606129 |  | 3.14E-05 |  |  |  |  |  | 1 |  |  | 1 |  |  |
| cg05066621 |  |  |  |  |  |  | cg05066621 | RBP3 | 3.15E-05 |  |  |  |  |  | 1 |  |  | 1 |  |  |
| cg01419670 |  |  |  |  |  |  | cg01419670 |  | 3.16E-05 |  |  |  |  |  | 1 |  |  | 1 |  |  |
| cg14681176 |  |  |  |  |  |  | cg14681176 | SIRT7 | 3.17E-05 |  |  |  |  |  | 1 |  |  | 1 |  |  |
| cg19165344 |  |  |  |  |  |  | cg19165344 | AP1B1 | 3.18E-05 |  |  |  |  |  | 1 |  |  | 1 |  |  |
| cg11648740 |  |  |  |  |  |  | cg11648740 | FLJ35220 | 3.19E-05 |  |  |  |  |  | 1 |  |  | 1 |  |  |
| cg00887547 |  |  |  |  |  |  | cg00887547 | TAOK3 | 3.19E-05 |  |  |  |  |  | 1 |  |  | 1 |  |  |
| cg24865495 |  |  |  |  |  |  | cg24865495 | MACROD1 | 3.20E-05 |  |  |  |  |  | 1 |  |  | 1 |  |  |
| cg13642800 |  |  |  |  |  |  | cg13642800 |  | 3.20E-05 |  |  |  |  |  | 1 |  |  | 1 |  |  |
| cg18206858 |  |  |  |  |  |  | cg18206858 |  | 3.20E-05 |  |  |  |  |  | 1 |  |  | 1 |  |  |
| cg23463205 |  |  |  |  |  |  | cg23463205 | PNLIP | 3.23E-05 |  |  |  |  |  | 1 |  |  | 1 |  |  |
| cg05192538 |  |  |  |  |  |  | cg05192538 |  | 3.23E-05 |  |  |  |  |  | 1 |  |  | 1 |  |  |
| cg07141622 |  |  |  |  |  |  | cg07141622 |  | 3.23E-05 |  |  |  |  |  | 1 |  |  | 1 |  |  |
| cg19134728 |  |  |  |  |  |  | cg19134728 | JAKMIP3 | 3.24E-05 |  |  |  |  |  | 1 |  |  | 1 |  |  |
| cg20665002 |  |  |  |  |  |  | cg20665002 | ATP8A2 | 3.28E-05 |  |  |  |  |  | 1 |  |  | 1 |  |  |
| cg09934692 |  |  |  |  |  |  | cg09934692 | FIGNL2 | 3.28E-05 |  |  |  |  |  | 1 |  |  | 1 |  |  |
| cg06866416 |  |  |  |  |  |  | cg06866416 | LOC595101 | 3.28E-05 |  |  |  |  |  | 1 |  |  | 1 |  |  |
| cg12661610 |  |  |  |  |  |  | cg12661610 | RGS12 | 3.28E-05 |  |  |  |  |  | 1 |  |  | 1 |  |  |
| cg17448109 |  |  |  |  |  |  | cg17448109 | RNF115 | 3.28E-05 |  |  |  |  |  | 1 |  |  | 1 |  |  |
| cg23170535 |  |  |  |  |  |  | cg23170535 |  | 3.28E-05 |  |  |  |  |  | 1 |  |  | 1 |  |  |
| cg02423267 |  |  |  |  |  |  | cg02423267 |  | 3.29E-05 |  |  |  |  |  | 1 |  |  | 1 |  |  |
| cg13532410 |  |  |  |  |  |  | cg13532410 | CACNA2D3 | 3.31E-05 |  |  |  |  |  | 1 |  |  | 1 |  |  |
| cg23208326 |  |  |  |  |  |  | cg23208326 | MTHFD2L | 3.32E-05 |  |  |  |  |  | 1 |  |  | 1 |  |  |
| cg23243012 |  |  |  |  |  |  | cg23243012 | PROKR1 | 3.36E-05 |  |  |  |  |  | 1 |  |  | 1 |  |  |
| cg04400496 |  |  |  |  |  |  | cg04400496 |  | 3.40E-05 |  |  |  |  |  | 1 |  |  | 1 |  |  |
| cg10542127 |  |  |  |  |  |  | cg10542127 |  | 3.42E-05 |  |  |  |  |  | 1 |  |  | 1 |  |  |
| cg21889116 |  |  |  |  |  |  | cg21889116 | TMEM68 | 3.44E-05 |  |  |  |  |  | 1 |  |  | 1 |  |  |
| cg01739965 |  |  |  |  |  |  | cg01739965 | VPS54 | 3.45E-05 |  |  |  |  |  | 1 |  |  | 1 |  |  |
| cg19435453 |  |  |  |  |  |  | cg19435453 |  | 3.45E-05 |  |  |  |  |  | 1 |  |  | 1 |  |  |
| cg27551895 |  |  |  |  |  |  | cg27551895 |  | 3.46E-05 |  |  |  |  |  | 1 |  |  | 1 |  |  |
| cg02340915 |  |  |  |  |  |  | cg02340915 | KDM2B | 3.47E-05 |  |  |  |  |  | 1 |  |  | 1 |  |  |
| cg14412794 |  |  |  |  |  |  | cg14412794 | WWC2 | 3.47E-05 |  |  |  |  |  | 1 |  |  | 1 |  |  |
| cg12219123 |  |  |  |  |  |  | cg12219123 | AKR7A2 | 3.48E-05 |  |  |  |  |  | 1 |  |  | 1 |  |  |
| cg20330023 |  |  |  |  |  |  | cg20330023 | ARNT2 | 3.48E-05 |  |  |  |  |  | 1 |  |  | 1 |  |  |
| cg14153876 |  |  |  |  |  |  | cg14153876 | GPD2 | 3.48E-05 |  |  |  |  |  | 1 |  |  | 1 |  |  |
| cg13424330 |  |  |  |  |  |  | cg13424330 | HAUS8 | 3.48E-05 |  |  |  |  |  | 1 |  |  | 1 |  |  |
| cg04951822 |  |  |  |  |  |  | cg04951822 | OAS1 | 3.48E-05 |  |  |  |  |  | 1 |  |  | 1 |  |  |
| cg14860917 |  |  |  |  |  |  | cg14860917 | PSD3 | 3.49E-05 |  |  |  |  |  | 1 |  |  | 1 |  |  |
| cg18748085 |  |  |  |  |  |  | cg18748085 | CDH12 | 3.50E-05 |  |  |  |  |  | 1 |  |  | 1 |  |  |
| cg23348743 |  |  |  |  |  |  | cg23348743 | EBF1 | 3.50E-05 |  |  |  |  |  | 1 |  |  | 1 |  |  |
| cg00068377 |  |  |  |  |  |  | cg00068377 | PRDM16 | 3.50E-05 |  |  |  |  |  | 1 |  |  | 1 |  |  |
| cg09794469 |  |  |  |  |  |  | cg09794469 | TNRC18 | 3.50E-05 |  |  |  |  |  | 1 |  |  | 1 |  |  |
| cg09725686 |  |  |  |  |  |  | cg09725686 |  | 3.50E-05 |  |  |  |  |  | 1 |  |  | 1 |  |  |
| cg18311516 |  |  |  |  |  |  | cg18311516 | DYNC1H1 | 3.52E-05 |  |  |  |  |  | 1 |  |  | 1 |  |  |
| cg15369419 |  |  |  |  |  |  | cg15369419 | KLF7 | 3.52E-05 |  |  |  |  |  | 1 |  |  | 1 |  |  |
| cg11930700 |  |  |  |  |  |  | cg11930700 |  | 3.52E-05 |  |  |  |  |  | 1 |  |  | 1 |  |  |
| cg27130993 |  |  |  |  |  |  | cg27130993 | ABLIM3 | 3.53E-05 |  |  |  |  |  | 1 |  |  | 1 |  |  |
| cg01922433 |  |  |  |  |  |  | cg01922433 | ADAMTSL4 | 3.53E-05 |  |  |  |  |  | 1 |  |  | 1 |  |  |
| cg03708990 |  |  |  |  |  |  | cg03708990 | OSBPL7 | 3.53E-05 |  |  |  |  |  | 1 |  |  | 1 |  |  |
| cg16157016 |  |  |  |  |  |  | cg16157016 | PICK1 | 3.53E-05 |  |  |  |  |  | 1 |  |  | 1 |  |  |
| cg20014778 |  |  |  |  |  |  | cg20014778 | URB2 | 3.54E-05 |  |  |  |  |  | 1 |  |  | 1 |  |  |
| cg16494843 |  |  |  |  |  |  | cg16494843 |  | 3.57E-05 |  |  |  |  |  | 1 |  |  | 1 |  |  |
| cg25371449 |  |  |  |  |  |  | cg25371449 | SNHG3-RCC1 | 3.60E-05 |  |  |  |  |  | 1 |  |  | 1 |  |  |
| cg08431873 |  |  |  |  |  |  | cg08431873 | C11orf73 | 3.61E-05 |  |  |  |  |  | 1 |  |  | 1 |  |  |
| cg07589519 |  |  |  |  |  |  | cg07589519 | MACROD1 | 3.61E-05 |  |  |  |  |  | 1 |  |  | 1 |  |  |
| cg10887309 |  |  |  |  |  |  | cg10887309 | PRKAG1 | 3.61E-05 |  |  |  |  |  | 1 |  |  | 1 |  |  |
| cg19889666 |  |  |  |  |  |  | cg19889666 | TP53AIP1 | 3.61E-05 |  |  |  |  |  | 1 |  |  | 1 |  |  |
| cg24379915 |  |  |  |  |  |  | cg24379915 | DUSP4 | 3.64E-05 |  |  |  |  |  | 1 |  |  | 1 |  |  |
| cg27182159 |  |  |  |  |  |  | cg27182159 | RPS18 | 3.70E-05 |  |  |  |  |  | 1 |  |  | 1 |  |  |
| cg02933375 |  |  |  |  |  |  | cg02933375 | RPTOR | 3.70E-05 |  |  |  |  |  | 1 |  |  | 1 |  |  |
| cg26900750 |  |  |  |  |  |  | cg26900750 | LTBP4 | 3.73E-05 |  |  |  |  |  | 1 |  |  | 1 |  |  |
| cg00794722 |  |  |  |  |  |  | cg00794722 |  | 3.73E-05 |  |  |  |  |  | 1 |  |  | 1 |  |  |
| cg08141342 |  |  |  |  |  |  | cg08141342 | PTCH2 | 3.74E-05 |  |  |  |  |  | 1 |  |  | 1 |  |  |
| cg23117592 |  |  |  |  |  |  | cg23117592 | UNC80 | 3.74E-05 |  |  |  |  |  | 1 |  |  | 1 |  |  |
| cg08704623 |  |  |  |  |  |  | cg08704623 |  | 3.74E-05 |  |  |  |  |  | 1 |  |  | 1 |  |  |
| cg10553894 |  |  |  |  |  |  | cg10553894 | CPT1A | 3.79E-05 |  |  |  |  |  | 1 |  |  | 1 |  |  |
| cg23817637 |  |  |  |  |  |  | cg23817637 | CLRN3 | 3.80E-05 |  |  |  |  |  | 1 |  |  | 1 |  |  |
| cg11925103 |  |  |  |  |  |  | cg11925103 | NDUFS7 | 3.80E-05 |  |  |  |  |  | 1 |  |  | 1 |  |  |
| cg04822973 |  |  |  |  |  |  | cg04822973 | ARG2 | 3.81E-05 |  |  |  |  |  | 1 |  |  | 1 |  |  |
| cg07277624 |  |  |  |  |  |  | cg07277624 | DBNDD2 | 3.82E-05 |  |  |  |  |  | 1 |  |  | 1 |  |  |
| cg16385237 |  |  |  |  |  |  | cg16385237 | NEU3 | 3.82E-05 |  |  |  |  |  | 1 |  |  | 1 |  |  |
| cg18461093 |  |  |  |  |  |  | cg18461093 |  | 3.83E-05 |  |  |  |  |  | 1 |  |  | 1 |  |  |
| cg23879460 |  |  |  |  |  |  | cg23879460 | LOC285370 | 3.85E-05 |  |  |  |  |  | 1 |  |  | 1 |  |  |
| cg21696055 |  |  |  |  |  |  | cg21696055 | ARHGEF10L | 3.87E-05 |  |  |  |  |  | 1 |  |  | 1 |  |  |
| cg17998283 |  |  |  |  |  |  | cg17998283 |  | 3.87E-05 |  |  |  |  |  | 1 |  |  | 1 |  |  |
| cg14007706 |  |  |  |  |  |  | cg14007706 | SLC25A23 | 3.92E-05 |  |  |  |  |  | 1 |  |  | 1 |  |  |
| cg15464363 |  |  |  |  |  |  | cg15464363 | DEPDC5 | 3.93E-05 |  |  |  |  |  | 1 |  |  | 1 |  |  |
| cg05093469 |  |  |  |  |  |  | cg05093469 | FLVCR2 | 3.94E-05 |  |  |  |  |  | 1 |  |  | 1 |  |  |
| cg14414124 |  |  |  |  |  |  | cg14414124 | PPP3R1 | 3.94E-05 |  |  |  |  |  | 1 |  |  | 1 |  |  |
| cg05179805 |  |  |  |  |  |  | cg05179805 |  | 3.94E-05 |  |  |  |  |  | 1 |  |  | 1 |  |  |
| cg09331995 |  |  |  |  |  |  | cg09331995 |  | 3.94E-05 |  |  |  |  |  | 1 |  |  | 1 |  |  |
| cg27194586 |  |  |  |  |  |  | cg27194586 |  | 3.94E-05 |  |  |  |  |  | 1 |  |  | 1 |  |  |
| cg03915940 |  |  |  |  |  |  | cg03915940 | C16orf90 | 3.95E-05 |  |  |  |  |  | 1 |  |  | 1 |  |  |
| cg10778931 |  |  |  |  |  |  | cg10778931 | SCAPER | 3.95E-05 |  |  |  |  |  | 1 |  |  | 1 |  |  |
| cg18821320 |  |  |  |  |  |  | cg18821320 | BAT1 | 3.98E-05 |  |  |  |  |  | 1 |  |  | 1 |  |  |
| cg23430209 |  |  |  |  |  |  | cg23430209 | HCCA2 | 3.98E-05 |  |  |  |  |  | 1 |  |  | 1 |  |  |
| cg16278828 |  |  |  |  |  |  | cg16278828 | MAN2C1 | 3.98E-05 |  |  |  |  |  | 1 |  |  | 1 |  |  |
| cg08993103 |  |  |  |  |  |  | cg08993103 | SEL1L3 | 3.98E-05 |  |  |  |  |  | 1 |  |  | 1 |  |  |
| cg26741280 |  |  |  |  |  |  | cg26741280 | SLC6A4 | 3.98E-05 |  |  |  |  |  | 1 |  |  | 1 |  |  |
| cg18824724 |  |  |  |  |  |  | cg18824724 | SORBS2 | 3.98E-05 |  |  |  |  |  | 1 |  |  | 1 |  |  |
| cg19153095 |  |  |  |  |  |  | cg19153095 | ZFPM2 | 3.98E-05 |  |  |  |  |  | 1 |  |  | 1 |  |  |
| cg04722977 |  |  |  |  |  |  | cg04722977 | ZNF526 | 3.98E-05 |  |  |  |  |  | 1 |  |  | 1 |  |  |
| cg13852730 |  |  |  |  |  |  | cg13852730 |  | 3.98E-05 |  |  |  |  |  | 1 |  |  | 1 |  |  |
| cg21539981 |  |  |  |  |  |  | cg21539981 |  | 3.98E-05 |  |  |  |  |  | 1 |  |  | 1 |  |  |
| cg13849727 |  |  |  |  |  |  | cg13849727 | C9orf93 | 4.01E-05 |  |  |  |  |  | 1 |  |  | 1 |  |  |
| cg13709054 |  |  |  |  |  |  | cg13709054 | DOM3Z | 4.02E-05 |  |  |  |  |  | 1 |  |  | 1 |  |  |
| cg16261572 |  |  |  |  |  |  | cg16261572 | CBY3 | 4.05E-05 |  |  |  |  |  | 1 |  |  | 1 |  |  |
| cg14430629 |  |  |  |  |  |  | cg14430629 | RNF112 | 4.05E-05 |  |  |  |  |  | 1 |  |  | 1 |  |  |
| cg13502545 |  |  |  |  |  |  | cg13502545 |  | 4.05E-05 |  |  |  |  |  | 1 |  |  | 1 |  |  |
| cg23313266 |  |  |  |  |  |  | cg23313266 |  | 4.05E-05 |  |  |  |  |  | 1 |  |  | 1 |  |  |
| cg08371086 |  |  |  |  |  |  | cg08371086 | FAM19A1 | 4.06E-05 |  |  |  |  |  | 1 |  |  | 1 |  |  |
| cg01785490 |  |  |  |  |  |  | cg01785490 |  | 4.08E-05 |  |  |  |  |  | 1 |  |  | 1 |  |  |
| cg22123804 |  |  |  |  |  |  | cg22123804 |  | 4.08E-05 |  |  |  |  |  | 1 |  |  | 1 |  |  |
| cg01233786 |  |  |  |  |  |  | cg01233786 | ATP11A | 4.09E-05 |  |  |  |  |  | 1 |  |  | 1 |  |  |
| cg01592662 |  |  |  |  |  |  | cg01592662 | NTAN1 | 4.09E-05 |  |  |  |  |  | 1 |  |  | 1 |  |  |
| cg12384262 |  |  |  |  |  |  | cg12384262 | PFKL | 4.09E-05 |  |  |  |  |  | 1 |  |  | 1 |  |  |
| cg10446401 |  |  |  |  |  |  | cg10446401 | RPL31 | 4.09E-05 |  |  |  |  |  | 1 |  |  | 1 |  |  |
| cg03859106 |  |  |  |  |  |  | cg03859106 |  | 4.09E-05 |  |  |  |  |  | 1 |  |  | 1 |  |  |
| cg12752325 |  |  |  |  |  |  | cg12752325 |  | 4.09E-05 |  |  |  |  |  | 1 |  |  | 1 |  |  |
| cg12623107 |  |  |  |  |  |  | cg12623107 | GTF2H1 | 4.11E-05 |  |  |  |  |  | 1 |  |  | 1 |  |  |
| cg25295726 |  |  |  |  |  |  | cg25295726 | FUT11 | 4.12E-05 |  |  |  |  |  | 1 |  |  | 1 |  |  |
| cg04337618 |  |  |  |  |  |  | cg04337618 | HAGHL | 4.12E-05 |  |  |  |  |  | 1 |  |  | 1 |  |  |
| cg16677528 |  |  |  |  |  |  | cg16677528 | IL1F7 | 4.12E-05 |  |  |  |  |  | 1 |  |  | 1 |  |  |
| cg14614754 |  |  |  |  |  |  | cg14614754 |  | 4.12E-05 |  |  |  |  |  | 1 |  |  | 1 |  |  |
| cg24385334 |  |  |  |  |  |  | cg24385334 | ARHGAP22 | 4.14E-05 |  |  |  |  |  | 1 |  |  | 1 |  |  |
| cg11543196 |  |  |  |  |  |  | cg11543196 | CCDC49 | 4.15E-05 |  |  |  |  |  | 1 |  |  | 1 |  |  |
| cg15911114 |  |  |  |  |  |  | cg15911114 | FST | 4.15E-05 |  |  |  |  |  | 1 |  |  | 1 |  |  |
| cg18910630 |  |  |  |  |  |  | cg18910630 | TFCP2L1 | 4.15E-05 |  |  |  |  |  | 1 |  |  | 1 |  |  |
| cg00959749 |  |  |  |  |  |  | cg00959749 | TMEM48 | 4.15E-05 |  |  |  |  |  | 1 |  |  | 1 |  |  |
| cg08524717 |  |  |  |  |  |  | cg08524717 |  | 4.15E-05 |  |  |  |  |  | 1 |  |  | 1 |  |  |
| cg24340655 |  |  |  |  |  |  | cg24340655 |  | 4.15E-05 |  |  |  |  |  | 1 |  |  | 1 |  |  |
| cg08099570 |  |  |  |  |  |  | cg08099570 | CNGB1 | 4.16E-05 |  |  |  |  |  | 1 |  |  | 1 |  |  |
| cg08240592 |  |  |  |  |  |  | cg08240592 | C1orf107 | 4.20E-05 |  |  |  |  |  | 1 |  |  | 1 |  |  |
| cg17842912 |  |  |  |  |  |  | cg17842912 | CXXC5 | 4.20E-05 |  |  |  |  |  | 1 |  |  | 1 |  |  |
| cg15386853 |  |  |  |  |  |  | cg15386853 | PRDM16 | 4.20E-05 |  |  |  |  |  | 1 |  |  | 1 |  |  |
| cg00086809 |  |  |  |  |  |  | cg00086809 | RAB40C | 4.20E-05 |  |  |  |  |  | 1 |  |  | 1 |  |  |
| cg09801837 |  |  |  |  |  |  | cg09801837 |  | 4.21E-05 |  |  |  |  |  | 1 |  |  | 1 |  |  |
| cg03621406 |  |  |  |  |  |  | cg03621406 | FBXL12 | 4.22E-05 |  |  |  |  |  | 1 |  |  | 1 |  |  |
| cg06026375 |  |  |  |  |  |  | cg06026375 | PMCH | 4.22E-05 |  |  |  |  |  | 1 |  |  | 1 |  |  |
| cg08062329 |  |  |  |  |  |  | cg08062329 | PRR3 | 4.22E-05 |  |  |  |  |  | 1 |  |  | 1 |  |  |
| cg11814935 |  |  |  |  |  |  | cg11814935 | RABIF | 4.22E-05 |  |  |  |  |  | 1 |  |  | 1 |  |  |
| cg15734706 |  |  |  |  |  |  | cg15734706 | RARG | 4.22E-05 |  |  |  |  |  | 1 |  |  | 1 |  |  |
| cg01700035 |  |  |  |  |  |  | cg01700035 | ZNF668 | 4.22E-05 |  |  |  |  |  | 1 |  |  | 1 |  |  |
| cg25490241 |  |  |  |  |  |  | cg25490241 |  | 4.22E-05 |  |  |  |  |  | 1 |  |  | 1 |  |  |
| cg25618672 |  |  |  |  |  |  | cg25618672 |  | 4.22E-05 |  |  |  |  |  | 1 |  |  | 1 |  |  |
| cg27536870 |  |  |  |  |  |  | cg27536870 |  | 4.22E-05 |  |  |  |  |  | 1 |  |  | 1 |  |  |
| cg00475815 |  |  |  |  |  |  | cg00475815 | SENP5 | 4.25E-05 |  |  |  |  |  | 1 |  |  | 1 |  |  |
| cg22153062 |  |  |  |  |  |  | cg22153062 | TUBB6 | 4.26E-05 |  |  |  |  |  | 1 |  |  | 1 |  |  |
| cg14429979 |  |  |  |  |  |  | cg14429979 | AP2A2 | 4.27E-05 |  |  |  |  |  | 1 |  |  | 1 |  |  |
| cg01209150 |  |  |  |  |  |  | cg01209150 |  | 4.27E-05 |  |  |  |  |  | 1 |  |  | 1 |  |  |
| cg26346930 |  |  |  |  |  |  | cg26346930 |  | 4.27E-05 |  |  |  |  |  | 1 |  |  | 1 |  |  |
| cg23252848 |  |  |  |  |  |  | cg23252848 | YBX1 | 4.29E-05 |  |  |  |  |  | 1 |  |  | 1 |  |  |
| cg13286990 |  |  |  |  |  |  | cg13286990 | LOC100132831 | 4.31E-05 |  |  |  |  |  | 1 |  |  | 1 |  |  |
| cg20152152 |  |  |  |  |  |  | cg20152152 | GPR152 | 4.37E-05 |  |  |  |  |  | 1 |  |  | 1 |  |  |
| cg23555340 |  |  |  |  |  |  | cg23555340 |  | 4.39E-05 |  |  |  |  |  | 1 |  |  | 1 |  |  |
| cg12482260 |  |  |  |  |  |  | cg12482260 | NDUFAF4 | 4.41E-05 |  |  |  |  |  | 1 |  |  | 1 |  |  |
| cg10716494 |  |  |  |  |  |  | cg10716494 | MAML2 | 4.44E-05 |  |  |  |  |  | 1 |  |  | 1 |  |  |
| cg12279175 |  |  |  |  |  |  | cg12279175 |  | 4.46E-05 |  |  |  |  |  | 1 |  |  | 1 |  |  |
| cg24876404 |  |  |  |  |  |  | cg24876404 |  | 4.46E-05 |  |  |  |  |  | 1 |  |  | 1 |  |  |
| cg14878128 |  |  |  |  |  |  | cg14878128 | ABCB5 | 4.51E-05 |  |  |  |  |  | 1 |  |  | 1 |  |  |
| cg05331472 |  |  |  |  |  |  | cg05331472 | BCAS4 | 4.51E-05 |  |  |  |  |  | 1 |  |  | 1 |  |  |
| cg25147684 |  |  |  |  |  |  | cg25147684 | GPR78 | 4.51E-05 |  |  |  |  |  | 1 |  |  | 1 |  |  |
| cg26177629 |  |  |  |  |  |  | cg26177629 | RAB18 | 4.51E-05 |  |  |  |  |  | 1 |  |  | 1 |  |  |
| cg06991955 |  |  |  |  |  |  | cg06991955 | TSC2 | 4.51E-05 |  |  |  |  |  | 1 |  |  | 1 |  |  |
| cg02759193 |  |  |  |  |  |  | cg02759193 |  | 4.51E-05 |  |  |  |  |  | 1 |  |  | 1 |  |  |
| cg07346171 |  |  |  |  |  |  | cg07346171 | EPB41 | 4.52E-05 |  |  |  |  |  | 1 |  |  | 1 |  |  |
| cg02832305 |  |  |  |  |  |  | cg02832305 | FCGR2C | 4.52E-05 |  |  |  |  |  | 1 |  |  | 1 |  |  |
| cg06989074 |  |  |  |  |  |  | cg06989074 | LRP5 | 4.52E-05 |  |  |  |  |  | 1 |  |  | 1 |  |  |
| cg07267166 |  |  |  |  |  |  | cg07267166 | ZNF323 | 4.52E-05 |  |  |  |  |  | 1 |  |  | 1 |  |  |
| cg07399636 |  |  |  |  |  |  | cg07399636 |  | 4.52E-05 |  |  |  |  |  | 1 |  |  | 1 |  |  |
| cg19834585 |  |  |  |  |  |  | cg19834585 | GALNT9 | 4.53E-05 |  |  |  |  |  | 1 |  |  | 1 |  |  |
| cg23008083 |  |  |  |  |  |  | cg23008083 | ITGB6 | 4.53E-05 |  |  |  |  |  | 1 |  |  | 1 |  |  |
| cg27118937 |  |  |  |  |  |  | cg27118937 | LMF1 | 4.53E-05 |  |  |  |  |  | 1 |  |  | 1 |  |  |
| cg18831262 |  |  |  |  |  |  | cg18831262 | PNPLA6 | 4.53E-05 |  |  |  |  |  | 1 |  |  | 1 |  |  |
| cg06280512 |  |  |  |  |  |  | cg06280512 | PRKD2 | 4.53E-05 |  |  |  |  |  | 1 |  |  | 1 |  |  |
| cg14781190 |  |  |  |  |  |  | cg14781190 | SERINC2 | 4.53E-05 |  |  |  |  |  | 1 |  |  | 1 |  |  |
| cg25680105 |  |  |  |  |  |  | cg25680105 |  | 4.53E-05 |  |  |  |  |  | 1 |  |  | 1 |  |  |
| cg21223803 |  |  |  |  |  |  | cg21223803 | DACT2 | 4.54E-05 |  |  |  |  |  | 1 |  |  | 1 |  |  |
| cg24412117 |  |  |  |  |  |  | cg24412117 | LOC100188947 | 4.54E-05 |  |  |  |  |  | 1 |  |  | 1 |  |  |
| cg08598287 |  |  |  |  |  |  | cg08598287 |  | 4.56E-05 |  |  |  |  |  | 1 |  |  | 1 |  |  |
| cg10795359 |  |  |  |  |  |  | cg10795359 | AKR1B1 | 4.58E-05 |  |  |  |  |  | 1 |  |  | 1 |  |  |
| cg02706575 |  |  |  |  |  |  | cg02706575 | CCL13 | 4.61E-05 |  |  |  |  |  | 1 |  |  | 1 |  |  |
| cg17306814 |  |  |  |  |  |  | cg17306814 | SLC7A14 | 4.61E-05 |  |  |  |  |  | 1 |  |  | 1 |  |  |
| cg16681031 |  |  |  |  |  |  | cg16681031 | RNF213 | 4.62E-05 |  |  |  |  |  | 1 |  |  | 1 |  |  |
| cg20536794 |  |  |  |  |  |  | cg20536794 |  | 4.65E-05 |  |  |  |  |  | 1 |  |  | 1 |  |  |
| cg10094238 |  |  |  |  |  |  | cg10094238 | ARHGAP27 | 4.66E-05 |  |  |  |  |  | 1 |  |  | 1 |  |  |
| cg08740477 |  |  |  |  |  |  | cg08740477 | FER | 4.66E-05 |  |  |  |  |  | 1 |  |  | 1 |  |  |
| cg06500120 |  |  |  |  |  |  | cg06500120 | GSX2 | 4.66E-05 |  |  |  |  |  | 1 |  |  | 1 |  |  |
| cg12168066 |  |  |  |  |  |  | cg12168066 |  | 4.66E-05 |  |  |  |  |  | 1 |  |  | 1 |  |  |
| cg25673945 |  |  |  |  |  |  | cg25673945 |  | 4.66E-05 |  |  |  |  |  | 1 |  |  | 1 |  |  |
| cg25987208 |  |  |  |  |  |  | cg25987208 |  | 4.66E-05 |  |  |  |  |  | 1 |  |  | 1 |  |  |
| cg01769354 |  |  |  |  |  |  | cg01769354 |  | 4.68E-05 |  |  |  |  |  | 1 |  |  | 1 |  |  |
| cg09048334 |  |  |  |  |  |  | cg09048334 |  | 4.69E-05 |  |  |  |  |  | 1 |  |  | 1 |  |  |
| cg24162965 |  |  |  |  |  |  | cg24162965 |  | 4.69E-05 |  |  |  |  |  | 1 |  |  | 1 |  |  |
| cg23963476 |  |  |  |  |  |  | cg23963476 | SMARCA4 | 4.70E-05 |  |  |  |  |  | 1 |  |  | 1 |  |  |
| cg17750043 |  |  |  |  |  |  | cg17750043 | TMEM211 | 4.70E-05 |  |  |  |  |  | 1 |  |  | 1 |  |  |
| cg00681665 |  |  |  |  |  |  | cg00681665 | ALOX12B | 4.72E-05 |  |  |  |  |  | 1 |  |  | 1 |  |  |
| cg27063327 |  |  |  |  |  |  | cg27063327 | BCAS3 | 4.72E-05 |  |  |  |  |  | 1 |  |  | 1 |  |  |
| cg12747410 |  |  |  |  |  |  | cg12747410 | MS4A15 | 4.72E-05 |  |  |  |  |  | 1 |  |  | 1 |  |  |
| cg11023992 |  |  |  |  |  |  | cg11023992 | OR7E156P | 4.72E-05 |  |  |  |  |  | 1 |  |  | 1 |  |  |
| cg22857604 |  |  |  |  |  |  | cg22857604 | RASSF5 | 4.72E-05 |  |  |  |  |  | 1 |  |  | 1 |  |  |
| cg20752695 |  |  |  |  |  |  | cg20752695 |  | 4.72E-05 |  |  |  |  |  | 1 |  |  | 1 |  |  |
| cg11130097 |  |  |  |  |  |  | cg11130097 | C10orf75 | 4.74E-05 |  |  |  |  |  | 1 |  |  | 1 |  |  |
| cg00918738 |  |  |  |  |  |  | cg00918738 | TCF15 | 4.74E-05 |  |  |  |  |  | 1 |  |  | 1 |  |  |
| cg24408603 |  |  |  |  |  |  | cg24408603 | TOR1AIP1 | 4.74E-05 |  |  |  |  |  | 1 |  |  | 1 |  |  |
| cg05213267 |  |  |  |  |  |  | cg05213267 | DIP2C | 4.76E-05 |  |  |  |  |  | 1 |  |  | 1 |  |  |
| cg15009352 |  |  |  |  |  |  | cg15009352 | HSPG2 | 4.76E-05 |  |  |  |  |  | 1 |  |  | 1 |  |  |
| cg17727418 |  |  |  |  |  |  | cg17727418 | NRG3 | 4.76E-05 |  |  |  |  |  | 1 |  |  | 1 |  |  |
| cg03299095 |  |  |  |  |  |  | cg03299095 | SNORD115-11 | 4.76E-05 |  |  |  |  |  | 1 |  |  | 1 |  |  |
| cg05575273 |  |  |  |  |  |  | cg05575273 |  | 4.76E-05 |  |  |  |  |  | 1 |  |  | 1 |  |  |
| cg14910395 |  |  |  |  |  |  | cg14910395 | GRIN2D | 4.77E-05 |  |  |  |  |  | 1 |  |  | 1 |  |  |
| cg05107228 |  |  |  |  |  |  | cg05107228 | HDAC4 | 4.77E-05 |  |  |  |  |  | 1 |  |  | 1 |  |  |
| cg13444392 |  |  |  |  |  |  | cg13444392 |  | 4.77E-05 |  |  |  |  |  | 1 |  |  | 1 |  |  |
| cg05844366 |  |  |  |  |  |  | cg05844366 |  | 4.78E-05 |  |  |  |  |  | 1 |  |  | 1 |  |  |
| cg24732062 |  |  |  |  |  |  | cg24732062 |  | 4.78E-05 |  |  |  |  |  | 1 |  |  | 1 |  |  |
| cg14787287 |  |  |  |  |  |  | cg14787287 | ABCA4 | 4.79E-05 |  |  |  |  |  | 1 |  |  | 1 |  |  |
| cg25766801 |  |  |  |  |  |  | cg25766801 | CSMD1 | 4.81E-05 |  |  |  |  |  | 1 |  |  | 1 |  |  |
| cg12617684 |  |  |  |  |  |  | cg12617684 |  | 4.81E-05 |  |  |  |  |  | 1 |  |  | 1 |  |  |
| cg21734175 |  |  |  |  |  |  | cg21734175 |  | 4.81E-05 |  |  |  |  |  | 1 |  |  | 1 |  |  |
| cg25747655 |  |  |  |  |  |  | cg25747655 |  | 4.81E-05 |  |  |  |  |  | 1 |  |  | 1 |  |  |
| cg07604202 |  |  |  |  |  |  | cg07604202 | ZFP64 | 4.85E-05 |  |  |  |  |  | 1 |  |  | 1 |  |  |
| cg09749862 |  |  |  |  |  |  | cg09749862 | LRP1 | 4.87E-05 |  |  |  |  |  | 1 |  |  | 1 |  |  |
| cg24926253 |  |  |  |  |  |  | cg24926253 | SLC22A16 | 4.89E-05 |  |  |  |  |  | 1 |  |  | 1 |  |  |
| cg20297940 |  |  |  |  |  |  | cg20297940 |  | 4.89E-05 |  |  |  |  |  | 1 |  |  | 1 |  |  |
| cg08079331 |  |  |  |  |  |  | cg08079331 | TMCC3 | 4.91E-05 |  |  |  |  |  | 1 |  |  | 1 |  |  |
| cg01476047 |  |  |  |  |  |  | cg01476047 | ASCC1 | 4.92E-05 |  |  |  |  |  | 1 |  |  | 1 |  |  |
| cg16555896 |  |  |  |  |  |  | cg16555896 | MORN1 | 4.92E-05 |  |  |  |  |  | 1 |  |  | 1 |  |  |
| cg12660445 |  |  |  |  |  |  | cg12660445 | SNORD18A | 4.92E-05 |  |  |  |  |  | 1 |  |  | 1 |  |  |
| cg10415122 |  |  |  |  |  |  | cg10415122 | CSPG4 | 4.96E-05 |  |  |  |  |  | 1 |  |  | 1 |  |  |
| cg24790419 |  |  |  |  |  |  | cg24790419 | KIAA1683 | 4.99E-05 |  |  |  |  |  | 1 |  |  | 1 |  |  |
| cg26923014 |  |  |  |  |  |  | cg26923014 |  | 4.99E-05 |  |  |  |  |  | 1 |  |  | 1 |  |  |
| cg02423618 |  |  |  |  |  |  | cg02423618 | SPATA8 | 5.00E-05 |  |  |  |  |  | 1 |  |  | 1 |  |  |
| cg02709840 |  |  |  |  |  |  | cg02709840 | CBFB | 5.02E-05 |  |  |  |  |  | 1 |  |  | 1 |  |  |
| cg01552711 |  |  |  |  |  |  | cg01552711 | IL17D | 5.02E-05 |  |  |  |  |  | 1 |  |  | 1 |  |  |
| cg03930929 |  |  |  |  |  |  | cg03930929 | LRRN1 | 5.02E-05 |  |  |  |  |  | 1 |  |  | 1 |  |  |
| cg17872658 |  |  |  |  |  |  | cg17872658 | RPTOR | 5.02E-05 |  |  |  |  |  | 1 |  |  | 1 |  |  |
| cg15034413 |  |  |  |  |  |  | cg15034413 |  | 5.02E-05 |  |  |  |  |  | 1 |  |  | 1 |  |  |
| cg16280946 |  |  |  |  |  |  | cg16280946 |  | 5.02E-05 |  |  |  |  |  | 1 |  |  | 1 |  |  |
| cg03485672 |  |  |  |  |  |  | cg03485672 |  | 5.03E-05 |  |  |  |  |  | 1 |  |  | 1 |  |  |
| cg08773029 |  |  |  |  |  |  | cg08773029 | ANKMY1 | 5.04E-05 |  |  |  |  |  | 1 |  |  | 1 |  |  |
| cg16724696 |  |  |  |  |  |  | cg16724696 | HINFP | 5.10E-05 |  |  |  |  |  | 1 |  |  | 1 |  |  |
| cg05880945 |  |  |  |  |  |  | cg05880945 | C9orf122 | 5.11E-05 |  |  |  |  |  | 1 |  |  | 1 |  |  |
| cg05633605 |  |  |  |  |  |  | cg05633605 | ANKRD55 | 5.13E-05 |  |  |  |  |  | 1 |  |  | 1 |  |  |
| cg17820989 |  |  |  |  |  |  | cg17820989 | MFSD3 | 5.13E-05 |  |  |  |  |  | 1 |  |  | 1 |  |  |
| cg05725703 |  |  |  |  |  |  | cg05725703 | MYL3 | 5.13E-05 |  |  |  |  |  | 1 |  |  | 1 |  |  |
| cg07205462 |  |  |  |  |  |  | cg07205462 | SNRPB2 | 5.13E-05 |  |  |  |  |  | 1 |  |  | 1 |  |  |
| cg25428612 |  |  |  |  |  |  | cg25428612 | STAT4 | 5.13E-05 |  |  |  |  |  | 1 |  |  | 1 |  |  |
| cg02283353 |  |  |  |  |  |  | cg02283353 | POLRMT | 5.14E-05 |  |  |  |  |  | 1 |  |  | 1 |  |  |
| cg19418648 |  |  |  |  |  |  | cg19418648 | RGS14 | 5.14E-05 |  |  |  |  |  | 1 |  |  | 1 |  |  |
| cg09538725 |  |  |  |  |  |  | cg09538725 |  | 5.14E-05 |  |  |  |  |  | 1 |  |  | 1 |  |  |
| cg01337429 |  |  |  |  |  |  | cg01337429 | NTN3 | 5.16E-05 |  |  |  |  |  | 1 |  |  | 1 |  |  |
| cg15587018 |  |  |  |  |  |  | cg15587018 | SUPT3H | 5.17E-05 |  |  |  |  |  | 1 |  |  | 1 |  |  |
| cg03516394 |  |  |  |  |  |  | cg03516394 |  | 5.17E-05 |  |  |  |  |  | 1 |  |  | 1 |  |  |
| cg02047547 |  |  |  |  |  |  | cg02047547 | ITPRIPL2 | 5.18E-05 |  |  |  |  |  | 1 |  |  | 1 |  |  |
| cg16834212 |  |  |  |  |  |  | cg16834212 | SUCLG2 | 5.18E-05 |  |  |  |  |  | 1 |  |  | 1 |  |  |
| cg01704976 |  |  |  |  |  |  | cg01704976 | TWF2 | 5.18E-05 |  |  |  |  |  | 1 |  |  | 1 |  |  |
| cg27074221 |  |  |  |  |  |  | cg27074221 | MIR495 | 5.20E-05 |  |  |  |  |  | 1 |  |  | 1 |  |  |
| cg09740598 |  |  |  |  |  |  | cg09740598 | SLC13A2 | 5.20E-05 |  |  |  |  |  | 1 |  |  | 1 |  |  |
| cg16754643 |  |  |  |  |  |  | cg16754643 | ZNF605 | 5.20E-05 |  |  |  |  |  | 1 |  |  | 1 |  |  |
| cg09075525 |  |  |  |  |  |  | cg09075525 |  | 5.20E-05 |  |  |  |  |  | 1 |  |  | 1 |  |  |
| cg23473088 |  |  |  |  |  |  | cg23473088 |  | 5.20E-05 |  |  |  |  |  | 1 |  |  | 1 |  |  |
| cg09376008 |  |  |  |  |  |  | cg09376008 |  | 5.22E-05 |  |  |  |  |  | 1 |  |  | 1 |  |  |
| cg23516342 |  |  |  |  |  |  | cg23516342 | DCAF8L2 | 5.25E-05 |  |  |  |  |  | 1 |  |  | 1 |  |  |
| cg01427976 |  |  |  |  |  |  | cg01427976 | NDRG3 | 5.25E-05 |  |  |  |  |  | 1 |  |  | 1 |  |  |
| cg03793872 |  |  |  |  |  |  | cg03793872 |  | 5.25E-05 |  |  |  |  |  | 1 |  |  | 1 |  |  |
| cg15874144 |  |  |  |  |  |  | cg15874144 |  | 5.25E-05 |  |  |  |  |  | 1 |  |  | 1 |  |  |
| cg03925157 |  |  |  |  |  |  | cg03925157 | DSTN | 5.27E-05 |  |  |  |  |  | 1 |  |  | 1 |  |  |
| cg02812891 |  |  |  |  |  |  | cg02812891 | ECEL1P2 | 5.29E-05 |  |  |  |  |  | 1 |  |  | 1 |  |  |
| cg17628700 |  |  |  |  |  |  | cg17628700 | MDK | 5.29E-05 |  |  |  |  |  | 1 |  |  | 1 |  |  |
| cg05853039 |  |  |  |  |  |  | cg05853039 | MSI2 | 5.29E-05 |  |  |  |  |  | 1 |  |  | 1 |  |  |
| cg09834444 |  |  |  |  |  |  | cg09834444 | SPIRE2 | 5.29E-05 |  |  |  |  |  | 1 |  |  | 1 |  |  |
| cg25468907 |  |  |  |  |  |  | cg25468907 | TNNI1 | 5.29E-05 |  |  |  |  |  | 1 |  |  | 1 |  |  |
| cg08040148 |  |  |  |  |  |  | cg08040148 |  | 5.29E-05 |  |  |  |  |  | 1 |  |  | 1 |  |  |
| cg27640712 |  |  |  |  |  |  | cg27640712 |  | 5.32E-05 |  |  |  |  |  | 1 |  |  | 1 |  |  |
| cg03449125 |  |  |  |  |  |  | cg03449125 | CAPN5 | 5.40E-05 |  |  |  |  |  | 1 |  |  | 1 |  |  |
| cg12720459 |  |  |  |  |  |  | cg12720459 | PELI2 | 5.40E-05 |  |  |  |  |  | 1 |  |  | 1 |  |  |
| cg10336578 |  |  |  |  |  |  | cg10336578 |  | 5.40E-05 |  |  |  |  |  | 1 |  |  | 1 |  |  |
| cg25265930 |  |  |  |  |  |  | cg25265930 | HLA-DMB | 5.41E-05 |  |  |  |  |  | 1 |  |  | 1 |  |  |
| cg20986608 |  |  |  |  |  |  | cg20986608 | SART1 | 5.41E-05 |  |  |  |  |  | 1 |  |  | 1 |  |  |
| cg01342858 |  |  |  |  |  |  | cg01342858 |  | 5.41E-05 |  |  |  |  |  | 1 |  |  | 1 |  |  |
| cg14778576 |  |  |  |  |  |  | cg14778576 | PPM1L | 5.42E-05 |  |  |  |  |  | 1 |  |  | 1 |  |  |
| cg13471374 |  |  |  |  |  |  | cg13471374 | RCL1 | 5.42E-05 |  |  |  |  |  | 1 |  |  | 1 |  |  |
| cg01123250 |  |  |  |  |  |  | cg01123250 | UNC80 | 5.42E-05 |  |  |  |  |  | 1 |  |  | 1 |  |  |
| cg15433604 |  |  |  |  |  |  | cg15433604 | UPF1 | 5.43E-05 |  |  |  |  |  | 1 |  |  | 1 |  |  |
| cg15110219 |  |  |  |  |  |  | cg15110219 | PADI4 | 5.44E-05 |  |  |  |  |  | 1 |  |  | 1 |  |  |
| cg19998148 |  |  |  |  |  |  | cg19998148 | CWF19L2 | 5.48E-05 |  |  |  |  |  | 1 |  |  | 1 |  |  |
| cg23576855 |  |  |  | cg23576855 | AHRR | 1.08E-14 | cg23576855 | AHRR | 3.37E-12 |  |  | 1 |  |  | 1 |  |  |  | 1 |  |
| cg06972908 |  |  |  | cg06972908 | ITGAL | 1.67E-14 | cg06972908 | ITGAL | 8.41E-07 |  |  | 1 |  |  | 1 |  |  |  | 1 |  |
| cg11556164 |  |  |  | cg11556164 | LRRN3 | 6.97E-09 | cg11556164 | LRRN3 | 4.43E-06 |  |  | 1 |  |  | 1 |  |  |  | 1 |  |
| cg20778199 |  |  |  | cg20778199 | NA | 1.11E-12 | cg20778199 |  | 1.31E-05 |  |  | 1 |  |  | 1 |  |  |  | 1 |  |
| cg06060868 |  |  |  | cg06060868 | SDHA | 4.67E-08 | cg06060868 | SDHA | 4.00E-05 |  |  | 1 |  |  | 1 |  |  |  | 1 |  |
| cg04716530 |  |  |  | cg04716530 | ITGAL | 1.08E-12 | cg04716530 | ITGAL | 4.02E-05 |  |  | 1 |  |  | 1 |  |  |  | 1 |  |
| cg17287155 |  |  |  | cg17287155 | AHRR | 9.67E-17 | cg17287155 | AHRR | 4.74E-05 |  |  | 1 |  |  | 1 |  |  |  | 1 |  |
| cg14817490 | cg14817490 | AHRR | 4.08E-36 | cg14817490 | AHRR | 4.22E-37 |  |  |  |  |  |  | 1 | 1 |  |  | 1 |  |  |  |
| cg15342087 | cg15342087 | xaxaxaxaxaxaxaxaxaxaxaxaxaxaxaxaxaxaxaxaxaxaxaxaxaxaxaxaxaxaxaxa | 1.71E-28 | cg15342087 | NA | 2.09E-32 |  |  |  |  |  |  | 1 | 1 |  |  | 1 |  |  |  |
| cg11902777 | cg11902777 | AHRR | 4.04E-27 | cg11902777 | AHRR | 4.04E-14 |  |  |  |  |  |  | 1 | 1 |  |  | 1 |  |  |  |
| cg25189904 | cg25189904 | GNG12 | 1.71E-26 | cg25189904 | GNG12 | 1.25E-32 |  |  |  |  |  |  | 1 | 1 |  |  | 1 |  |  |  |
| cg27241845 | cg27241845 | xaxaxaxaxaxaxaxaxaxaxaxaxaxaxaxaxaxaxaxaxaxaxaxaxaxaxaxaxaxaxaxa | 2.79E-21 | cg27241845 | NA | 5.77E-26 |  |  |  |  |  |  | 1 | 1 |  |  | 1 |  |  |  |
| cg22132788 | cg22132788 | MYO1G | 1.99E-20 | cg22132788 | MYO1G | 3.57E-18 |  |  |  |  |  |  | 1 | 1 |  |  | 1 |  |  |  |
| cg06644428 | cg06644428 | ALPPL2bALPPL2bALPPL2bALPPL2bALPPL2bALPPL2bALPPL2bALPPL2bALPPL2bALPPL2bALPPL2bALPPL2bALPPL2bALPPL2bALPPL2bALPPL2bALPPL2bALPPL2bALPPL2bALPPL2bALPPL2bALPPL2bALPPL2bALPPL2bALPPL2bALPPL2bALPPL2bALPPL2bALPPL2bALPPL2bALPPL2bALPPL2b | 6.37E-17 | cg06644428 | NA | 8.48E-28 |  |  |  |  |  |  | 1 | 1 |  |  | 1 |  |  |  |
| cg27537125 | cg27537125 | xaxaxaxaxaxaxaxaxaxaxaxaxaxaxaxaxaxaxaxaxaxaxaxaxaxaxaxaxaxaxaxa | 2.70E-16 | cg27537125 | NA | 2.55E-26 |  |  |  |  |  |  | 1 | 1 |  |  | 1 |  |  |  |
| cg12803068 | cg12803068 | MYO1G | 7.08E-16 | cg12803068 | MYO1G | 2.53E-15 |  |  |  |  |  |  | 1 | 1 |  |  | 1 |  |  |  |
| cg03604011 | cg03604011 | AHRR | 7.87E-16 | cg03604011 | AHRR | 1.65E-14 |  |  |  |  |  |  | 1 | 1 |  |  | 1 |  |  |  |
| cg25949550 | cg25949550 | CNTNAP2 | 4.53E-16 | cg25949550 | CNTNAP2 | 3.09E-18 |  |  |  |  |  |  | 1 | 1 |  |  | 1 |  |  |  |
| cg07826859 | cg07826859 | MYO1G | 8.03E-13 | cg07826859 | MYO1G | 2.69E-23 |  |  |  |  |  |  | 1 | 1 |  |  | 1 |  |  |  |
| cg23973524 | cg23973524 | CRTC1 | 1.23E-11 | cg23973524 | CRTC1 | 9.15E-12 |  |  |  |  |  |  | 1 | 1 |  |  | 1 |  |  |  |
| cg00501876 | cg00501876 | CSRNP1 | 1.39E-11 | cg00501876 | CSRNP1 | 1.03E-16 |  |  |  |  |  |  | 1 | 1 |  |  | 1 |  |  |  |
| cg03274391 | cg03274391 | xaxaxaxaxaxaxaxaxaxaxaxaxaxaxaxaxaxaxaxaxaxaxaxaxaxaxaxaxaxaxa | 1.67E-11 |  |  |  |  |  |  |  | 1 |  |  | 1 |  |  |  |  |  |  |
| cg24996979 | cg24996979 | C14orf43 | 6.84E-11 | cg24996979 | C14orf43 | 1.03E-08 |  |  |  |  |  |  | 1 | 1 |  |  | 1 |  |  |  |
| cg23161492 | cg23161492 | ANPEP | 1.12E-10 | cg23161492 | ANPEP | 8.68E-19 |  |  |  |  |  |  | 1 | 1 |  |  | 1 |  |  |  |
| cg21322436 | cg21322436 | CNTNAP2 | 1.43E-10 | cg21322436 | CNTNAP2 | 1.49E-24 |  |  |  |  |  |  | 1 | 1 |  |  | 1 |  |  |  |
| cg09099830 | cg09099830 | ITGAL | 1.55E-10 | cg09099830 | ITGAL | 5.98E-17 |  |  |  |  |  |  | 1 | 1 |  |  | 1 |  |  |  |
| cg11554391 | cg11554391 | AHRR | 5.07E-10 | cg11554391 | AHRR | 5.91E-12 |  |  |  |  |  |  | 1 | 1 |  |  | 1 |  |  |  |
| cg14753356 | cg14753356 | xaxaxaxaxaxaxaxaxaxaxaxaxaxaxaxaxaxaxaxaxaxaxaxaxaxaxaxaxaxaxa | 8.14E-10 | cg14753356 | NA | 3.55E-34 |  |  |  |  |  |  | 1 | 1 |  |  | 1 |  |  |  |
| cg23079012 | cg23079012 | xaxaxaxaxaxaxaxaxaxaxaxaxaxaxaxaxaxaxaxaxaxaxaxaxaxaxaxaxaxaxa | 8.29E-10 |  |  |  |  |  |  |  | 1 |  |  | 1 |  |  |  |  |  |  |
| cg13193840 | cg13193840 | ALPPL2bALPPL2bALPPL2bALPPL2bALPPL2bALPPL2bALPPL2bALPPL2bALPPL2bALPPL2bALPPL2bALPPL2bALPPL2bALPPL2bALPPL2bALPPL2bALPPL2bALPPL2bALPPL2bALPPL2bALPPL2bALPPL2bALPPL2bALPPL2bALPPL2bALPPL2bALPPL2bALPPL2bALPPL2bALPPL2bALPPL2b | 1.53E-09 | cg13193840 | NA | 6.96E-16 |  |  |  |  |  |  | 1 | 1 |  |  | 1 |  |  |  |
| cg05673882 | cg05673882 | POLK | 4.49E-09 | cg05673882 | POLK | 6.58E-11 |  |  |  |  |  |  | 1 | 1 |  |  | 1 |  |  |  |
| cg11660018 | cg11660018 | PRSS23 | 1.29E-08 | cg11660018 | PRSS23 | 6.89E-30 |  |  |  |  |  |  | 1 | 1 |  |  | 1 |  |  |  |
| cg23480021 | cg23480021 | xaxaxaxaxaxaxaxaxaxaxaxaxaxaxaxaxaxaxaxaxaxaxaxaxaxaxaxaxaxaxa | 2.67E-08 |  |  |  |  |  |  |  | 1 |  |  | 1 |  |  |  |  |  |  |
| cg05194346 | cg05194346 | xaxaxaxaxaxaxaxaxaxaxaxaxaxaxaxaxaxaxaxaxaxaxaxaxaxaxaxaxaxaxa | 3.36E-08 |  |  |  |  |  |  |  | 1 |  |  | 1 |  |  |  |  |  |  |
| cg26963277 | cg26963277 | KCNQ1OT1 | 4.52E-09 | cg26963277 | KCNQ1OT1 | 9.93E-18 |  |  |  |  |  |  | 1 | 1 |  |  | 1 |  |  |  |
| cg07339236 | cg07339236 | ATP9A | 1.57E-07 | cg07339236 | ATP9A | 6.76E-13 |  |  |  |  |  |  | 1 | 1 |  |  | 1 |  |  |  |
| cg06635952 | cg06635952 | ANXA4 | 2.37E-07 |  |  |  |  |  |  |  | 1 |  |  | 1 |  |  |  |  |  |  |
| cg21393163 | cg21393163 | xaxaxaxaxaxaxaxaxaxaxaxaxaxaxaxaxaxaxaxaxaxaxaxaxaxaxaxaxaxa | 1.54E-06 | cg21393163 | NA | 2.30E-11 |  |  |  |  |  |  | 1 | 1 |  |  | 1 |  |  |  |
| cg08709672 | cg08709672 | AVPR1B | 1.93E-06 | cg08709672 | AVPR1B | 4.75E-25 |  |  |  |  |  |  | 1 | 1 |  |  | 1 |  |  |  |
| cg12547807 | cg12547807 | xaxaxaxaxaxaxaxaxaxaxaxaxaxaxaxaxaxaxaxaxaxaxaxaxaxaxaxaxaxa | 6.52E-06 | cg12547807 | NA | 2.57E-11 |  |  |  |  |  |  | 1 | 1 |  |  | 1 |  |  |  |
| cg02583484 | cg02583484 | HNRNPA1 | 7.10E-06 | cg02583484 | HNRNPA1 | 3.13E-15 |  |  |  |  |  |  | 1 | 1 |  |  | 1 |  |  |  |
| cg23771366 | cg23771366 | PRSS23 | 7.62E-06 | cg23771366 | PRSS23 | 1.09E-21 |  |  |  |  |  |  | 1 | 1 |  |  | 1 |  |  |  |
| cg21611682 | cg21611682 | LRP5 | 1.09E-04 | cg21611682 | LRP5 | 5.51E-33 |  |  |  |  |  |  | 1 | 1 |  |  | 1 |  |  |  |
| cg17619755 | cg17619755 | VARS | 2.64E-05 |  |  |  |  |  |  |  | 1 |  |  | 1 |  |  |  |  |  |  |
| cg24540678 | cg24540678 | xaxaxaxaxaxaxaxaxaxaxaxaxaxaxaxaxaxaxaxaxaxaxaxaxaxaxaxaxaxa | 2.30E-03 | cg24540678 | NA | 4.63E-11 |  |  |  |  |  |  | 1 | 1 |  |  | 1 |  |  |  |
| cg12513616 | cg12513616 | xaxaxaxaxaxaxaxaxaxaxaxaxaxaxaxaxaxaxaxaxaxaxaxaxaxaxaxaxaxa | 2.88E-03 | cg12513616 | NA | 8.90E-21 |  |  |  |  |  |  | 1 | 1 |  |  | 1 |  |  |  |
| cg07381806 | cg07381806 | MOBKL2A | 3.46E-03 | cg07381806 | MOBKL2A | 8.24E-12 |  |  |  |  |  |  | 1 | 1 |  |  | 1 |  |  |  |
| cg13039251 | cg13039251 | PDZD2 | 3.81E-04 |  |  |  |  |  |  |  | 1 |  |  | 1 |  |  |  |  |  |  |
| cg18146737 | cg18146737 | GFI1 | 4.79E-03 | cg18146737 | GFI1 | 1.77E-10 |  |  |  |  |  |  | 1 | 1 |  |  | 1 |  |  |  |
| cg10919522 | cg10919522 | C14orf43 | 6.20E-03 | cg10919522 | C14orf43 | 9.60E-09 |  |  |  |  |  |  | 1 | 1 |  |  | 1 |  |  |  |
| cg15417641 | cg15417641 | CACNA1D | 1.25E-02 |  |  |  |  |  |  |  | 1 |  |  | 1 |  |  |  |  |  |  |
| cg18316974 | cg18316974 | GFI1 | 1.60E-02 | cg18316974 | GFI1 | 6.27E-13 |  |  |  |  |  |  | 1 | 1 |  |  | 1 |  |  |  |
| cg23110422 | cg23110422 | ETS2 | 1.72E-02 | cg23110422 | ETS2 | 5.45E-15 |  |  |  |  |  |  | 1 | 1 |  |  | 1 |  |  |  |
| cg02532700 | cg02532700 | NCF4 | 1.88E-02 | cg02532700 | NCF4 | 2.22E-11 |  |  |  |  |  |  | 1 | 1 |  |  | 1 |  |  |  |
| cg19713429 | cg19713429 | CAPZB | 1.90E-02 | cg19713429 | CAPZB | 1.46E-09 |  |  |  |  |  |  | 1 | 1 |  |  | 1 |  |  |  |
| cg17924476 | cg17924476 | AHRR | 2.67E-02 |  |  |  |  |  |  |  | 1 |  |  | 1 |  |  |  |  |  |  |
| cg27312979 | cg27312979 | SORBS1 | 2.74E-02 |  |  |  |  |  |  |  | 1 |  |  | 1 |  |  |  |  |  |  |
| cg01127300 | cg01127300 | xaxaxaxaxaxaxaxaxaxaxaxaxaxaxaxaxaxaxaxaxaxaxaxaxaxaxaxaxa | 3.08E-02 | cg01127300 | NA | 1.28E-15 |  |  |  |  |  |  | 1 | 1 |  |  | 1 |  |  |  |
| cg02186444 | cg02186444 | ARMC7 | 4.67E-02 |  |  |  |  |  |  |  | 1 |  |  | 1 |  |  |  |  |  |  |
| cg13976502 | cg13976502 | C14orf43 | 4.67E-02 | cg13976502 | C14orf43 | 3.90E-12 |  |  |  |  |  |  | 1 | 1 |  |  | 1 |  |  |  |
| cg26242531 | cg26242531 | ZFYVE21 | 1.37E-01 |  |  |  |  |  |  |  | 1 |  |  | 1 |  |  |  |  |  |  |
| cg03234777 | cg03234777 | AMICA1 | 5.16E-01 |  |  |  |  |  |  |  | 1 |  |  | 1 |  |  |  |  |  |  |
| cg15159987 | cg15159987 | CPAMD8 | 6.39E-01 | cg15159987 | CPAMD8 | 8.38E-15 |  |  |  |  |  |  | 1 | 1 |  |  | 1 |  |  |  |
| cg03489965 | cg03489965 | LOC390594 | 9.52E-01 |  |  |  |  |  |  |  | 1 |  |  | 1 |  |  |  |  |  |  |
| cg26271591 | cg26271591 | NFE2L2 | 1.75E+00 | cg26271591 | NFE2L2 | 2.81E-12 |  |  |  |  |  |  | 1 | 1 |  |  | 1 |  |  |  |
| cg05875421 | cg05875421 | GPR68 | 2.15E+00 |  |  |  |  |  |  |  | 1 |  |  | 1 |  |  |  |  |  |  |
| cg24090911 | cg24090911 | AHRR | 3.41E+00 | cg24090911 | AHRR | 1.70E-19 |  |  |  |  |  |  | 1 | 1 |  |  | 1 |  |  |  |
| cg24049493 | cg24049493 | HIVEP3 | 3.73E+00 |  |  |  |  |  |  |  | 1 |  |  | 1 |  |  |  |  |  |  |
| cg15693572 | cg15693572 | xaxaxaxaxaxaxaxaxaxaxaxaxaxaxaxaxaxaxaxaxaxaxaxaxaxaxaxa | 3.84E+00 |  |  |  |  |  |  |  | 1 |  |  | 1 |  |  |  |  |  |  |
| cg19589396 | cg19589396 | xaxaxaxaxaxaxaxaxaxaxaxaxaxaxaxaxaxaxaxaxaxaxaxaxaxaxaxa | 4.23E+00 | cg19589396 | NA | 5.08E-13 |  |  |  |  |  |  | 1 | 1 |  |  | 1 |  |  |  |
| cg04180046 | cg04180046 | MYO1G | 5.92E+00 |  |  |  |  |  |  |  | 1 |  |  | 1 |  |  |  |  |  |  |
| cg23126342 | cg23126342 | PCDH9 | 7.16E+00 |  |  |  |  |  |  |  | 1 |  |  | 1 |  |  |  |  |  |  |
| cg16201146 | cg16201146 | xaxaxaxaxaxaxaxaxaxaxaxaxaxaxaxaxaxaxaxaxaxaxaxaxaxaxaxa | 7.91E-01 | cg16201146 | NA | 1.47E-13 |  |  |  |  |  |  | 1 | 1 |  |  | 1 |  |  |  |
| cg00073090 | cg00073090 | xaxaxaxaxaxaxaxaxaxaxaxaxaxaxaxaxaxaxaxaxaxaxaxaxaxaxaxa | 9.10E+00 | cg00073090 | NA | 2.08E-21 |  |  |  |  |  |  | 1 | 1 |  |  | 1 |  |  |  |
| cg13518625 | cg13518625 | xaxaxaxaxaxaxaxaxaxaxaxaxaxaxaxaxaxaxaxaxaxaxaxaxaxaxaxa | 1.15E+01 | cg13518625 | NA | 2.63E-09 |  |  |  |  |  |  | 1 | 1 |  |  | 1 |  |  |  |
| cg21121843 | cg21121843 | HTT | 1.79E+00 | cg21121843 | HTT | 4.85E-10 |  |  |  |  |  |  | 1 | 1 |  |  | 1 |  |  |  |
| cg19719391 | cg19719391 | xaxaxaxaxaxaxaxaxaxaxaxaxaxaxaxaxaxaxaxaxaxaxaxaxaxaxaxa | 2.32E+00 |  |  |  |  |  |  |  | 1 |  |  | 1 |  |  |  |  |  |  |
| cg00310412 | cg00310412 | SEMA7A | 2.34E+01 | cg00310412 | SEMA7A | 2.97E-15 |  |  |  |  |  |  | 1 | 1 |  |  | 1 |  |  |  |
| cg02451831 | cg02451831 | KIAA0087 | 2.79E+01 | cg02451831 | KIAA0087 | 1.37E-27 |  |  |  |  |  |  | 1 | 1 |  |  | 1 |  |  |  |
| cg15022400 | cg15022400 | TRIM69 | 3.36E+01 |  |  |  |  |  |  |  | 1 |  |  | 1 |  |  |  |  |  |  |
| cg11902728 | cg11902728 | MAG | 4.05E+00 |  |  |  |  |  |  |  | 1 |  |  | 1 |  |  |  |  |  |  |
| cg08884752 | cg08884752 | SKI | 4.43E+01 |  |  |  |  |  |  |  | 1 |  |  | 1 |  |  |  |  |  |  |
| cg18335991 | cg18335991 | SEMA7A | 4.90E+01 |  |  |  |  |  |  |  | 1 |  |  | 1 |  |  |  |  |  |  |
| cg22539182 | cg22539182 | xaxaxaxaxaxaxaxaxaxaxaxaxaxaxaxaxaxaxaxaxaxaxaxaxaxaxa | 7.40E+01 |  |  |  |  |  |  |  | 1 |  |  | 1 |  |  |  |  |  |  |
| cg07123182 | cg07123182 | KCNQ1OT1 | 8.64E+01 | cg07123182 | KCNQ1OT1 | 4.85E-15 |  |  |  |  |  |  | 1 | 1 |  |  | 1 |  |  |  |
| cg19089201 | cg19089201 | MYO1G | 9.26E+01 |  |  |  |  |  |  |  | 1 |  |  | 1 |  |  |  |  |  |  |
| cg03547355 | cg03547355 | xaxaxaxaxaxaxaxaxaxaxaxaxaxaxaxaxaxaxaxaxaxaxaxaxaxaxa | 1.06E+02 | cg03547355 | NA | 3.10E-10 |  |  |  |  |  |  | 1 | 1 |  |  | 1 |  |  |  |
| cg10807309 | cg10807309 | VARS | 1.25E+02 |  |  |  |  |  |  |  | 1 |  |  | 1 |  |  |  |  |  |  |
| cg19713851 | cg19713851 | ALPP | 1.28E+02 | cg19713851 | ALPP | 4.64E-08 |  |  |  |  |  |  | 1 | 1 |  |  | 1 |  |  |  |
| cg18642234 | cg18642234 | GPX1 | 1.44E+02 | cg18642234 | GPX1 | 6.82E-12 |  |  |  |  |  |  | 1 | 1 |  |  | 1 |  |  |  |
| cg00336149 | cg00336149 | CACNA1D | 1.57E+02 |  |  |  |  |  |  |  | 1 |  |  | 1 |  |  |  |  |  |  |
| cg00336149 | cg00336149 | CACNA1D | 1.57E+02 |  |  |  |  |  |  |  | 1 |  |  | 1 |  |  |  |  |  |  |
| cg14580211 | cg14580211 | C5orf62 | 2.19E+02 | cg14580211 | C5orf62 | 2.33E-23 |  |  |  |  |  |  | 1 | 1 |  |  | 1 |  |  |  |
| cg01208318 | cg01208318 | xaxaxaxaxaxaxaxaxaxaxaxaxaxaxaxaxaxaxaxaxaxaxaxaxaxaxa | 2.54E+02 | cg01208318 | NA | 6.19E-09 |  |  |  |  |  |  | 1 | 1 |  |  | 1 |  |  |  |
| cg25809905 | cg25809905 | ITGA2B | 2.74E+02 |  |  |  |  |  |  |  | 1 |  |  | 1 |  |  |  |  |  |  |
| cg14624207 | cg14624207 | LRP5 | 2.94E+01 | cg14624207 | LRP5 | 6.54E-14 |  |  |  |  |  |  | 1 | 1 |  |  | 1 |  |  |  |
| cg07465627 | cg07465627 | STXBP4 | 3.67E+02 | cg07465627 | STXBP4 | 9.09E-10 |  |  |  |  |  |  | 1 | 1 |  |  | 1 |  |  |  |
| cg17372101 | cg17372101 | CNTNAP2 | 4.17E+01 | cg17372101 | CNTNAP2 | 6.32E-08 |  |  |  |  |  |  | 1 | 1 |  |  | 1 |  |  |  |
| cg04039799 | cg04039799 | NAV2 | 5.24E+02 | cg04039799 | NAV2 | 3.49E-09 |  |  |  |  |  |  | 1 | 1 |  |  | 1 |  |  |  |
| cg16611234 | cg16611234 | xaxaxaxaxaxaxaxaxaxaxaxaxaxaxaxaxaxaxaxaxaxaxaxaxaxa | 8.31E+02 | cg16611234 | NA | 3.60E-13 |  |  |  |  |  |  | 1 | 1 |  |  | 1 |  |  |  |
| cg11152412 | cg11152412 | EDC3 | 1.37E+03 | cg11152412 | EDC3 | 5.48E-09 |  |  |  |  |  |  | 1 | 1 |  |  | 1 |  |  |  |
| cg03373393 | cg03373393 | HAP1 | 1.38E+03 |  |  |  |  |  |  |  | 1 |  |  | 1 |  |  |  |  |  |  |
| cg23667432 | cg23667432 | ALPP | 1.55E+03 | cg23667432 | ALPP | 2.82E-13 |  |  |  |  |  |  | 1 | 1 |  |  | 1 |  |  |  |
| cg03440944 | cg03440944 | C7orf40 | 1.68E+02 | cg03440944 | C7orf40 | 1.32E-08 |  |  |  |  |  |  | 1 | 1 |  |  | 1 |  |  |  |
| cg06885459 | cg06885459 | MCF2L | 2.09E+03 |  |  |  |  |  |  |  | 1 |  |  | 1 |  |  |  |  |  |  |
| cg16786458 | cg16786458 | PPARGC1B | 2.79E+03 |  |  |  |  |  |  |  | 1 |  |  | 1 |  |  |  |  |  |  |
| cg06819357 | cg06819357 | TECPR2 | 4.81E+02 |  |  |  |  |  |  |  | 1 |  |  | 1 |  |  |  |  |  |  |
| cg22635096 | cg22635096 | ADARB1 | 5.81E+03 |  |  |  |  |  |  |  | 1 |  |  | 1 |  |  |  |  |  |  |
| cg03188382 | cg03188382 | ALPP | 6.73E+03 |  |  |  |  |  |  |  | 1 |  |  | 1 |  |  |  |  |  |  |
| cg21913886 | cg21913886 | TMEM51 | 7.26E+03 | cg21913886 | TMEM51 | 9.58E-09 |  |  |  |  |  |  | 1 | 1 |  |  | 1 |  |  |  |
| cg01692968 | cg01692968 | xaxaxaxaxaxaxaxaxaxaxaxaxaxaxaxaxaxaxaxaxaxaxaxaxaxa | 7.30E+03 | cg01692968 | NA | 1.36E-26 |  |  |  |  |  |  | 1 | 1 |  |  | 1 |  |  |  |
| cg16255816 | cg16255816 | HAP1 | 9.19E+03 |  |  |  |  |  |  |  | 1 |  |  | 1 |  |  |  |  |  |  |
| cg23090529 | cg23090529 | xaxaxaxaxaxaxaxaxaxaxaxaxaxaxaxaxaxaxaxaxaxaxaxaxaxa | 9.90E+03 |  |  |  |  |  |  |  | 1 |  |  | 1 |  |  |  |  |  |  |
| cg21140898 | cg21140898 | xaxaxaxaxaxaxaxaxaxaxaxaxaxaxaxaxaxaxaxaxaxaxaxaxaxa | 1.08E+04 | cg21140898 | NA | 2.52E-08 |  |  |  |  |  |  | 1 | 1 |  |  | 1 |  |  |  |
| cg21188533 | cg21188533 | CACNA1D | 1.12E+04 |  |  |  |  |  |  |  | 1 |  |  | 1 |  |  |  |  |  |  |
| cg00931843 | cg00931843 | TIAM2 | 1.69E+04 | cg00931843 | TIAM2 | 7.68E-08 |  |  |  |  |  |  | 1 | 1 |  |  | 1 |  |  |  |
| cg06459104 | cg06459104 | EPB41L3 | 1.78E+04 |  |  |  |  |  |  |  | 1 |  |  | 1 |  |  |  |  |  |  |
| cg13038618 | cg13038618 | xaxaxaxaxaxaxaxaxaxaxaxaxaxaxaxaxaxaxaxaxaxaxaxaxa | 1.86E+04 | cg13038618 | NA | 1.69E-12 |  |  |  |  |  |  | 1 | 1 |  |  | 1 |  |  |  |
| cg11730703 | cg11730703 | INF2 | 2.04E+04 |  |  |  |  |  |  |  | 1 |  |  | 1 |  |  |  |  |  |  |
| cg13787850 | cg13787850 | xaxaxaxaxaxaxaxaxaxaxaxaxaxaxaxaxaxaxaxaxaxaxaxaxa | 2.14E+03 |  |  |  |  |  |  |  | 1 |  |  | 1 |  |  |  |  |  |  |
| cg11207515 | cg11207515 | CNTNAP2 | 2.53E+04 | cg11207515 | CNTNAP2 | 2.74E-16 |  |  |  |  |  |  | 1 | 1 |  |  | 1 |  |  |  |
| cg24556382 | cg24556382 | GALNT7 | 2.64E+04 | cg24556382 | GALNT7 | 8.78E-10 |  |  |  |  |  |  | 1 | 1 |  |  | 1 |  |  |  |
| cg09469355 | cg09469355 | SKI | 3.34E+04 | cg09469355 | SKI | 2.00E-10 |  |  |  |  |  |  | 1 | 1 |  |  | 1 |  |  |  |
| cg11701312 | cg11701312 | RPS5 | 3.40E+03 |  |  |  |  |  |  |  | 1 |  |  | 1 |  |  |  |  |  |  |
| cg04956244 | cg04956244 | RARA | 3.44E+04 |  |  |  |  |  |  |  | 1 |  |  | 1 |  |  |  |  |  |  |
| cg01744331 | cg01744331 | KCNQ1OT1 | 3.51E+04 | cg01744331 | KCNQ1OT1 | 3.77E-08 |  |  |  |  |  |  | 1 | 1 |  |  | 1 |  |  |  |
| cg17487894 | cg17487894 | RASA3 | 4.72E+04 |  |  |  |  |  |  |  | 1 |  |  | 1 |  |  |  |  |  |  |
| cg25197194 | cg25197194 | CCDC48 | 5.71E+04 |  |  |  |  |  |  |  | 1 |  |  | 1 |  |  |  |  |  |  |
| cg01207684 | cg01207684 | ADCY9 | 6.29E+04 |  |  |  |  |  |  |  | 1 |  |  | 1 |  |  |  |  |  |  |
| cg25953130 | cg25953130 | ARID5B | 6.34E+04 | cg25953130 | ARID5B | 6.52E-09 |  |  |  |  |  |  | 1 | 1 |  |  | 1 |  |  |  |
| cg16556677 | cg16556677 | KCNQ1OT1 | 6.58E+04 | cg16556677 | KCNQ1OT1 | 1.71E-13 |  |  |  |  |  |  | 1 | 1 |  |  | 1 |  |  |  |
| cg00911794 | cg00911794 | HIC1 | 6.84E+04 |  |  |  |  |  |  |  | 1 |  |  | 1 |  |  |  |  |  |  |
| cg00921574 | cg00921574 | INTS1 | 8.71E+04 |  |  |  |  |  |  |  | 1 |  |  | 1 |  |  |  |  |  |  |
| cg20295214 | cg20295214 | AVPR1B | 1.13E+05 | cg20295214 | AVPR1B | 2.44E-22 |  |  |  |  |  |  | 1 | 1 |  |  | 1 |  |  |  |
| cg22649124 | cg22649124 | LGALS7B | 1.15E+05 |  |  |  |  |  |  |  | 1 |  |  | 1 |  |  |  |  |  |  |
| cg08972170 | cg08972170 | C7orf41 | 1.53E+05 |  |  |  |  |  |  |  | 1 |  |  | 1 |  |  |  |  |  |  |
| cg01901332 | cg01901332 | ARRB1 | 1.70E+05 | cg01901332 | ARRB1 | 2.97E-26 |  |  |  |  |  |  | 1 | 1 |  |  | 1 |  |  |  |
| cg05284742 | cg05284742 | ITPK1 | 3.57E+05 | cg05284742 | ITPK1 | 1.03E-29 |  |  |  |  |  |  | 1 | 1 |  |  | 1 |  |  |  |
| cg11231349 | cg11231349 | NOS1AP | 3.94E+05 | cg11231349 | NOS1AP | 4.97E-14 |  |  |  |  |  |  | 1 | 1 |  |  | 1 |  |  |  |
| cg17024919 | cg17024919 | ZNF385D | 4.06E+04 |  |  |  |  |  |  |  | 1 |  |  | 1 |  |  |  |  |  |  |
| cg26361535 | cg26361535 | ZC3H3 | 4.14E+04 | cg26361535 | ZC3H3 | 4.62E-16 |  |  |  |  |  |  | 1 | 1 |  |  | 1 |  |  |  |
| cg25421530 | cg25421530 | SORBS1 | 4.22E+05 |  |  |  |  |  |  |  | 1 |  |  | 1 |  |  |  |  |  |  |
| cg19254163 | cg19254163 | GPR44 | 4.82E+05 | cg19254163 | GPR44 | 2.31E-13 |  |  |  |  |  |  | 1 | 1 |  |  | 1 |  |  |  |
| cg07251887 | cg07251887 | LOC100130933 | 5.61E+05 | cg07251887 | LOC100130933 | 4.93E-20 |  |  |  |  |  |  | 1 | 1 |  |  | 1 |  |  |  |
| cg09662411 | cg09662411 | GFI1 | 8.01E+05 | cg09662411 | GFI1 | 2.52E-14 |  |  |  |  |  |  | 1 | 1 |  |  | 1 |  |  |  |
| cg15187398 | cg15187398 | MOBKL2A | 8.51E+05 | cg15187398 | MOBKL2A | 3.96E-11 |  |  |  |  |  |  | 1 | 1 |  |  | 1 |  |  |  |
| cg08606254 | cg08606254 | AHRR | 9.25E+05 |  |  |  |  |  |  |  | 1 |  |  | 1 |  |  |  |  |  |  |
| cg15474579 | cg15474579 | CDKN1A | 9.30E+05 | cg15474579 | CDKN1A | 7.78E-14 |  |  |  |  |  |  | 1 | 1 |  |  | 1 |  |  |  |
| cg23681440 | cg23681440 | xaxaxaxaxaxaxaxaxaxaxaxaxaxaxaxaxaxaxaxaxaxaxa | 1.12E+06 | cg23681440 | NA | 1.02E-10 |  |  |  |  |  |  | 1 | 1 |  |  | 1 |  |  |  |
| cg19717773 | cg19717773 | GNA12 | 1.15E+05 | cg19717773 | GNA12 | 1.14E-10 |  |  |  |  |  |  | 1 | 1 |  |  | 1 |  |  |  |
| cg25491122 | cg25491122 | PCDH9 | 1.49E+06 |  |  |  |  |  |  |  | 1 |  |  | 1 |  |  |  |  |  |  |
| cg21280392 | cg21280392 | PHOSPHO1 | 1.56E+06 |  |  |  |  |  |  |  | 1 |  |  | 1 |  |  |  |  |  |  |
| cg01882991 | cg01882991 | xaxaxaxaxaxaxaxaxaxaxaxaxaxaxaxaxaxaxaxaxaxaxa | 1.74E+06 |  |  |  |  |  |  |  | 1 |  |  | 1 |  |  |  |  |  |  |
| cg25305703 | cg25305703 | xaxaxaxaxaxaxaxaxaxaxaxaxaxaxaxaxaxaxaxaxaxaxa | 2.09E+06 | cg25305703 | NA | 4.52E-20 |  |  |  |  |  |  | 1 | 1 |  |  | 1 |  |  |  |
| cg00835193 | cg00835193 | LINGO3 | 2.44E+06 |  |  |  |  |  |  |  | 1 |  |  | 1 |  |  |  |  |  |  |
| cg15542713 | cg15542713 | HIVEP3 | 2.54E+06 |  |  |  |  |  |  |  | 1 |  |  | 1 |  |  |  |  |  |  |
| cg06595162 | cg06595162 | NCRNA00114 | 2.65E+06 | cg06595162 | NCRNA00114 | 1.14E-10 |  |  |  |  |  |  | 1 | 1 |  |  | 1 |  |  |  |
| cg26908328 | cg26908328 | SERINC5 | 2.73E+05 |  |  |  |  |  |  |  | 1 |  |  | 1 |  |  |  |  |  |  |
| cg19406367 | cg19406367 | SGIP1 | 2.79E+06 |  |  |  |  |  |  |  | 1 |  |  | 1 |  |  |  |  |  |  |
| cg08202836 | cg08202836 | LRRC33 | 3.85E+06 |  |  |  |  |  |  |  | 1 |  |  | 1 |  |  |  |  |  |  |
| cg23621097 | cg23621097 | HIC1 | 4.23E+05 |  |  |  |  |  |  |  | 1 |  |  | 1 |  |  |  |  |  |  |
| cg09197783 | cg09197783 | SLC43A3 | 5.23E+05 |  |  |  |  |  |  |  | 1 |  |  | 1 |  |  |  |  |  |  |
| cg00871610 | cg00871610 | MIR802 | 5.46E+06 |  |  |  |  |  |  |  | 1 |  |  | 1 |  |  |  |  |  |  |
| cg09858022 | cg09858022 | RARA | 5.46E+06 |  |  |  |  |  |  |  | 1 |  |  | 1 |  |  |  |  |  |  |
| cg13910681 | cg13910681 | FAM102A | 5.92E+06 |  |  |  |  |  |  |  | 1 |  |  | 1 |  |  |  |  |  |  |
| cg17819085 | cg17819085 | xaxaxaxaxaxaxaxaxaxaxaxaxaxaxaxaxaxaxaxaxaxa | 6.87E+06 |  |  |  |  |  |  |  | 1 |  |  | 1 |  |  |  |  |  |  |
| cg26282236 | cg26282236 | RAD52 | 8.24E+06 |  |  |  |  |  |  |  | 1 |  |  | 1 |  |  |  |  |  |  |
| cg04158018 | cg04158018 | NFE2 | 8.40E+06 | cg04158018 | NFE2 | 2.42E-09 |  |  |  |  |  |  | 1 | 1 |  |  | 1 |  |  |  |
| cg12276019 | cg12276019 | XKR6 | 8.75E+06 |  |  |  |  |  |  |  | 1 |  |  | 1 |  |  |  |  |  |  |
| cg03155159 | cg03155159 | xaxaxaxaxaxaxaxaxaxaxaxaxaxaxaxaxaxaxaxaxaxa | 9.31E+06 |  |  |  |  |  |  |  | 1 |  |  | 1 |  |  |  |  |  |  |
| cg00007076 |  |  |  | cg00007076 | RRS1 | 4.76E-10 |  |  |  |  |  | 1 |  |  |  |  | 1 |  |  |  |
| cg00024404 |  |  |  | cg00024404 | SERINC5 | 2.43E-08 |  |  |  |  |  | 1 |  |  |  |  | 1 |  |  |  |
| cg00063111 |  |  |  | cg00063111 | SNORA6 | 5.16E-08 |  |  |  |  |  | 1 |  |  |  |  | 1 |  |  |  |
| cg00187059 |  |  |  | cg00187059 | DAXX | 5.44E-08 |  |  |  |  |  | 1 |  |  |  |  | 1 |  |  |  |
| cg00207731 |  |  |  | cg00207731 | FURIN | 7.06E-08 |  |  |  |  |  | 1 |  |  |  |  | 1 |  |  |  |
| cg00214171 |  |  |  | cg00214171 | RECQL5 | 4.79E-09 |  |  |  |  |  | 1 |  |  |  |  | 1 |  |  |  |
| cg00295485 |  |  |  | cg00295485 | UXS1 | 9.76E-16 |  |  |  |  |  | 1 |  |  |  |  | 1 |  |  |  |
| cg00324097 |  |  |  | cg00324097 |  | 5.50E-09 |  |  |  |  |  | 1 |  |  |  |  | 1 |  |  |  |
| cg00489660 |  |  |  | cg00489660 | TNKS1BP1 | 1.51E-08 |  |  |  |  |  | 1 |  |  |  |  | 1 |  |  |  |
| cg00534655 |  |  |  | cg00534655 | PURA | 9.06E-08 |  |  |  |  |  | 1 |  |  |  |  | 1 |  |  |  |
| cg00540464 |  |  |  | cg00540464 | NA | 7.86E-09 |  |  |  |  |  | 1 |  |  |  |  | 1 |  |  |  |
| cg00554421 |  |  |  | cg00554421 | INTS1 | 8.57E-10 |  |  |  |  |  | 1 |  |  |  |  | 1 |  |  |  |
| cg00566320 |  |  |  | cg00566320 | NA | 1.04E-07 |  |  |  |  |  | 1 |  |  |  |  | 1 |  |  |  |
| cg00639656 |  |  |  | cg00639656 | SNORD42A | 5.25E-08 |  |  |  |  |  | 1 |  |  |  |  | 1 |  |  |  |
| cg00830621 |  |  |  | cg00830621 | RPS15A | 4.31E-08 |  |  |  |  |  | 1 |  |  |  |  | 1 |  |  |  |
| cg01005506 |  |  |  | cg01005506 | ADO | 2.27E-08 |  |  |  |  |  | 1 |  |  |  |  | 1 |  |  |  |
| cg01017464 |  |  |  | cg01017464 | SNORD58A | 3.96E-09 |  |  |  |  |  | 1 |  |  |  |  | 1 |  |  |  |
| cg01205831 |  |  |  | cg01205831 | DPM2 | 6.47E-08 |  |  |  |  |  | 1 |  |  |  |  | 1 |  |  |  |
| cg01272202 |  |  |  | cg01272202 | MCC | 3.46E-08 |  |  |  |  |  | 1 |  |  |  |  | 1 |  |  |  |
| cg01293143 |  |  |  | cg01293143 | TCEA2 | 7.80E-09 |  |  |  |  |  | 1 |  |  |  |  | 1 |  |  |  |
| cg01442064 |  |  |  | cg01442064 | EVC | 1.10E-08 |  |  |  |  |  | 1 |  |  |  |  | 1 |  |  |  |
| cg01447748 |  |  |  | cg01447748 | EPS15 | 1.57E-08 |  |  |  |  |  | 1 |  |  |  |  | 1 |  |  |  |
| cg01478234 |  |  |  | cg01478234 | BTBD11 | 1.10E-09 |  |  |  |  |  | 1 |  |  |  |  | 1 |  |  |  |
| cg01513913 |  |  |  | cg01513913 | NA | 4.15E-09 |  |  |  |  |  | 1 |  |  |  |  | 1 |  |  |  |
| cg01554474 |  |  |  | cg01554474 | RAG1AP1 | 4.00E-12 |  |  |  |  |  | 1 |  |  |  |  | 1 |  |  |  |
| cg01588224 |  |  |  | cg01588224 | JTB | 3.15E-11 |  |  |  |  |  | 1 |  |  |  |  | 1 |  |  |  |
| cg01604883 |  |  |  | cg01604883 | NA | 6.94E-08 |  |  |  |  |  | 1 |  |  |  |  | 1 |  |  |  |
| cg01651915 |  |  |  | cg01651915 | NA | 2.91E-08 |  |  |  |  |  | 1 |  |  |  |  | 1 |  |  |  |
| cg01668352 |  |  |  | cg01668352 | SRGAP1 | 9.02E-09 |  |  |  |  |  | 1 |  |  |  |  | 1 |  |  |  |
| cg01693305 |  |  |  | cg01693305 | CAPZB | 2.31E-14 |  |  |  |  |  | 1 |  |  |  |  | 1 |  |  |  |
| cg01763916 |  |  |  | cg01763916 | SMAP2 | 2.22E-10 |  |  |  |  |  | 1 |  |  |  |  | 1 |  |  |  |
| cg01765406 |  |  |  | cg01765406 | NA | 6.80E-11 |  |  |  |  |  | 1 |  |  |  |  | 1 |  |  |  |
| cg01832549 |  |  |  | cg01832549 | CAPZB | 3.25E-10 |  |  |  |  |  | 1 |  |  |  |  | 1 |  |  |  |
| cg01839993 |  |  |  | cg01839993 | DDIT4 | 2.89E-09 |  |  |  |  |  | 1 |  |  |  |  | 1 |  |  |  |
| cg01873977 |  |  |  | cg01873977 | MTSS1 | 1.60E-10 |  |  |  |  |  | 1 |  |  |  |  | 1 |  |  |  |
| cg01919999 |  |  |  | cg01919999 | GNB2L1 | 1.69E-09 |  |  |  |  |  | 1 |  |  |  |  | 1 |  |  |  |
| cg01937809 |  |  |  | cg01937809 | ZC3H12A | 5.36E-08 |  |  |  |  |  | 1 |  |  |  |  | 1 |  |  |  |
| cg01972009 |  |  |  | cg01972009 | PITPNM1 | 3.48E-08 |  |  |  |  |  | 1 |  |  |  |  | 1 |  |  |  |
| cg01999701 |  |  |  | cg01999701 | NA | 5.42E-08 |  |  |  |  |  | 1 |  |  |  |  | 1 |  |  |  |
| cg02003272 |  |  |  | cg02003272 | NA | 6.32E-10 |  |  |  |  |  | 1 |  |  |  |  | 1 |  |  |  |
| cg02054431 |  |  |  | cg02054431 | SNORA38 | 6.64E-08 |  |  |  |  |  | 1 |  |  |  |  | 1 |  |  |  |
| cg02315870 |  |  |  | cg02315870 | HNRNPA2B1 | 9.24E-08 |  |  |  |  |  | 1 |  |  |  |  | 1 |  |  |  |
| cg02325250 |  |  |  | cg02325250 | CSF2 | 7.42E-08 |  |  |  |  |  | 1 |  |  |  |  | 1 |  |  |  |
| cg02385153 |  |  |  | cg02385153 | AHRR | 9.71E-08 |  |  |  |  |  | 1 |  |  |  |  | 1 |  |  |  |
| cg02417427 |  |  |  | cg02417427 | SERINC5 | 8.46E-08 |  |  |  |  |  | 1 |  |  |  |  | 1 |  |  |  |
| cg02743070 |  |  |  | cg02743070 | ZMIZ1 | 2.52E-10 |  |  |  |  |  | 1 |  |  |  |  | 1 |  |  |  |
| cg02767093 |  |  |  | cg02767093 | STK24 | 6.14E-09 |  |  |  |  |  | 1 |  |  |  |  | 1 |  |  |  |
| cg02810967 |  |  |  | cg02810967 | NCAPG | 2.00E-09 |  |  |  |  |  | 1 |  |  |  |  | 1 |  |  |  |
| cg02917867 |  |  |  | cg02917867 | NA | 9.36E-08 |  |  |  |  |  | 1 |  |  |  |  | 1 |  |  |  |
| cg02964434 |  |  |  | cg02964434 | NA | 5.43E-08 |  |  |  |  |  | 1 |  |  |  |  | 1 |  |  |  |
| cg03117379 |  |  |  | cg03117379 | SNORD17 | 9.06E-09 |  |  |  |  |  | 1 |  |  |  |  | 1 |  |  |  |
| cg03140521 |  |  |  | cg03140521 | GNG12 | 8.34E-09 |  |  |  |  |  | 1 |  |  |  |  | 1 |  |  |  |
| cg03147185 |  |  |  | cg03147185 | NCAPH | 7.30E-09 |  |  |  |  |  | 1 |  |  |  |  | 1 |  |  |  |
| cg03149958 |  |  |  | cg03149958 | NA | 1.64E-08 |  |  |  |  |  | 1 |  |  |  |  | 1 |  |  |  |
| cg03296761 |  |  |  | cg03296761 | MTP18 | 4.46E-08 |  |  |  |  |  | 1 |  |  |  |  | 1 |  |  |  |
| cg03340878 |  |  |  | cg03340878 | OR2B6 | 8.32E-08 |  |  |  |  |  | 1 |  |  |  |  | 1 |  |  |  |
| cg03358636 |  |  |  | cg03358636 | KIAA0226 | 2.05E-10 |  |  |  |  |  | 1 |  |  |  |  | 1 |  |  |  |
| cg03450842 |  |  |  | cg03450842 | ZMIZ1 | 1.96E-23 |  |  |  |  |  | 1 |  |  |  |  | 1 |  |  |  |
| cg03474926 |  |  |  | cg03474926 | RALGDS | 3.47E-08 |  |  |  |  |  | 1 |  |  |  |  | 1 |  |  |  |
| cg03519879 |  |  |  | cg03519879 | C14orf43 | 1.58E-11 |  |  |  |  |  | 1 |  |  |  |  | 1 |  |  |  |
| cg03604424 |  |  |  | cg03604424 | TRIO | 1.25E-08 |  |  |  |  |  | 1 |  |  |  |  | 1 |  |  |  |
| cg03655142 |  |  |  | cg03655142 | JTB | 9.94E-12 |  |  |  |  |  | 1 |  |  |  |  | 1 |  |  |  |
| cg03707168 |  |  |  | cg03707168 | PPP1R15A | 2.58E-19 |  |  |  |  |  | 1 |  |  |  |  | 1 |  |  |  |
| cg03785076 |  |  |  | cg03785076 | SNED1 | 3.11E-08 |  |  |  |  |  | 1 |  |  |  |  | 1 |  |  |  |
| cg03929796 |  |  |  | cg03929796 | ALAS1 | 2.18E-09 |  |  |  |  |  | 1 |  |  |  |  | 1 |  |  |  |
| cg03999941 |  |  |  | cg03999941 | NA | 6.40E-09 |  |  |  |  |  | 1 |  |  |  |  | 1 |  |  |  |
| cg04011474 |  |  |  | cg04011474 | NA | 1.47E-08 |  |  |  |  |  | 1 |  |  |  |  | 1 |  |  |  |
| cg04202338 |  |  |  | cg04202338 | RPL32 | 3.78E-10 |  |  |  |  |  | 1 |  |  |  |  | 1 |  |  |  |
| cg04388657 |  |  |  | cg04388657 | RPL35 | 1.92E-08 |  |  |  |  |  | 1 |  |  |  |  | 1 |  |  |  |
| cg04517079 |  |  |  | cg04517079 | FOXP4 | 1.26E-11 |  |  |  |  |  | 1 |  |  |  |  | 1 |  |  |  |
| cg04535902 |  |  |  | cg04535902 | GFI1 | 2.74E-08 |  |  |  |  |  | 1 |  |  |  |  | 1 |  |  |  |
| cg04551776 |  |  |  | cg04551776 | AHRR | 4.30E-20 |  |  |  |  |  | 1 |  |  |  |  | 1 |  |  |  |
| cg04759756 |  |  |  | cg04759756 | SLA2 | 2.46E-08 |  |  |  |  |  | 1 |  |  |  |  | 1 |  |  |  |
| cg04761231 |  |  |  | cg04761231 | RPL35 | 1.04E-08 |  |  |  |  |  | 1 |  |  |  |  | 1 |  |  |  |
| cg04813697 |  |  |  | cg04813697 | PIP4K2A | 4.75E-10 |  |  |  |  |  | 1 |  |  |  |  | 1 |  |  |  |
| cg04907244 |  |  |  | cg04907244 | SNORD93 | 1.45E-10 |  |  |  |  |  | 1 |  |  |  |  | 1 |  |  |  |
| cg04929932 |  |  |  | cg04929932 | NA | 8.76E-09 |  |  |  |  |  | 1 |  |  |  |  | 1 |  |  |  |
| cg04945608 |  |  |  | cg04945608 | TTPAL | 1.08E-07 |  |  |  |  |  | 1 |  |  |  |  | 1 |  |  |  |
| cg04962621 |  |  |  | cg04962621 | MGRN1 | 3.91E-08 |  |  |  |  |  | 1 |  |  |  |  | 1 |  |  |  |
| cg05051464 |  |  |  | cg05051464 | CAPZB | 1.73E-09 |  |  |  |  |  | 1 |  |  |  |  | 1 |  |  |  |
| cg05221370 |  |  |  | cg05221370 | LRRN3 | 5.31E-14 |  |  |  |  |  | 1 |  |  |  |  | 1 |  |  |  |
| cg05228408 |  |  |  | cg05228408 | CLCN6 | 4.00E-08 |  |  |  |  |  | 1 |  |  |  |  | 1 |  |  |  |
| cg05329352 |  |  |  | cg05329352 | ADRA2A | 1.65E-08 |  |  |  |  |  | 1 |  |  |  |  | 1 |  |  |  |
| cg05339037 |  |  |  | cg05339037 | NA | 2.25E-08 |  |  |  |  |  | 1 |  |  |  |  | 1 |  |  |  |
| cg05343105 |  |  |  | cg05343105 | KIF22 | 9.71E-08 |  |  |  |  |  | 1 |  |  |  |  | 1 |  |  |  |
| cg05397202 |  |  |  | cg05397202 | PUSL1 | 8.75E-08 |  |  |  |  |  | 1 |  |  |  |  | 1 |  |  |  |
| cg05460226 |  |  |  | cg05460226 | PIK3R5 | 5.37E-16 |  |  |  |  |  | 1 |  |  |  |  | 1 |  |  |  |
| cg05603985 |  |  |  | cg05603985 | SKI | 1.02E-07 |  |  |  |  |  | 1 |  |  |  |  | 1 |  |  |  |
| cg05616472 |  |  |  | cg05616472 | EHMT1 | 5.41E-08 |  |  |  |  |  | 1 |  |  |  |  | 1 |  |  |  |
| cg05655806 |  |  |  | cg05655806 | CD96 | 3.84E-09 |  |  |  |  |  | 1 |  |  |  |  | 1 |  |  |  |
| cg05677062 |  |  |  | cg05677062 | SETD8 | 1.58E-10 |  |  |  |  |  | 1 |  |  |  |  | 1 |  |  |  |
| cg05721773 |  |  |  | cg05721773 | FAIM3 | 9.57E-09 |  |  |  |  |  | 1 |  |  |  |  | 1 |  |  |  |
| cg05726935 |  |  |  | cg05726935 | AKT1 | 2.80E-10 |  |  |  |  |  | 1 |  |  |  |  | 1 |  |  |  |
| cg05789250 |  |  |  | cg05789250 | C6orf48 | 1.43E-08 |  |  |  |  |  | 1 |  |  |  |  | 1 |  |  |  |
| cg05802386 |  |  |  | cg05802386 | SLC2A1 | 7.19E-08 |  |  |  |  |  | 1 |  |  |  |  | 1 |  |  |  |
| cg05824218 |  |  |  | cg05824218 | RARA | 9.55E-11 |  |  |  |  |  | 1 |  |  |  |  | 1 |  |  |  |
| cg05886626 |  |  |  | cg05886626 | THBS1 | 5.52E-08 |  |  |  |  |  | 1 |  |  |  |  | 1 |  |  |  |
| cg05958351 |  |  |  | cg05958351 | SRRM2 | 2.66E-08 |  |  |  |  |  | 1 |  |  |  |  | 1 |  |  |  |
| cg06096184 |  |  |  | cg06096184 | LRIG1 | 5.38E-08 |  |  |  |  |  | 1 |  |  |  |  | 1 |  |  |  |
| cg06121808 |  |  |  | cg06121808 | SLC20A1 | 3.45E-08 |  |  |  |  |  | 1 |  |  |  |  | 1 |  |  |  |
| cg06154597 |  |  |  | cg06154597 | GAK | 2.41E-08 |  |  |  |  |  | 1 |  |  |  |  | 1 |  |  |  |
| cg06178669 |  |  |  | cg06178669 | NA | 1.89E-08 |  |  |  |  |  | 1 |  |  |  |  | 1 |  |  |  |
| cg06223834 |  |  |  | cg06223834 | ADCY9 | 8.00E-08 |  |  |  |  |  | 1 |  |  |  |  | 1 |  |  |  |
| cg06235438 |  |  |  | cg06235438 | ITGAL | 1.78E-22 |  |  |  |  |  | 1 |  |  |  |  | 1 |  |  |  |
| cg06294803 |  |  |  | cg06294803 | EIF4G1 | 1.75E-09 |  |  |  |  |  | 1 |  |  |  |  | 1 |  |  |  |
| cg06321596 |  |  |  | cg06321596 | XYLT1 | 1.24E-09 |  |  |  |  |  | 1 |  |  |  |  | 1 |  |  |  |
| cg06338710 |  |  |  | cg06338710 | GFI1 | 9.57E-09 |  |  |  |  |  | 1 |  |  |  |  | 1 |  |  |  |
| cg06457408 |  |  |  | cg06457408 | ARHGDIA | 2.95E-10 |  |  |  |  |  | 1 |  |  |  |  | 1 |  |  |  |
| cg06532880 |  |  |  | cg06532880 | PRELID1 | 1.81E-08 |  |  |  |  |  | 1 |  |  |  |  | 1 |  |  |  |
| cg06762457 |  |  |  | cg06762457 | ZC3H12D | 1.24E-08 |  |  |  |  |  | 1 |  |  |  |  | 1 |  |  |  |
| cg06890522 |  |  |  | cg06890522 | RNASEK | 9.47E-08 |  |  |  |  |  | 1 |  |  |  |  | 1 |  |  |  |
| cg07069636 |  |  |  | cg07069636 | NA | 2.08E-13 |  |  |  |  |  | 1 |  |  |  |  | 1 |  |  |  |
| cg07094298 |  |  |  | cg07094298 | TNIP2 | 1.16E-08 |  |  |  |  |  | 1 |  |  |  |  | 1 |  |  |  |
| cg07113006 |  |  |  | cg07113006 | RNF220 | 4.05E-09 |  |  |  |  |  | 1 |  |  |  |  | 1 |  |  |  |
| cg07151117 |  |  |  | cg07151117 | DUSP4 | 9.95E-09 |  |  |  |  |  | 1 |  |  |  |  | 1 |  |  |  |
| cg07180646 |  |  |  | cg07180646 | TMEM51 | 1.52E-10 |  |  |  |  |  | 1 |  |  |  |  | 1 |  |  |  |
| cg07202214 |  |  |  | cg07202214 | LRRC32 | 2.07E-12 |  |  |  |  |  | 1 |  |  |  |  | 1 |  |  |  |
| cg07265588 |  |  |  | cg07265588 | NA | 1.85E-09 |  |  |  |  |  | 1 |  |  |  |  | 1 |  |  |  |
| cg07512814 |  |  |  | cg07512814 | LDLR | 7.36E-08 |  |  |  |  |  | 1 |  |  |  |  | 1 |  |  |  |
| cg07805542 |  |  |  | cg07805542 | PIK3CD | 4.83E-09 |  |  |  |  |  | 1 |  |  |  |  | 1 |  |  |  |
| cg07945582 |  |  |  | cg07945582 | NFE2L3 | 9.63E-08 |  |  |  |  |  | 1 |  |  |  |  | 1 |  |  |  |
| cg07986378 |  |  |  | cg07986378 | ETV6 | 2.57E-18 |  |  |  |  |  | 1 |  |  |  |  | 1 |  |  |  |
| cg08101174 |  |  |  | cg08101174 | NA | 9.12E-10 |  |  |  |  |  | 1 |  |  |  |  | 1 |  |  |  |
| cg08149865 |  |  |  | cg08149865 | EPB49 | 1.01E-10 |  |  |  |  |  | 1 |  |  |  |  | 1 |  |  |  |
| cg08170227 |  |  |  | cg08170227 | ACTN1 | 8.66E-08 |  |  |  |  |  | 1 |  |  |  |  | 1 |  |  |  |
| cg08257009 |  |  |  | cg08257009 | NA | 7.58E-10 |  |  |  |  |  | 1 |  |  |  |  | 1 |  |  |  |
| cg08262002 |  |  |  | cg08262002 | LDB2 | 1.08E-07 |  |  |  |  |  | 1 |  |  |  |  | 1 |  |  |  |
| cg08354053 |  |  |  | cg08354053 | RHBDL3 | 9.26E-09 |  |  |  |  |  | 1 |  |  |  |  | 1 |  |  |  |
| cg08396193 |  |  |  | cg08396193 | HOXA7 | 3.73E-08 |  |  |  |  |  | 1 |  |  |  |  | 1 |  |  |  |
| cg08553327 |  |  |  | cg08553327 | TNF | 2.30E-11 |  |  |  |  |  | 1 |  |  |  |  | 1 |  |  |  |
| cg08666707 |  |  |  | cg08666707 | NA | 3.96E-08 |  |  |  |  |  | 1 |  |  |  |  | 1 |  |  |  |
| cg08682036 |  |  |  | cg08682036 | HNRNPF | 1.21E-08 |  |  |  |  |  | 1 |  |  |  |  | 1 |  |  |  |
| cg08737116 |  |  |  | cg08737116 | SLC22A15 | 8.82E-09 |  |  |  |  |  | 1 |  |  |  |  | 1 |  |  |  |
| cg08763102 |  |  |  | cg08763102 | HTT | 1.69E-09 |  |  |  |  |  | 1 |  |  |  |  | 1 |  |  |  |
| cg08866634 |  |  |  | cg08866634 | RNF44 | 5.89E-10 |  |  |  |  |  | 1 |  |  |  |  | 1 |  |  |  |
| cg08893087 |  |  |  | cg08893087 | FAM69A | 1.09E-07 |  |  |  |  |  | 1 |  |  |  |  | 1 |  |  |  |
| cg09022230 |  |  |  | cg09022230 | TNRC18 | 3.13E-10 |  |  |  |  |  | 1 |  |  |  |  | 1 |  |  |  |
| cg09069072 |  |  |  | cg09069072 | TMEM51 | 3.52E-08 |  |  |  |  |  | 1 |  |  |  |  | 1 |  |  |  |
| cg09084200 |  |  |  | cg09084200 | VPS26B | 9.10E-11 |  |  |  |  |  | 1 |  |  |  |  | 1 |  |  |  |
| cg09282085 |  |  |  | cg09282085 | RPL3 | 6.17E-08 |  |  |  |  |  | 1 |  |  |  |  | 1 |  |  |  |
| cg09465703 |  |  |  | cg09465703 | JMJD8 | 1.14E-08 |  |  |  |  |  | 1 |  |  |  |  | 1 |  |  |  |
| cg09479241 |  |  |  | cg09479241 | TLCD1 | 7.27E-08 |  |  |  |  |  | 1 |  |  |  |  | 1 |  |  |  |
| cg09578155 |  |  |  | cg09578155 | LRP5 | 7.02E-10 |  |  |  |  |  | 1 |  |  |  |  | 1 |  |  |  |
| cg09658497 |  |  |  | cg09658497 | GNA12 | 1.78E-09 |  |  |  |  |  | 1 |  |  |  |  | 1 |  |  |  |
| cg09931909 |  |  |  | cg09931909 | C6orf150 | 1.12E-07 |  |  |  |  |  | 1 |  |  |  |  | 1 |  |  |  |
| cg10062919 |  |  |  | cg10062919 | RARA | 4.67E-12 |  |  |  |  |  | 1 |  |  |  |  | 1 |  |  |  |
| cg10179300 |  |  |  | cg10179300 | TRIO | 5.82E-09 |  |  |  |  |  | 1 |  |  |  |  | 1 |  |  |  |
| cg10190813 |  |  |  | cg10190813 | HUS1 | 7.55E-09 |  |  |  |  |  | 1 |  |  |  |  | 1 |  |  |  |
| cg10237088 |  |  |  | cg10237088 | POFUT1 | 9.45E-08 |  |  |  |  |  | 1 |  |  |  |  | 1 |  |  |  |
| cg10361922 |  |  |  | cg10361922 | VPS25 | 2.12E-08 |  |  |  |  |  | 1 |  |  |  |  | 1 |  |  |  |
| cg10399789 |  |  |  | cg10399789 | GFI1 | 3.49E-10 |  |  |  |  |  | 1 |  |  |  |  | 1 |  |  |  |
| cg10420527 |  |  |  | cg10420527 | LRP5 | 1.05E-14 |  |  |  |  |  | 1 |  |  |  |  | 1 |  |  |  |
| cg10678215 |  |  |  | cg10678215 | HMHB1 | 1.59E-08 |  |  |  |  |  | 1 |  |  |  |  | 1 |  |  |  |
| cg10691866 |  |  |  | cg10691866 | TPST1 | 1.93E-09 |  |  |  |  |  | 1 |  |  |  |  | 1 |  |  |  |
| cg10750182 |  |  |  | cg10750182 | C10orf105 | 3.54E-19 |  |  |  |  |  | 1 |  |  |  |  | 1 |  |  |  |
| cg10788371 |  |  |  | cg10788371 | LRRC32 | 1.03E-08 |  |  |  |  |  | 1 |  |  |  |  | 1 |  |  |  |
| cg10790685 |  |  |  | cg10790685 | VIM | 6.80E-08 |  |  |  |  |  | 1 |  |  |  |  | 1 |  |  |  |
| cg10814005 |  |  |  | cg10814005 | GPR68 | 1.42E-08 |  |  |  |  |  | 1 |  |  |  |  | 1 |  |  |  |
| cg10858677 |  |  |  | cg10858677 | NA | 4.95E-08 |  |  |  |  |  | 1 |  |  |  |  | 1 |  |  |  |
| cg10965178 |  |  |  | cg10965178 | TIE1 | 6.31E-10 |  |  |  |  |  | 1 |  |  |  |  | 1 |  |  |  |
| cg11071448 |  |  |  | cg11071448 | SYT2 | 1.86E-12 |  |  |  |  |  | 1 |  |  |  |  | 1 |  |  |  |
| cg11082959 |  |  |  | cg11082959 | FASTK | 9.46E-10 |  |  |  |  |  | 1 |  |  |  |  | 1 |  |  |  |
| cg11094248 |  |  |  | cg11094248 | RARA | 1.26E-11 |  |  |  |  |  | 1 |  |  |  |  | 1 |  |  |  |
| cg11130692 |  |  |  | cg11130692 | EIF2C2 | 1.07E-07 |  |  |  |  |  | 1 |  |  |  |  | 1 |  |  |  |
| cg11314684 |  |  |  | cg11314684 | AKT3 | 2.06E-08 |  |  |  |  |  | 1 |  |  |  |  | 1 |  |  |  |
| cg11436113 |  |  |  | cg11436113 | NA | 1.31E-10 |  |  |  |  |  | 1 |  |  |  |  | 1 |  |  |  |
| cg11619216 |  |  |  | cg11619216 | LOC100130933 | 1.48E-13 |  |  |  |  |  | 1 |  |  |  |  | 1 |  |  |  |
| cg11621113 |  |  |  | cg11621113 | MORG1 | 5.08E-09 |  |  |  |  |  | 1 |  |  |  |  | 1 |  |  |  |
| cg11624345 |  |  |  | cg11624345 | KCNN4 | 4.65E-10 |  |  |  |  |  | 1 |  |  |  |  | 1 |  |  |  |
| cg11739148 |  |  |  | cg11739148 | KLF13 | 3.79E-11 |  |  |  |  |  | 1 |  |  |  |  | 1 |  |  |  |
| cg11794215 |  |  |  | cg11794215 | NA | 9.98E-08 |  |  |  |  |  | 1 |  |  |  |  | 1 |  |  |  |
| cg11927233 |  |  |  | cg11927233 | NPM1 | 1.32E-08 |  |  |  |  |  | 1 |  |  |  |  | 1 |  |  |  |
| cg12033822 |  |  |  | cg12033822 | SLC35C2 | 7.73E-08 |  |  |  |  |  | 1 |  |  |  |  | 1 |  |  |  |
| cg12147622 |  |  |  | cg12147622 | NA | 3.37E-10 |  |  |  |  |  | 1 |  |  |  |  | 1 |  |  |  |
| cg12303084 |  |  |  | cg12303084 | ZMYND8 | 4.09E-12 |  |  |  |  |  | 1 |  |  |  |  | 1 |  |  |  |
| cg12364755 |  |  |  | cg12364755 | PAFAH1B1 | 6.54E-08 |  |  |  |  |  | 1 |  |  |  |  | 1 |  |  |  |
| cg12406027 |  |  |  | cg12406027 | PAK4 | 9.58E-08 |  |  |  |  |  | 1 |  |  |  |  | 1 |  |  |  |
| cg12554573 |  |  |  | cg12554573 | PARP3 | 1.02E-07 |  |  |  |  |  | 1 |  |  |  |  | 1 |  |  |  |
| cg12593793 |  |  |  | cg12593793 | NA | 3.28E-10 |  |  |  |  |  | 1 |  |  |  |  | 1 |  |  |  |
| cg12655542 |  |  |  | cg12655542 | NA | 1.81E-08 |  |  |  |  |  | 1 |  |  |  |  | 1 |  |  |  |
| cg12678834 |  |  |  | cg12678834 | CXCR5 | 9.23E-10 |  |  |  |  |  | 1 |  |  |  |  | 1 |  |  |  |
| cg12761472 |  |  |  | cg12761472 | NA | 5.47E-09 |  |  |  |  |  | 1 |  |  |  |  | 1 |  |  |  |
| cg12873476 |  |  |  | cg12873476 | NA | 1.26E-08 |  |  |  |  |  | 1 |  |  |  |  | 1 |  |  |  |
| cg13009654 |  |  |  | cg13009654 | EGR1 | 1.94E-08 |  |  |  |  |  | 1 |  |  |  |  | 1 |  |  |  |
| cg13092108 |  |  |  | cg13092108 | RPS6KA1 | 1.47E-08 |  |  |  |  |  | 1 |  |  |  |  | 1 |  |  |  |
| cg13127741 |  |  |  | cg13127741 | COMMD7 | 1.73E-08 |  |  |  |  |  | 1 |  |  |  |  | 1 |  |  |  |
| cg13184736 |  |  |  | cg13184736 | GNG12 | 8.14E-13 |  |  |  |  |  | 1 |  |  |  |  | 1 |  |  |  |
| cg13256912 |  |  |  | cg13256912 | HRH1 | 5.04E-08 |  |  |  |  |  | 1 |  |  |  |  | 1 |  |  |  |
| cg13399816 |  |  |  | cg13399816 | GNG12 | 2.35E-08 |  |  |  |  |  | 1 |  |  |  |  | 1 |  |  |  |
| cg13500388 |  |  |  | cg13500388 | CBFB | 7.94E-16 |  |  |  |  |  | 1 |  |  |  |  | 1 |  |  |  |
| cg13541527 |  |  |  | cg13541527 | C6orf48 | 1.48E-08 |  |  |  |  |  | 1 |  |  |  |  | 1 |  |  |  |
| cg13543915 |  |  |  | cg13543915 | NA | 2.12E-10 |  |  |  |  |  | 1 |  |  |  |  | 1 |  |  |  |
| cg13583535 |  |  |  | cg13583535 | MBP | 1.83E-08 |  |  |  |  |  | 1 |  |  |  |  | 1 |  |  |  |
| cg13633560 |  |  |  | cg13633560 | LRRC32 | 6.22E-08 |  |  |  |  |  | 1 |  |  |  |  | 1 |  |  |  |
| cg13657200 |  |  |  | cg13657200 | DFNB31 | 1.00E-07 |  |  |  |  |  | 1 |  |  |  |  | 1 |  |  |  |
| cg13708645 |  |  |  | cg13708645 | KDM2B | 1.11E-07 |  |  |  |  |  | 1 |  |  |  |  | 1 |  |  |  |
| cg13745346 |  |  |  | cg13745346 | CBFA2T3 | 2.96E-08 |  |  |  |  |  | 1 |  |  |  |  | 1 |  |  |  |
| cg13784312 |  |  |  | cg13784312 | RAPGEF1 | 8.43E-09 |  |  |  |  |  | 1 |  |  |  |  | 1 |  |  |  |
| cg13832201 |  |  |  | cg13832201 | MAPK7 | 7.77E-09 |  |  |  |  |  | 1 |  |  |  |  | 1 |  |  |  |
| cg13916835 |  |  |  | cg13916835 | SMG6 | 1.11E-11 |  |  |  |  |  | 1 |  |  |  |  | 1 |  |  |  |
| cg13937905 |  |  |  | cg13937905 | RARG | 6.36E-10 |  |  |  |  |  | 1 |  |  |  |  | 1 |  |  |  |
| cg13951797 |  |  |  | cg13951797 | TRAF7 | 1.03E-08 |  |  |  |  |  | 1 |  |  |  |  | 1 |  |  |  |
| cg13985437 |  |  |  | cg13985437 | LRRC32 | 4.29E-09 |  |  |  |  |  | 1 |  |  |  |  | 1 |  |  |  |
| cg14014731 |  |  |  | cg14014731 | RPS6 | 9.09E-08 |  |  |  |  |  | 1 |  |  |  |  | 1 |  |  |  |
| cg14034325 |  |  |  | cg14034325 | HNRNPF | 2.24E-09 |  |  |  |  |  | 1 |  |  |  |  | 1 |  |  |  |
| cg14074174 |  |  |  | cg14074174 | SNAPC2 | 5.91E-09 |  |  |  |  |  | 1 |  |  |  |  | 1 |  |  |  |
| cg14120703 |  |  |  | cg14120703 | NOTCH1 | 1.48E-09 |  |  |  |  |  | 1 |  |  |  |  | 1 |  |  |  |
| cg14316231 |  |  |  | cg14316231 | MYST3 | 8.47E-12 |  |  |  |  |  | 1 |  |  |  |  | 1 |  |  |  |
| cg14571710 |  |  |  | cg14571710 | KIAA1949 | 1.23E-08 |  |  |  |  |  | 1 |  |  |  |  | 1 |  |  |  |
| cg14712058 |  |  |  | cg14712058 | SIN3B | 2.39E-10 |  |  |  |  |  | 1 |  |  |  |  | 1 |  |  |  |
| cg14796406 |  |  |  | cg14796406 | SEMA3C | 1.27E-10 |  |  |  |  |  | 1 |  |  |  |  | 1 |  |  |  |
| cg14975410 |  |  |  | cg14975410 | NA | 4.08E-08 |  |  |  |  |  | 1 |  |  |  |  | 1 |  |  |  |
| cg15036326 |  |  |  | cg15036326 | RPL23A | 6.11E-09 |  |  |  |  |  | 1 |  |  |  |  | 1 |  |  |  |
| cg15059065 |  |  |  | cg15059065 | NR2F6 | 1.16E-08 |  |  |  |  |  | 1 |  |  |  |  | 1 |  |  |  |
| cg15344028 |  |  |  | cg15344028 | ICOS | 5.67E-09 |  |  |  |  |  | 1 |  |  |  |  | 1 |  |  |  |
| cg15588941 |  |  |  | cg15588941 | SLC25A20 | 1.17E-08 |  |  |  |  |  | 1 |  |  |  |  | 1 |  |  |  |
| cg15677364 |  |  |  | cg15677364 | TUBB | 2.21E-09 |  |  |  |  |  | 1 |  |  |  |  | 1 |  |  |  |
| cg16047567 |  |  |  | cg16047567 | DHRS3 | 8.92E-16 |  |  |  |  |  | 1 |  |  |  |  | 1 |  |  |  |
| cg16219322 |  |  |  | cg16219322 | AHRR | 1.17E-12 |  |  |  |  |  | 1 |  |  |  |  | 1 |  |  |  |
| cg16290996 |  |  |  | cg16290996 | GAS5 | 2.48E-10 |  |  |  |  |  | 1 |  |  |  |  | 1 |  |  |  |
| cg16357582 |  |  |  | cg16357582 | CSRP1 | 9.33E-08 |  |  |  |  |  | 1 |  |  |  |  | 1 |  |  |  |
| cg16391678 |  |  |  | cg16391678 | ITGAL | 2.07E-13 |  |  |  |  |  | 1 |  |  |  |  | 1 |  |  |  |
| cg16398761 |  |  |  | cg16398761 | C14orf43 | 1.41E-11 |  |  |  |  |  | 1 |  |  |  |  | 1 |  |  |  |
| cg16519923 |  |  |  | cg16519923 | ITGAL | 1.24E-12 |  |  |  |  |  | 1 |  |  |  |  | 1 |  |  |  |
| cg16541026 |  |  |  | cg16541026 | P4HTM | 6.51E-10 |  |  |  |  |  | 1 |  |  |  |  | 1 |  |  |  |
| cg16646054 |  |  |  | cg16646054 | C5orf62 | 7.66E-08 |  |  |  |  |  | 1 |  |  |  |  | 1 |  |  |  |
| cg16702313 |  |  |  | cg16702313 | C14orf43 | 6.64E-08 |  |  |  |  |  | 1 |  |  |  |  | 1 |  |  |  |
| cg16708465 |  |  |  | cg16708465 | ABCC4 | 4.52E-08 |  |  |  |  |  | 1 |  |  |  |  | 1 |  |  |  |
| cg16736826 |  |  |  | cg16736826 | EDN2 | 6.38E-09 |  |  |  |  |  | 1 |  |  |  |  | 1 |  |  |  |
| cg16794579 |  |  |  | cg16794579 | XYLT1 | 2.66E-09 |  |  |  |  |  | 1 |  |  |  |  | 1 |  |  |  |
| cg16822666 |  |  |  | cg16822666 | ACACA | 1.25E-08 |  |  |  |  |  | 1 |  |  |  |  | 1 |  |  |  |
| cg17025683 |  |  |  | cg17025683 | SNORD78 | 1.03E-09 |  |  |  |  |  | 1 |  |  |  |  | 1 |  |  |  |
| cg17087741 |  |  |  | cg17087741 | NA | 7.47E-08 |  |  |  |  |  | 1 |  |  |  |  | 1 |  |  |  |
| cg17333042 |  |  |  | cg17333042 | KAZALD1 | 2.83E-08 |  |  |  |  |  | 1 |  |  |  |  | 1 |  |  |  |
| cg17417856 |  |  |  | cg17417856 | PRMT1 | 2.26E-09 |  |  |  |  |  | 1 |  |  |  |  | 1 |  |  |  |
| cg17478749 |  |  |  | cg17478749 | SNORA38 | 1.28E-08 |  |  |  |  |  | 1 |  |  |  |  | 1 |  |  |  |
| cg17551891 |  |  |  | cg17551891 | MAD1L1 | 1.46E-08 |  |  |  |  |  | 1 |  |  |  |  | 1 |  |  |  |
| cg17863923 |  |  |  | cg17863923 | RGPD1 | 9.99E-11 |  |  |  |  |  | 1 |  |  |  |  | 1 |  |  |  |
| cg17942851 |  |  |  | cg17942851 | C1orf113 | 1.92E-08 |  |  |  |  |  | 1 |  |  |  |  | 1 |  |  |  |
| cg18033416 |  |  |  | cg18033416 | RHBDL3 | 4.11E-09 |  |  |  |  |  | 1 |  |  |  |  | 1 |  |  |  |
| cg18236066 |  |  |  | cg18236066 | RPH3A | 3.78E-11 |  |  |  |  |  | 1 |  |  |  |  | 1 |  |  |  |
| cg18280057 |  |  |  | cg18280057 | MTSS1 | 1.47E-08 |  |  |  |  |  | 1 |  |  |  |  | 1 |  |  |  |
| cg18295744 |  |  |  | cg18295744 | ZMIZ1 | 3.52E-08 |  |  |  |  |  | 1 |  |  |  |  | 1 |  |  |  |
| cg18352916 |  |  |  | cg18352916 | CUTA | 1.14E-07 |  |  |  |  |  | 1 |  |  |  |  | 1 |  |  |  |
| cg18369034 |  |  |  | cg18369034 | PTGDR | 6.57E-08 |  |  |  |  |  | 1 |  |  |  |  | 1 |  |  |  |
| cg18405341 |  |  |  | cg18405341 | ATF4 | 3.68E-10 |  |  |  |  |  | 1 |  |  |  |  | 1 |  |  |  |
| cg18446336 |  |  |  | cg18446336 | GNA12 | 6.45E-11 |  |  |  |  |  | 1 |  |  |  |  | 1 |  |  |  |
| cg18474153 |  |  |  | cg18474153 | EEF2 | 8.69E-09 |  |  |  |  |  | 1 |  |  |  |  | 1 |  |  |  |
| cg18660898 |  |  |  | cg18660898 | CDC42SE1 | 1.73E-10 |  |  |  |  |  | 1 |  |  |  |  | 1 |  |  |  |
| cg18676273 |  |  |  | cg18676273 | INHBA | 7.35E-08 |  |  |  |  |  | 1 |  |  |  |  | 1 |  |  |  |
| cg18801806 |  |  |  | cg18801806 | CCDC84 | 2.48E-08 |  |  |  |  |  | 1 |  |  |  |  | 1 |  |  |  |
| cg18826637 |  |  |  | cg18826637 | NA | 4.14E-09 |  |  |  |  |  | 1 |  |  |  |  | 1 |  |  |  |
| cg18857467 |  |  |  | cg18857467 | HDAC1 | 9.49E-08 |  |  |  |  |  | 1 |  |  |  |  | 1 |  |  |  |
| cg19098932 |  |  |  | cg19098932 | PEX10 | 4.41E-08 |  |  |  |  |  | 1 |  |  |  |  | 1 |  |  |  |
| cg19197419 |  |  |  | cg19197419 | UBE2C | 1.42E-10 |  |  |  |  |  | 1 |  |  |  |  | 1 |  |  |  |
| cg19309676 |  |  |  | cg19309676 | C19orf76 | 2.45E-09 |  |  |  |  |  | 1 |  |  |  |  | 1 |  |  |  |
| cg19372602 |  |  |  | cg19372602 | NA | 6.51E-10 |  |  |  |  |  | 1 |  |  |  |  | 1 |  |  |  |
| cg19483007 |  |  |  | cg19483007 | WWTR1 | 1.10E-07 |  |  |  |  |  | 1 |  |  |  |  | 1 |  |  |  |
| cg19825437 |  |  |  | cg19825437 | NA | 6.16E-09 |  |  |  |  |  | 1 |  |  |  |  | 1 |  |  |  |
| cg19827923 |  |  |  | cg19827923 | GPR55 | 1.27E-11 |  |  |  |  |  | 1 |  |  |  |  | 1 |  |  |  |
| cg19868593 |  |  |  | cg19868593 | C6orf27 | 3.16E-08 |  |  |  |  |  | 1 |  |  |  |  | 1 |  |  |  |
| cg19918734 |  |  |  | cg19918734 | ME3 | 6.90E-09 |  |  |  |  |  | 1 |  |  |  |  | 1 |  |  |  |
| cg20059012 |  |  |  | cg20059012 | RARG | 1.32E-12 |  |  |  |  |  | 1 |  |  |  |  | 1 |  |  |  |
| cg20059928 |  |  |  | cg20059928 | NA | 2.75E-09 |  |  |  |  |  | 1 |  |  |  |  | 1 |  |  |  |
| cg20124610 |  |  |  | cg20124610 | CARS2 | 4.35E-12 |  |  |  |  |  | 1 |  |  |  |  | 1 |  |  |  |
| cg20131596 |  |  |  | cg20131596 | PAM | 6.19E-09 |  |  |  |  |  | 1 |  |  |  |  | 1 |  |  |  |
| cg20188739 |  |  |  | cg20188739 | SNORD87 | 4.12E-08 |  |  |  |  |  | 1 |  |  |  |  | 1 |  |  |  |
| cg20303561 |  |  |  | cg20303561 | CCDC88C | 3.14E-09 |  |  |  |  |  | 1 |  |  |  |  | 1 |  |  |  |
| cg20322193 |  |  |  | cg20322193 | RALA | 5.40E-08 |  |  |  |  |  | 1 |  |  |  |  | 1 |  |  |  |
| cg20477259 |  |  |  | cg20477259 | TNF | 4.53E-08 |  |  |  |  |  | 1 |  |  |  |  | 1 |  |  |  |
| cg20727233 |  |  |  | cg20727233 | ASNS | 8.88E-10 |  |  |  |  |  | 1 |  |  |  |  | 1 |  |  |  |
| cg20813374 |  |  |  | cg20813374 | FKBP5 | 1.17E-08 |  |  |  |  |  | 1 |  |  |  |  | 1 |  |  |  |
| cg20886049 |  |  |  | cg20886049 | TSKU | 2.48E-09 |  |  |  |  |  | 1 |  |  |  |  | 1 |  |  |  |
| cg20912205 |  |  |  | cg20912205 | NAT6 | 6.39E-08 |  |  |  |  |  | 1 |  |  |  |  | 1 |  |  |  |
| cg21091547 |  |  |  | cg21091547 | CDKN1A | 1.69E-09 |  |  |  |  |  | 1 |  |  |  |  | 1 |  |  |  |
| cg21201109 |  |  |  | cg21201109 | KANK2 | 1.86E-08 |  |  |  |  |  | 1 |  |  |  |  | 1 |  |  |  |
| cg21201657 |  |  |  | cg21201657 | SAFB | 2.30E-09 |  |  |  |  |  | 1 |  |  |  |  | 1 |  |  |  |
| cg21445230 |  |  |  | cg21445230 | PSMB10 | 8.96E-09 |  |  |  |  |  | 1 |  |  |  |  | 1 |  |  |  |
| cg21446172 |  |  |  | cg21446172 | CAPN8 | 8.78E-13 |  |  |  |  |  | 1 |  |  |  |  | 1 |  |  |  |
| cg21500300 |  |  |  | cg21500300 | BCAT1 | 3.12E-08 |  |  |  |  |  | 1 |  |  |  |  | 1 |  |  |  |
| cg21618521 |  |  |  | cg21618521 | B3GALT4 | 1.63E-08 |  |  |  |  |  | 1 |  |  |  |  | 1 |  |  |  |
| cg21698310 |  |  |  | cg21698310 | PPP1R9B | 3.21E-09 |  |  |  |  |  | 1 |  |  |  |  | 1 |  |  |  |
| cg21746120 |  |  |  | cg21746120 | LRP5 | 4.52E-09 |  |  |  |  |  | 1 |  |  |  |  | 1 |  |  |  |
| cg21747070 |  |  |  | cg21747070 | NA | 8.14E-08 |  |  |  |  |  | 1 |  |  |  |  | 1 |  |  |  |
| cg22407942 |  |  |  | cg22407942 | SNORD93 | 7.09E-09 |  |  |  |  |  | 1 |  |  |  |  | 1 |  |  |  |
| cg22441770 |  |  |  | cg22441770 | CRTC2 | 7.78E-11 |  |  |  |  |  | 1 |  |  |  |  | 1 |  |  |  |
| cg22499893 |  |  |  | cg22499893 | SFRS13A | 7.53E-08 |  |  |  |  |  | 1 |  |  |  |  | 1 |  |  |  |
| cg22549408 |  |  |  | cg22549408 | PMAIP1 | 4.37E-08 |  |  |  |  |  | 1 |  |  |  |  | 1 |  |  |  |
| cg22628926 |  |  |  | cg22628926 | SLC25A11 | 4.80E-08 |  |  |  |  |  | 1 |  |  |  |  | 1 |  |  |  |
| cg22740603 |  |  |  | cg22740603 | SYNGAP1 | 1.49E-08 |  |  |  |  |  | 1 |  |  |  |  | 1 |  |  |  |
| cg22851200 |  |  |  | cg22851200 | TRIP6 | 1.40E-10 |  |  |  |  |  | 1 |  |  |  |  | 1 |  |  |  |
| cg22870429 |  |  |  | cg22870429 | TIGIT | 4.15E-09 |  |  |  |  |  | 1 |  |  |  |  | 1 |  |  |  |
| cg22904711 |  |  |  | cg22904711 | KCNN4 | 2.08E-10 |  |  |  |  |  | 1 |  |  |  |  | 1 |  |  |  |
| cg22950598 |  |  |  | cg22950598 | GDF11 | 8.96E-12 |  |  |  |  |  | 1 |  |  |  |  | 1 |  |  |  |
| cg22998476 |  |  |  | cg22998476 | NA | 4.28E-08 |  |  |  |  |  | 1 |  |  |  |  | 1 |  |  |  |
| cg23233742 |  |  |  | cg23233742 | NA | 3.02E-13 |  |  |  |  |  | 1 |  |  |  |  | 1 |  |  |  |
| cg23565821 |  |  |  | cg23565821 | CUTA | 4.78E-11 |  |  |  |  |  | 1 |  |  |  |  | 1 |  |  |  |
| cg23574443 |  |  |  | cg23574443 | LOC100128288 | 3.86E-08 |  |  |  |  |  | 1 |  |  |  |  | 1 |  |  |  |
| cg23892690 |  |  |  | cg23892690 | BZW1 | 9.24E-08 |  |  |  |  |  | 1 |  |  |  |  | 1 |  |  |  |
| cg23902550 |  |  |  | cg23902550 | EEF1D | 6.02E-08 |  |  |  |  |  | 1 |  |  |  |  | 1 |  |  |  |
| cg23928726 |  |  |  | cg23928726 | PEX10 | 5.73E-08 |  |  |  |  |  | 1 |  |  |  |  | 1 |  |  |  |
| cg24086068 |  |  |  | cg24086068 | SHROOM3 | 2.52E-08 |  |  |  |  |  | 1 |  |  |  |  | 1 |  |  |  |
| cg24139443 |  |  |  | cg24139443 | NA | 6.83E-08 |  |  |  |  |  | 1 |  |  |  |  | 1 |  |  |  |
| cg24287110 |  |  |  | cg24287110 | KLF6 | 5.29E-10 |  |  |  |  |  | 1 |  |  |  |  | 1 |  |  |  |
| cg24323726 |  |  |  | cg24323726 | ZBED2 | 2.16E-09 |  |  |  |  |  | 1 |  |  |  |  | 1 |  |  |  |
| cg24342283 |  |  |  | cg24342283 | CXCR5 | 7.30E-11 |  |  |  |  |  | 1 |  |  |  |  | 1 |  |  |  |
| cg24389054 |  |  |  | cg24389054 | HOXA7 | 2.12E-08 |  |  |  |  |  | 1 |  |  |  |  | 1 |  |  |  |
| cg24688690 |  |  |  | cg24688690 | AHRR | 1.30E-10 |  |  |  |  |  | 1 |  |  |  |  | 1 |  |  |  |
| cg24719910 |  |  |  | cg24719910 | TGFBR2 | 2.64E-09 |  |  |  |  |  | 1 |  |  |  |  | 1 |  |  |  |
| cg24741744 |  |  |  | cg24741744 | NA | 4.19E-08 |  |  |  |  |  | 1 |  |  |  |  | 1 |  |  |  |
| cg24931658 |  |  |  | cg24931658 | EPB49 | 1.85E-09 |  |  |  |  |  | 1 |  |  |  |  | 1 |  |  |  |
| cg25007705 |  |  |  | cg25007705 | ECE1 | 4.98E-08 |  |  |  |  |  | 1 |  |  |  |  | 1 |  |  |  |
| cg25103895 |  |  |  | cg25103895 | ADO | 5.84E-08 |  |  |  |  |  | 1 |  |  |  |  | 1 |  |  |  |
| cg25212025 |  |  |  | cg25212025 | PARD3 | 2.57E-09 |  |  |  |  |  | 1 |  |  |  |  | 1 |  |  |  |
| cg25292882 |  |  |  | cg25292882 | NA | 7.86E-17 |  |  |  |  |  | 1 |  |  |  |  | 1 |  |  |  |
| cg25323554 |  |  |  | cg25323554 | C17orf63 | 8.96E-15 |  |  |  |  |  | 1 |  |  |  |  | 1 |  |  |  |
| cg25451120 |  |  |  | cg25451120 | ABI3 | 8.68E-08 |  |  |  |  |  | 1 |  |  |  |  | 1 |  |  |  |
| cg25507845 |  |  |  | cg25507845 | CAMK2A | 3.44E-08 |  |  |  |  |  | 1 |  |  |  |  | 1 |  |  |  |
| cg25560398 |  |  |  | cg25560398 | ECEL1P2 | 2.77E-10 |  |  |  |  |  | 1 |  |  |  |  | 1 |  |  |  |
| cg25596754 |  |  |  | cg25596754 | TNXB | 6.03E-08 |  |  |  |  |  | 1 |  |  |  |  | 1 |  |  |  |
| cg25799109 |  |  |  | cg25799109 | ARHGEF3 | 1.09E-07 |  |  |  |  |  | 1 |  |  |  |  | 1 |  |  |  |
| cg25998745 |  |  |  | cg25998745 | NA | 1.06E-07 |  |  |  |  |  | 1 |  |  |  |  | 1 |  |  |  |
| cg26158528 |  |  |  | cg26158528 | SESN2 | 8.94E-08 |  |  |  |  |  | 1 |  |  |  |  | 1 |  |  |  |
| cg26196424 |  |  |  | cg26196424 | C14orf37 | 1.32E-09 |  |  |  |  |  | 1 |  |  |  |  | 1 |  |  |  |
| cg26203136 |  |  |  | cg26203136 | PRKAR1B | 1.23E-08 |  |  |  |  |  | 1 |  |  |  |  | 1 |  |  |  |
| cg26337070 |  |  |  | cg26337070 | ATOH8 | 7.90E-09 |  |  |  |  |  | 1 |  |  |  |  | 1 |  |  |  |
| cg26470501 |  |  |  | cg26470501 | BCL3 | 7.79E-08 |  |  |  |  |  | 1 |  |  |  |  | 1 |  |  |  |
| cg26529655 |  |  |  | cg26529655 | AHRR | 2.87E-11 |  |  |  |  |  | 1 |  |  |  |  | 1 |  |  |  |
| cg26703507 |  |  |  | cg26703507 | SLC20A1 | 1.12E-09 |  |  |  |  |  | 1 |  |  |  |  | 1 |  |  |  |
| cg26718213 |  |  |  | cg26718213 | SNED1 | 7.66E-08 |  |  |  |  |  | 1 |  |  |  |  | 1 |  |  |  |
| cg26729380 |  |  |  | cg26729380 | TNF | 4.65E-09 |  |  |  |  |  | 1 |  |  |  |  | 1 |  |  |  |
| cg26764244 |  |  |  | cg26764244 | GNG12 | 2.28E-17 |  |  |  |  |  | 1 |  |  |  |  | 1 |  |  |  |
| cg26856289 |  |  |  | cg26856289 | SFRS13A | 2.18E-13 |  |  |  |  |  | 1 |  |  |  |  | 1 |  |  |  |
| cg26905258 |  |  |  | cg26905258 | MYCBP2 | 1.46E-14 |  |  |  |  |  | 1 |  |  |  |  | 1 |  |  |  |
| cg26995224 |  |  |  | cg26995224 | KDM2B | 6.46E-10 |  |  |  |  |  | 1 |  |  |  |  | 1 |  |  |  |
| cg27449150 |  |  |  | cg27449150 | NA | 1.34E-08 |  |  |  |  |  | 1 |  |  |  |  | 1 |  |  |  |
| cg27514333 |  |  |  | cg27514333 | SMAD6 | 1.29E-09 |  |  |  |  |  | 1 |  |  |  |  | 1 |  |  |  |
| cg27598208 |  |  |  | cg27598208 | TCEA2 | 5.55E-08 |  |  |  |  |  | 1 |  |  |  |  | 1 |  |  |  |
