# Supplementary material for: Tobacco smoking differently influences cell types of the innate and adaptive immune system—indications from CpG site methylation
Source: Clin Epigenetics. 2016 Aug 3;8:83. doi: 10.1186/s13148-016-0249-7 (PMC4973040; doi:10.1186/s13148-016-0249-7)
Supplement: Additional file 2: — Estimation of the prominent cell type of whole blood accounting for major smoking-associated methylation change (∆meth) at single CpG site based on reports of methylation changes in whole blood and peripheral blood mononuclear cells (PBMC). (DOCX 20 kb) [file 13148_2016_249_MOESM2_ESM.docx]

**Table S2.** Estimation of the prominent cell type of blood accounting for major smoking-associated methylation change (∆meth) at 24 intersectional single CpG site (as indicated in figure 1) based on one reports of methylation changes in WBC and one report in peripheral blood mononuclear cells (PBMC).

|  |  | *Zeilinger et al.* | *Dogan et al.* | Estimated | Difference of | Proposed cell type of |  |  |  |
| --- | --- | --- | --- | --- | --- | --- | --- | --- | --- |
|  |  | WBC | PBMC | ∆meth in | ∆meth | main ∆meth |  |  |  |
| CpG* | Gene | ∆meth^§^ | ∆meth^§^ | granulocyte^#^ | gran - PBMC | in WBC |  |  |  |
|  |  |  |  |  |  |  |  |  |  |
| cg12806681 | *AHRR* | -2 | -4 | -1 | 3 | PBMC |  |  |  |
| cg03991871 | *AHRR* | -6 | -6 | -6 | 0 |  |  |  |  |
| cg23916896 | *AHRR* | -2 | -6 | 0 | 6 | PBMC |  |  |  |
| cg01899089 | *AHRR* | -3 | -6 | -1 | 5 | PBMC |  |  |  |
| **cg05575921*** | *AHRR* | -24 | -15 | -31 | -16 | granulocyte |  |  |  |
| cg26703534 | *AHRR* | -6 | -6 | -6 | 0 |  |  |  |  |
| cg25648203 | *AHRR* | -8 | -5 | -10 | -5 | granulocyte |  |  |  |
| cg21161138 | *AHRR* | -10 | -8 | -12 | -4 | granulocyte |  |  |  |
| cg21566642 | *ALPPL2b* | -17 | -10 | -21 | -11 | granulocyte |  |  |  |
| cg01940273 | *ALPPL2b* | -8 | -9 | -7 | 2 |  |  |  |  |
| cg05951221 | *ALPPL2b* | -5 | -8 | -3 | 5 | PBMC |  |  |  |
| cg03329539 | *ALPPL2b* | -2 | -5 | -1 | 4 | PBMC |  |  |  |
| cg22851561 | *C14orf43* | -6 | -7 | -5 | 2 |  |  |  |  |
| cg01731783 | *C14orf43* | -2 | -5 | 0 | 5 | PBMC |  |  |  |
| **cg02657160*** | *CPOX* | -1 | -6 | 2 | 8 | PBMC |  |  |  |
| **cg03636183*** | *F2RL3* | -15 | -11 | -17 | -6 | granulocyte |  |  |  |
| **cg09935388*** | *GFI1* | -15 | -12 | -18 | -6 | granulocyte |  |  |  |
| cg12876356 | *GFI1* | -8 | -9 | -8 | 1 |  |  |  |  |
| **cg19859270*** | *GPR15* | -1 | -10 | 4 | 14 | PBMC |  |  |  |
| cg12075928 | *PTK2* | -8 | -7 | -8 | -1 |  |  |  |  |
| cg19572487 | *RARA* | -10 | -7 | -12 | -5 | granulocyte |  |  |  |
| cg06126421 | xa | -17 | -11 | -21 | -10 | granulocyte |  |  |  |
| cg24859433 | xa | -4 | -4 | -4 | 0 |  |  |  |  |
| cg04885881 | xa | -7 | -8 | -7 | 1 |  |  |  |  |
|  |  |  |  |  |  |  |  |  |  |
| *, selected CpG for verification of distribution of ∆meth among different cell types of WBC  ^§^, delta-methylation between tobacco smoker versus non-smoker  ^#^, considering a content of about 60% granulocytes and 40% PBMC in WBC, ∆meth in granulocytes was calculated  by (∆methWBC - 0.4*∆methPBMC)/0.6  xa, non annotated gene | | | | | | |  |  |  |
|  | | | |  |  |  |  |  |  |
|  | | | | | | |  |  |  |
