# Supplementary figures and images for: Tobacco smoking differently influences cell types of the innate and adaptive immune system—indications from CpG site methylation
Source: Clin Epigenetics. 2016 Aug 3;8:83. doi: 10.1186/s13148-016-0249-7 (PMC4973040; doi:10.1186/s13148-016-0249-7)

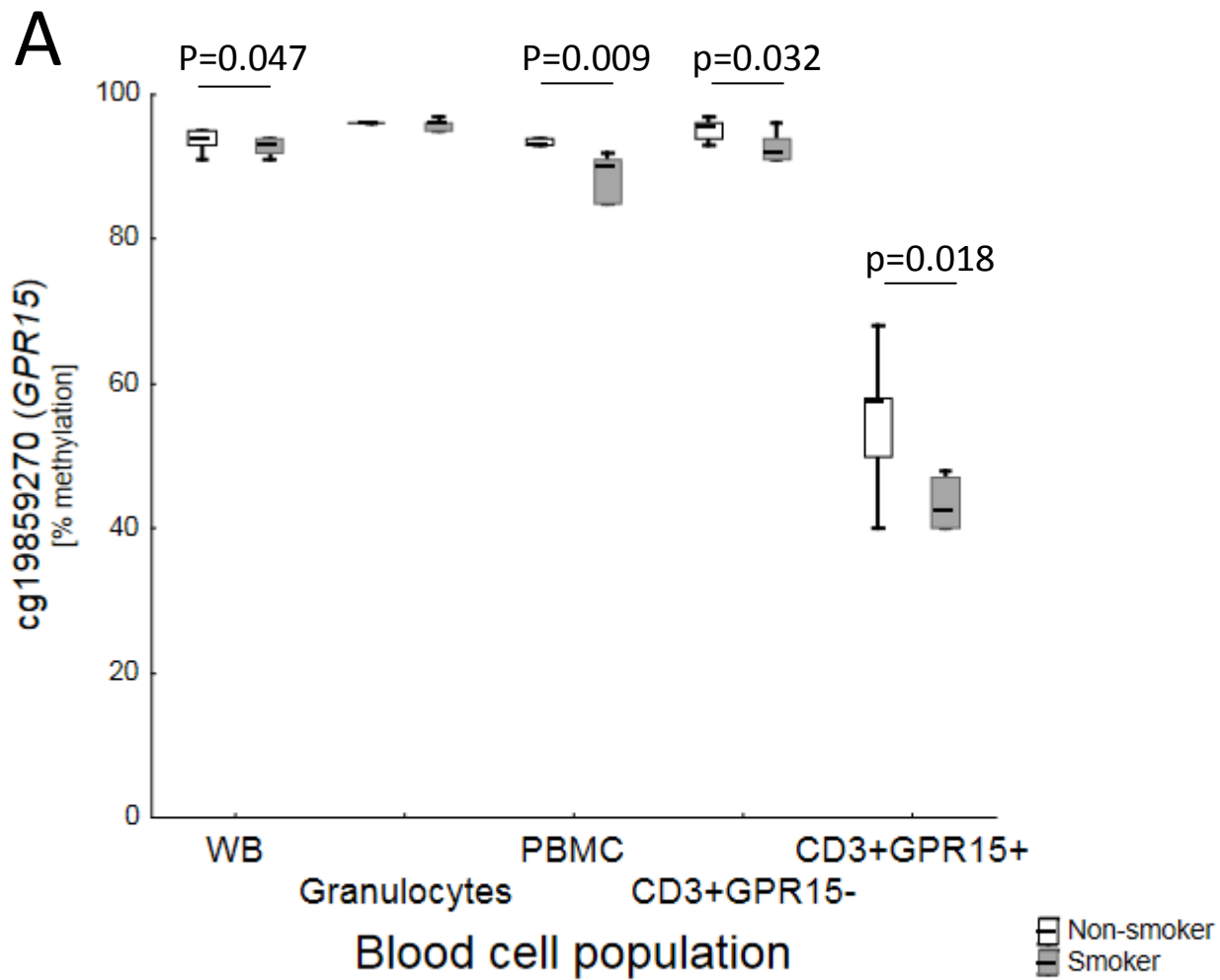

Figure S4

**B**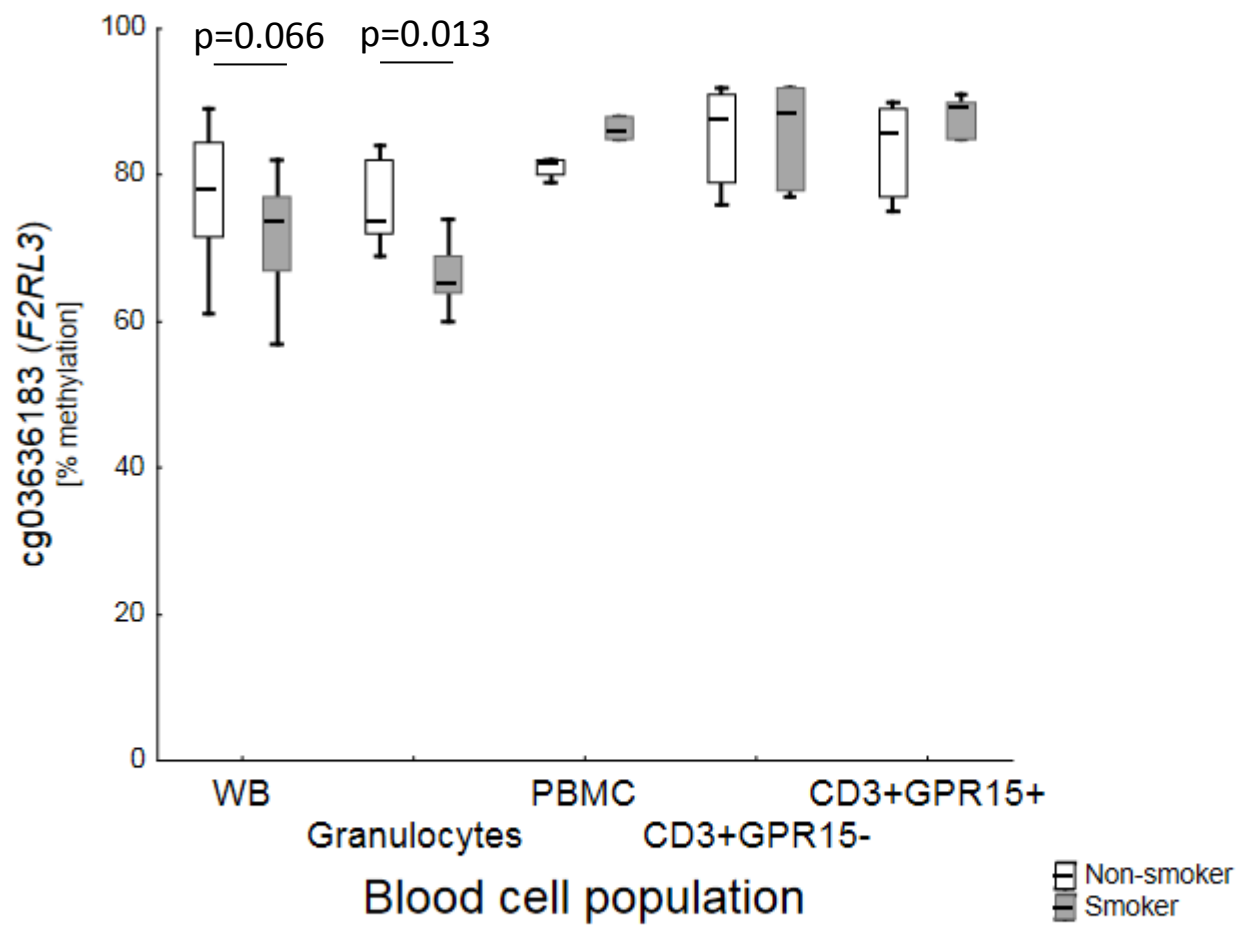

Figure S4

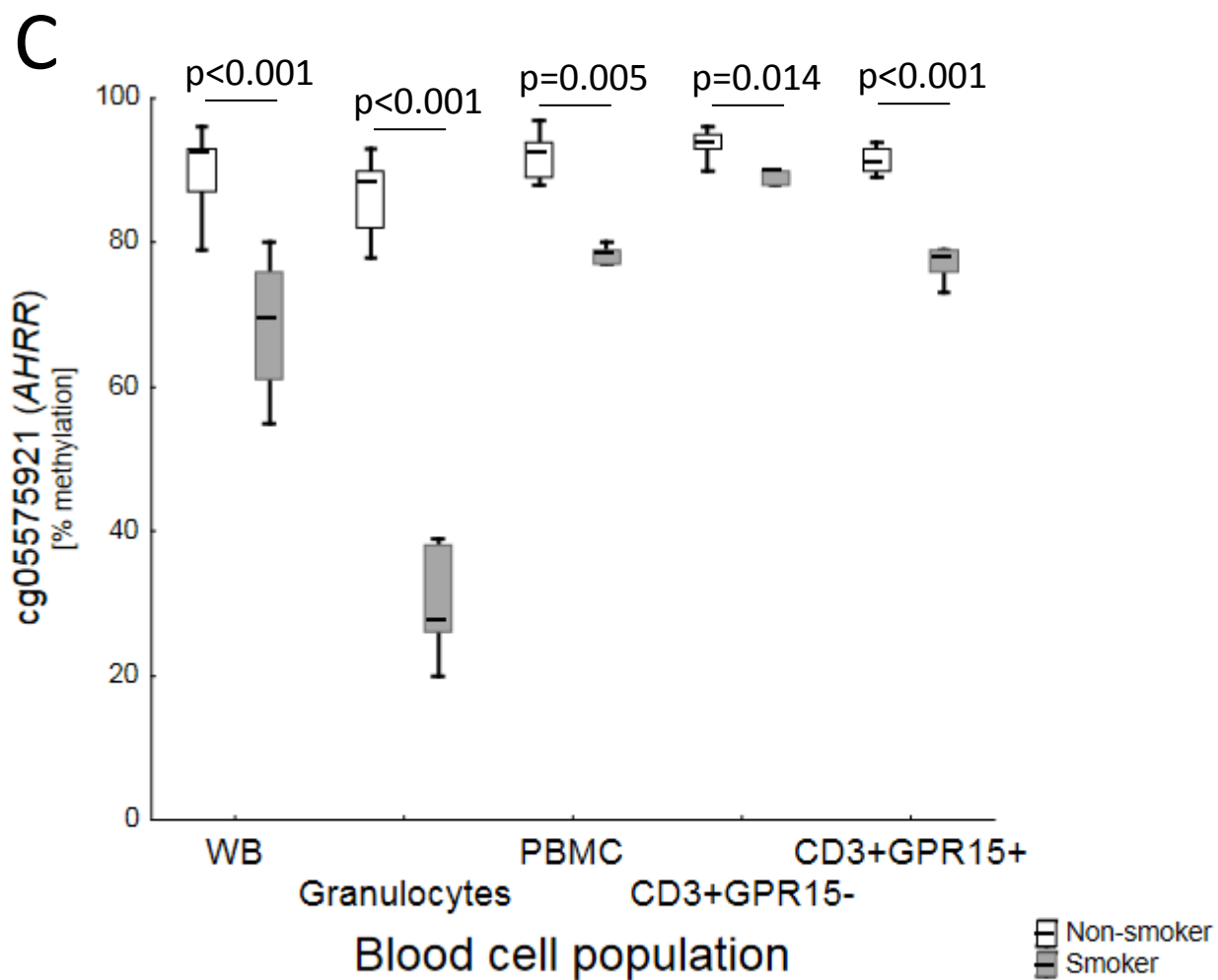

Figure S4

Supplement: Additional file 3: — Distribution of methylation changes at single CpGs by tobacco smoking in different cell populations of whole blood. Highlighted are significant methylation changes. NS, non-smoker; S, smoker; p, P, statistical significance by Student’s t test (p) or Mann-Whitney U test (P). (PDF 81 kb) [file 13148_2016_249_MOESM3_ESM.pdf]

A

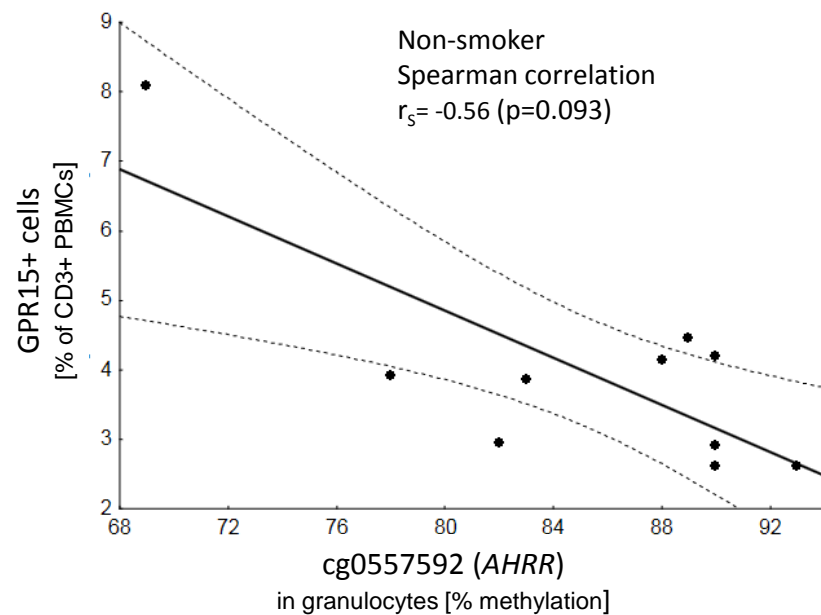

B

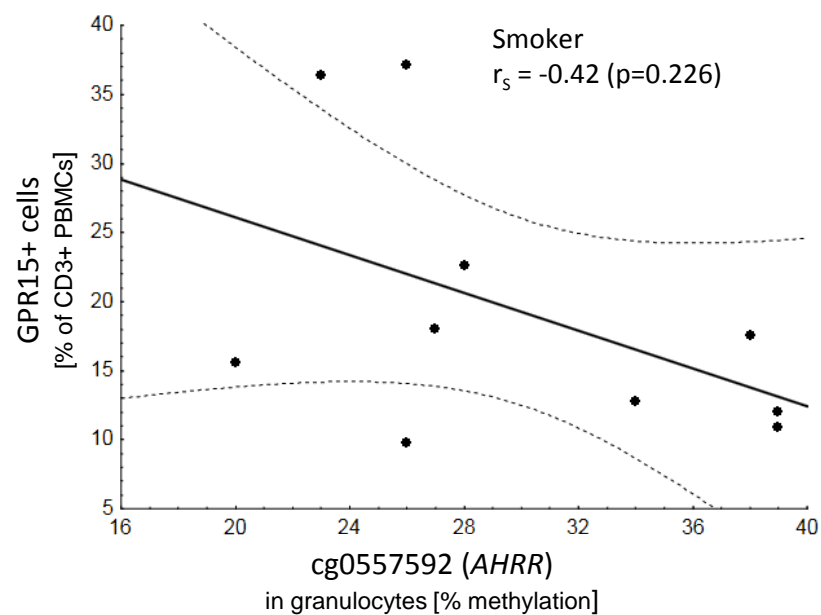

Figure S3

Supplement: Additional file 4: — Spearman correlation between amount of GPR15+ T cells and methylation at cg05575921 in granulocytes in non-smokers and smokers. Both biomarkers did not correlate significantly with each other. (PDF 101 kb) [file 13148_2016_249_MOESM4_ESM.pdf]

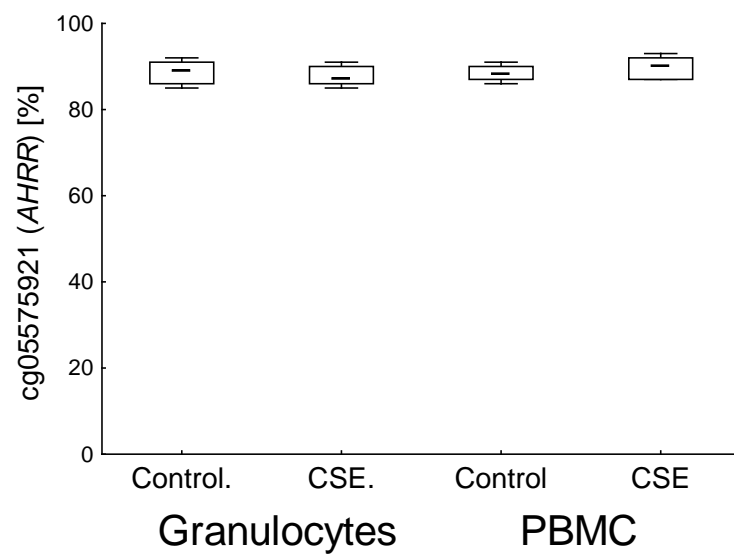

Figure S2

Supplement: Additional file 5: — Figure S2. Influence of cigarette smoke extract (CSE) exposure on in vitro cultured PBMC and granulocytes (48 h) of non-smokers (n = 3, each) on methylation at cg05575921 (AHRR). CSE did not change the methylation at cg05575921. (PDF 27 kb) [file 13148_2016_249_MOESM5_ESM.pdf]
