# Supplementary material for: Tobacco smoking differently influences cell types of the innate and adaptive immune system—indications from CpG site methylation
Source: Clin Epigenetics. 2016 Aug 3;8:83. doi: 10.1186/s13148-016-0249-7 (PMC4973040; doi:10.1186/s13148-016-0249-7)
Supplement: Additional file 6: — Venn diagram illustrating intersections of tobacco smoking-evoked methylation changes at single CpGs of four different reports using genomic DNA isolated from whole blood (WBC), from separated peripheral blood mononuclear cells (PBMC) of blood or from cells collected from the inside of a person’s cheek (buccal cells) of adult probands. Origin of data sets is indicated. (DOCX 20 kb) [file 13148_2016_249_MOESM6_ESM.docx]

**Table S3.** Estimation of the prominent cell type of blood accounting for major smoking-associated methylation change (∆meth) at 24 intersectional single CpG site (as indicated in figure 1) based on one reports of methylation changes in WBC and one report in peripheral blood mononuclear cells (PBMC).

|  |  | *Zeilinger et al.* | *Dogan et al.* | Estimated | Difference of | Proposed cell type of |  |  |  |
| --- | --- | --- | --- | --- | --- | --- | --- | --- | --- |
|  |  | WBC | PBMC | ∆meth in | ∆meth | main ∆meth |  |  |  |
| CpG* | Gene | ∆meth^§^ | ∆meth^§^ | granulocyte^#^ | gran - PBMC | in WBC |  |  |  |
|  |  |  |  |  |  |  |  |  |  |
| cg12806681 | *AHRR* | -2 | -4 | -1 | 3 | PBMC |  |  |  |
| cg03991871 | *AHRR* | -6 | -6 | -6 | 0 |  |  |  |  |
| cg23916896 | *AHRR* | -2 | -6 | 0 | 6 | PBMC |  |  |  |
| cg01899089 | *AHRR* | -3 | -6 | -1 | 5 | PBMC |  |  |  |
| **cg05575921*** | *AHRR* | -24 | -15 | -31 | -16 | granulocyte |  |  |  |
| cg26703534 | *AHRR* | -6 | -6 | -6 | 0 |  |  |  |  |
| cg25648203 | *AHRR* | -8 | -5 | -10 | -5 | granulocyte |  |  |  |
| cg21161138 | *AHRR* | -10 | -8 | -12 | -4 | granulocyte |  |  |  |
| cg21566642 | *ALPPL2b* | -17 | -10 | -21 | -11 | granulocyte |  |  |  |
| cg01940273 | *ALPPL2b* | -8 | -9 | -7 | 2 |  |  |  |  |
| cg05951221 | *ALPPL2b* | -5 | -8 | -3 | 5 | PBMC |  |  |  |
| cg03329539 | *ALPPL2b* | -2 | -5 | -1 | 4 | PBMC |  |  |  |
| cg22851561 | *C14orf43* | -6 | -7 | -5 | 2 |  |  |  |  |
| cg01731783 | *C14orf43* | -2 | -5 | 0 | 5 | PBMC |  |  |  |
| **cg02657160*** | *CPOX* | -1 | -6 | 2 | 8 | PBMC |  |  |  |
| **cg03636183*** | *F2RL3* | -15 | -11 | -17 | -6 | granulocyte |  |  |  |
| **cg09935388*** | *GFI1* | -15 | -12 | -18 | -6 | granulocyte |  |  |  |
| cg12876356 | *GFI1* | -8 | -9 | -8 | 1 |  |  |  |  |
| **cg19859270*** | *GPR15* | -1 | -10 | 4 | 14 | PBMC |  |  |  |
| cg12075928 | *PTK2* | -8 | -7 | -8 | -1 |  |  |  |  |
| cg19572487 | *RARA* | -10 | -7 | -12 | -5 | granulocyte |  |  |  |
| cg06126421 | xa | -17 | -11 | -21 | -10 | granulocyte |  |  |  |
| cg24859433 | xa | -4 | -4 | -4 | 0 |  |  |  |  |
| cg04885881 | xa | -7 | -8 | -7 | 1 |  |  |  |  |
|  |  |  |  |  |  |  |  |  |  |
| *, selected CpG for verification of distribution of ∆meth among different cell types of WBC  ^§^, delta-methylation between tobacco smoker versus non-smoker  ^#^, considering a content of about 60% granulocytes and 40% PBMC in WBC, ∆meth in granulocytes was calculated  by (∆methWBC - 0.4*∆methPBMC)/0.6  xa, non annotated gene | | | | | | |  |  |  |
|  | | | |  |  |  |  |  |  |
|  | | | | | | |  |  |  |
