# Supplementary material for: Tobacco smoking differently influences cell types of the innate and adaptive immune system—indications from CpG site methylation
Source: Clin Epigenetics. 2016 Aug 3;8:83. doi: 10.1186/s13148-016-0249-7 (PMC4973040; doi:10.1186/s13148-016-0249-7)
Supplement: Additional file 7: — Sample sizes of the prospective birth cohort study LINA. (DOCX 16 kb) [file 13148_2016_249_MOESM7_ESM.docx]

**Table S4.** Sample sizes of the prospective birth cohort study LINA.

| *Age of child* | *Mother-child pairs [n]* | *Blood sample [n]* | | *DNA (blood)* | |
| --- | --- | --- | --- | --- | --- |
| *[years]* |  | *Mother* | *Child* | *Mother* | *Child* |
|  |  |  |  |  |  |
| Birth/Prenatal | 629 | 622 (PW 34) | 518 (CB) | 619 (PW 34) | 473 (CB) |
| 1 | 606 | 564 | 517 | 550 | 499 |
| 2 | 546 | 397 | 340 | 386 | 331 |
| 3 | 514 | 367 | 289 | 360 | 289 |
| 4 | 474 | 333 | 254 | 324 | 244 |
|  |  |  |  |  |  |
|  | *Tobacco smoking* | *Pyrosequencing for cg05575921* | | | |
|  | *[urine: >100 µg cotinine/g creatinine]* | *Non-smoker* | | *Smoker* | |
|  |  | *Mother* | *Child* | *Mother* | *Child* |
|  |  |  |  |  |  |
| Birth/Prenatal | 29 (PW 34) | 20 (PW 34) | 20 (CB) | 13 (PW 34) | 13 (CB) |
| 1 |  | 20 | 20 | 13 | 13 |
| 2 |  |  | 20 |  | 11 |
| 3 |  |  |  |  |  |
| 4 |  |  | 20 |  | 8 |
|  |  |  |  |  |  |
| *PW34, pregnancy week; CB, cord blood* | | |  |  |  |
