# Supplementary material for: Tobacco smoking differently influences cell types of the innate and adaptive immune system—indications from CpG site methylation
Source: Clin Epigenetics. 2016 Aug 3;8:83. doi: 10.1186/s13148-016-0249-7 (PMC4973040; doi:10.1186/s13148-016-0249-7)
Supplement: Additional file 8: — Primer for pyrosequencing. (DOCX 15 kb) [file 13148_2016_249_MOESM8_ESM.docx]

**Table S1**. Primer for pyrosequencing.

| CpG | Gene | Primer | Sequence |
| --- | --- | --- | --- |
|  |  |  |  |
| cg05575921 | *AHRR* | F1 | GTGGGGATTGTTTATTTTTGAGAGG |
|  |  | R1 | [Btn]AACCCTACCAAAACCACTC |
|  |  | S1 | GGTTTTGGTTTTGTTTTGTA |
| cg02657160 | *CPOX* | F1 | GGTTTAGTGGTTTTTGTTTGAAAATATAAG |
|  |  | R1 | [Btn]AACCCCACATCTTAAATAAAAACTT |
|  |  | S1 | GTTAGTATTATAGTTTAAAATTTGT |
| cg03636183 | *F2RL3* | F1 | TTGGGTTGGGTGTTTATTAGG |
|  |  | R1 | [Btn]AACAACCCCAAAACCAACAAAAAATCAA |
|  |  | S1 | GTTGGAGTTGTGGGTG |
| cg09935388 | *GFI1* | F1 | TGGTTATTTTAGTGAGAGGTTGTAT |
|  |  | R1 | [Btn]CACCCCTCCCACAATCAATAAATTAACTT |
|  |  | S1 | GTTTAGTTAGGAGGGGTT |
| cg19859270 | *GPR15* | F1 | [Btn]GTTATTTGGTTATTGTGTGGTTAGT |
|  |  | R1 | CCTTTTCTCTACACAATATAACTTATCAT |
|  |  | S1 | CACAATCTATCCTTCTAAATTT |
| cg13086586 | *PAICS* | F1 | TAGGAGGTTGAGGTAGGAGAAT |
|  |  | R1 | [Btn]ACTTACACACACCCCCCCTAAACAAAT |
|  |  | S1 | GTTATTGTATTTTAGTTTGGG |
| cg02319016 | *PAK2* | F1 | GGTGGTAGGAGTTTGTAGTTTTAGTTATT |
|  |  | R1 | [Btn]ACTTAAAATCTTAAAATTAACCCAATTTCT |
|  |  | S1 | TGTATTTTAGTTTGGGTTATAGAG |
